# Supplementary material for: Differences in local population history at the finest level: the case of the Estonian population
Source: Eur J Hum Genet. 2020 Jul 25;28(11):1580–91. doi: 10.1038/s41431-020-0699-4 (PMC7575549; doi:10.1038/s41431-020-0699-4)
Supplement: Supplementary file 2 — Supplementary table [file 41431_2020_699_MOESM2_ESM.docx]

**Title:** Differences in local population history at the finest level: the case of the Estonian population

**Running title:** Genetic structure of Estonia

**Keywords:** Genetic structure, demographic history, natural selection

*Vasili Pankratov^a,*^, Francesco Montinaro^a^, Alena Kushniarevich^a^, Georgi Hudjashov^a,b^, Flora Jay^c^, Lauri Saag^a^, Rodrigo Flores^a^, Davide Marnetto^a^, Marten Seppel*^d^*, Mart Kals^a^, Urmo Võsa^a^, Cristian Taccioli^e^, Märt Möls^f^, Lili Milani^a^, Anto Aasa^g^, Daniel John Lawson^h^, Tõnu Esko^a^, Reedik Mägi^a^, Luca Pagani^a,e,1^, Andres Metspalu^a,1^, Mait Metspalu^a,1^*

^a^Institute of Genomics, University of Tartu, Tartu, 51010, Estonia;

^b^Statistics and Bioinformatics Group, School of Fundamental Sciences, Massey University, Palmerston North 4474, New Zealand;

^c^Laboratoire de Recherche en Informatique, CNRS UMR 8623, Université Paris-Sud, Université Paris-Saclay, Orsay 91405, France

^d^Institute of History and Archaeology, University of Tartu, Tartu 51005, Estonia;

^e^ Department of Biology, University of Padova, Padova 35131, Italy;

^f^Institute of Mathematical Statistics, University of Tartu, Tartu 50409, Estonia;

^g^Institute of Geography University of Tartu, Tartu 51003, Estonia;

^h^Medical Research Council Integrative Epidemiology Unit, Department of Population Health Sciences, Bristol Medical School, University of Bristol, Bristol BS8 2BN, United Kingdom;

*Corresponding author vasilipankratov@gmail.com

^1^Contributed equally

**Abstract**

Several recent studies detected fine-scale genetic structure in human populations. Hence, groups conventionally treated as single populations harbour significant variation in terms of allele frequencies and patterns of haplotype sharing. It has been shown that these findings should be considered when performing studies of genetic associations and natural selection, especially when dealing with polygenic phenotypes. However, there is little understanding of the practical effects of such genetic structure on demography reconstructions and selection scans when focusing on recent population history. Here we tested the impact of population structure on such inferences using high-coverage (~30X) genome sequences of 2,305 Estonians. We show that different regions of Estonia differ in both effective population size dynamics and signatures of natural selection. By analyzing identity-by-descent segments we also reveal that some Estonian regions exhibit evidence of a bottleneck 10-15 generations ago reflecting sequential episodes of wars, plague, and famine, although this signal is virtually undetected when treating Estonia as a single population. Besides that, we provide a framework for relating effective population size estimated from genetic data to actual census size and validate it on the Estonian population. This approach may be widely used both to cross-check estimates based on historical sources as well as to get insight into times and/or regions with no other information available. Our results suggest that the history of human populations within the last few millennia can be highly region-specific and cannot be properly studied without taking local genetic structure into account.

**Introduction**

With more and more datasets including genetic data from hundreds and thousands individuals now available it becomes apparent that most if not all human populations exhibit at least some degree of geography-driven genetic structure even at small scales (for some examples see^1–5^). Such structure is worthy of attention first of all because it may have confounding effects on genetic inference: a number of studies have highlighted the fact that not accounting for genetic structure even in datasets representing one nation or ethnic group may give false-positive results when studying genetic associations and natural selection signals, especially in the case of polygenic phenotypes^6–9^. However, our understanding of the effects of structure on population genetic analysis is still incomplete. One of the questions requiring further investigations is whether local groups within a country may actually differ in their evolutionary histories, especially in recent times, and thus if analyzing such groups separately may provide additional insights into the population’s past.

In addressing this question we make use of high-coverage whole genome sequences from more than 2300 Estonian Biobank donors generated as a part of a study by Kals et al.^10^. Previous studies^2,11,12^ have shown using a smaller sample that the Estonian population is genetically structured despite the small area it occupies and absence of significant physical barriers. Here by exploiting a bigger dataset we study the fine-scale genetic structure in Estonia and assess the local differences in recent demographic history and action of natural selection between genetically defined Estonian subgroups.

**IBD segments-based clustering is informative about fine genetic structure in Estonia**

To get a first glance at the Estonian population structure we performed principal component analysis (PCA) both using only the Estonian samples (figure 1a) and by projecting Estonian samples onto PC space defined by samples representing various European populations (figure 1b). The PCA shows the presence of a genetic gradient within Estonia with the main differentiation observed between South-East and North-East of the country in agreement with previous studies^2,11,12^. This differentiation reflects a broader-scale South-North gradient in Eastern Europe (figure 1b) with Estonians from the North-East being closer to Finns while South-East Estonians projected closer to Latvians and Lithuanians.

Next, to zoom-in into the fine-scale structure in Estonia we used a subset of 468 individuals sampled in rural areas at the age of 50 or more, as this cohort is expected to be the least affected by recent migrations. We refer to this subset as “R50+” throughout the text (Methods). We used total genetic length of shared IBD segments detected with *IBDseq*^13^ as input for the fineSTRUCTURE^14^ clustering algorithm (Methods) to group the samples into genetic clusters (figure 2, supplementary text 2.3). Such an approach as opposed to the classical fineSTRUCTURE based on CHROMOPAINTER chunk count matrix was motivated by the following two ideas. First, IBD segments are expected to be on average longer and younger and thus have a more localized geographic distribution. This, combined with using total length instead of count and so giving more weight to the longer segments (see a similar approach being applied by Bycroft et al., 2019^3^) allows to focus on a rather recent genetic signal when performing the clustering. See supplementary text 2. Second, as one of the main goals of the clustering was to test for the differences in recent effective population size dynamics as infered using IBDNe^15^ clustering based on IBD-sharing patterns is a natural choice.

IBD-based analysis (figure 2) reinforces previous observations^2,11,12^ and our PCA results, namely the strong differentiation between South-East and the rest of Estonia, and provides a deeper insight into Estonian genetic structure, showing that most of the revealed clusters are highly geographically localized. The sharing matrix provides additional details. First, off-diagonal sharing also reflects geography with clusters from the same area tending to have higher inter-cluster sharing. Second, intra-cluster sharing substantially varies among clusters, implying differences in effective population size (Ne), which is also supported by the results of homozygosity-by-descent analysis (figure S2.7).

**Genetic differences between different Estonian regions are driven by isolation within the country and admixture with neighbouring groups**

In order to understand how gene flow barriers and/or differences in local population density shaped the IBD-sharing pattern in the R50+ dataset, we inferred migration surfaces using MAPS^16^. We used two windows of IBD segments length (in centimorgans, cM), 2-6 cM and more than 6 cM, which under a simplistic model of infinite population size have mean segment ages of 50 and 12.5 generations respectively^16^. Results for the two length bins generally agree with each other, suggesting higher levels of gene flow in the North along with a barrier separating South-East Estonia (supplementary text 2.4). A second barrier, separating the islands, especially Hiiumaa, from the mainland is also evident. This observation suggests that the population ancestral to modern South-East Estonians was partially isolated from the rest of the country at least since 50 generations ago. Interestingly, this genetic differentiation is consistent with linguistic data suggesting that the deepest split within the Finnic languages separates Southern Estonian from the other branches of the phylum that includes Northern Estonian^17^.

As local differences in admixture with external populations may have played a role in creating the observed genetic structure within Estonia we looked at patterns of haplotype sharing between R50+ Estonians and different non-Estonian populations (table S3.1). Here we used a conventional CHROMOPAINTER/fineSTRUCTURE/GLOBETROTTER (CP/FS/GT) approach^18^ (Methods). Figure 3 shows the results of non-negative least squares (NNLS)^1^, modelling each individual from the R50+ dataset as a result of admixture between non-Estonian groups revealed by CP/FS (Figure 3, supplementary text 3.1 and table S3.4).

Admixture signals in figure 3 show clear geographic patterns that match known historical evidence of external migration to Estonia, including Swedish settlements on the western coast and islands in 14-15th centuries and Finnish immigration to North-East Estonia in the 17th century^19^. In the latter case the genetic gradient in Estonia is consistent with the broader European trend (figure 1b) and thus higher affinity of North-East Estonians to Finns is likely to have a more complex origin. Comparing NNLS results between clusters from figure 2 we found that some of them, such as NE_1 and NE_2, stand out in terms of sharing with external groups but most of the clusters have overlapping distributions of NNLS scores (supplementary text 3.1). A similar pattern is observed in IBD-sharing (supplementary text 3.2). These results suggest that admixture with non-Estonian groups can only partially explain the fine genetic structure observed in figure 2.

**Taking fine-scale genetic structure into account sheds light on regional differences in recent effective population size dynamics in Estonia**

We show that, despite the small territory it occupies, the Estonian population is structured (figures 1 and 2, tables S2.3 and S2.4). Next, we sought to explore whether there are any region-specific differences in effective population size dynamics and action of natural selection. We hence applied *IBDNe*, which estimates effective population size (Ne) in past generations^15^, and SDS (Singleton Density Score), a tool for detecting signatures of natural selection^20^, as both methods give insight into very recent time periods, when regional differences in population history may be anticipated. For both analyses, we used the entire dataset of 2,305 samples, for which clusters were inferred using the same approach as for the R50+ subset. This resulted in 89 and 90 clusters in two independent fineSTRUCTURE runs which were then grouped into higher order clusters based on the tree topology to increase sample size per cluster with the clustering resembling that from figure 2 (figure 4a,b).

We ran *IBDNe*^15^ on the four most distinct clusters fromfigure 4a , representing four regions of Estonia: North-West, North-East, South-West and South-East and observed rather distinct Ne trajectories (figure 4c, supplementary text 4.2). In particular, all clusters (except for eSE_5) show evidence of an effective population size decline between 10 and 20 generations ago, which is not detected when the entire dataset is analyzed (Figure 4c). Overall, these results suggest that population dynamics are region-specific and hence population-wide results may depend on the sampling scheme.

Based on MAPS results, we propose that most of the differences in Ne dynamics between Estonian subpopulations may be attributed to different patterns of gene flow and external admixture. South-West and North-West Estonia are characterized by an overall high level of gene flow (supplementary text 2.4), leading to similar Ne trajectories that deviate only during the last 20 generations (Figure 4c, supplementary text 4.2). This also brings about the idea that the strong bottleneck in South-West could contribute to the observed population structure, in particular leading to differentiation of South-West and its subgroups. On the other hand, South-East Estonia has the most distinct Ne trajectory according to Figure 4c, having a substantially lower long-term Ne compared to other regions. Together with MAPS results (supplementary text 2.4) this might suggest a recent expansion of a previously small-size eSE_5-like population. This, in turn, results in a rather recent increase in relative proportion of individuals with eSE_5-like ancestry in the entire Estonian population affecting the Ne reconstructions for the entire dataset (supplementary text 4.2).

**Effective population size estimates in humans can be related to past census population size**

Given our understanding of confounders of the observed regional Ne patterns, we exploited the fine-grained temporal resolution enabled by *IBDNe* to infer changes in actual census sizes (Nc) of the ancestors of contemporary Estonians, adapting previous theoretical work^21^ to empirical case of human populations (Methods, supplementary text 4.4). We applied equation [3] (Methods) to the Estonian-wide Ne trajectory inferred using the Est1527 subset, which excludes clusters that can be considered as outliers in terms of external admixture and/or Ne trajectory (supplementary text 4.4). We then compared the inferred Nc with available historical estimates (figure 4d) showing a remarkable match between the two with the exception of the last three generations, for which *IBDNe* estimates are extrapolated from preceding time points^15^[.](https://www.zotero.org/google-docs/?Q7f1uP) However, note that the pronounced fluctuations in Nc reported by historians between 1500 and 1700 are only very roughly approximated by the Ne-derived curve which, as expected^22^, provides only relatively long-term harmonic average of Ne. Nevertheless, we suggest that when keeping in mind all the assumptions implied by the biological notion of Ne, our approach could be used to convert Ne to human Nc at any time interval for which historical records are missing, including the ones provided by Pairwise Sequential Marcovian Coalescence analysis (PSMC)^23^, which are beyond the scope of the current paper.

**Signals of recent action of natural selection in Estonia show regional differences**

All the analyses performed so far speak for South-East Estonia showing relatively strong genetic differentiation from the rest of the country and having a partially independent demographic history. So we went on to look into signals of recent action of natural selection with a specific focus on whether treating South-East Estonia independently can reveal any additional insights. In order to do so we applied singleton density score (SDS)^20^ to the entire dataset of 2,305 samples as well as to two genetically defined subsets, South-East Estonia (SE, consisting of 1,029 samples belonging to clusters eSE_1 − eSE_5 infigure 4a) and the remaining 1,276 samples from the rest of the country (nonSE) (Methods, supplementary text 5.1).

First, we inspected the genome-wide distribution of SDS p-values in the three datasets (figure 5) for any evidence of recent selection acting at individual loci. Unlike other studies that used SDS^20,24^ we don’t observe any hits with p-values below 5x10^-8^. We attribute this lack of genome-wide hits to a shorter time window within which we can detect selection in our dataset as indicated by lower average number of singletons, lower recent Ne and higher correlation between our SDS results and the difference in derived allele frequency (DAF) between Estonian and the UK dataset compared to the study by Field et al. (see supplementary text 5.3). This property of our dataset reduces our power to detect selection but it also allows us to get a sense of remarkably recent selective processes. Despite not detecting any genome-wide significant hits we observe 33 SNPs in 10 genomic loci with a p-values below 1x10^-5^ (table S5.1). Out of these loci the ones on chromosomes 4 and 9 are the most promising targets of recent population-specific selection as besides low SDS p-values they are characterized by DAF out of the range between the Finnish and the British datasets (table S5.1) and evidence of being associated with expression levels of nearby genes (table S5.2).

When we compare results for SE and nonSE we see weak correlation between standardized SDS (sSDS) values in the two subsets (figure S5.3e) with most of the SNPs having sSDS scores close to 0 which is the neutral expectation. However, there are 61 SNPs in 34 genomic regions with p-values below 1x10^-5^ in one of the two subsets but not in the other (table S5.1). Though many of those SNPs may be false-positives there are five genomic regions that might represent genuine hits specific to one of the Estonian regions as the corresponding SNPs are characterized not only by low p-values but also by F_ST_ between SE and nonSE above 0.011 which is the 99.9 percentile of the genome-wide F_ST_ distribution (table S5.1). One of these regions lies within an intron of in the GRM1 gene and includes SNPs rs75386033 and rs79907158 which have a p-value below 1x10^-8^ in SE (see supplementary text 5.4). While those SNPs are not eQTLs themselves they lie in a region which is enriched in SNPs associated with expression levels of the EPM2A gene (figure S5.4, supplementary text 5.4). This gene is associated with Lafora disease which is a form of progressive myoclonus epilepsy^25–27^. Another SNP from this list, rs7114857, lies within the GRM5 gene which has been shown previously to be a potential target of natural selection for the pigmentation phenotype^28^. See supplementary text 5.4 for details.

Next, we looked for possible signals of polygenic selection both in the entire dataset as well as in SE and nonSE by a) focusing on SNPs and testing if any GWAS category was enriched in SNPs with high absolute sSDS scores and if there was any correlation between the absolute sSDS and absolute GWAS betas (see supplementary text 5.2 for details); b) focusing on genes we used EnrichR^29,30^ to see if any functional annotation category was enriched in genes that harbour SNPs with absolute sSDS scores^31^ above 2.5 and CADD^31^ PHRED scores above 10. Using the first approach and correcting for linkage between SNPs resulted in a number of categories being enriched in high absolute sSDS scores and / or showing correlation between sSDS values and betas (supplementary text 5.3 and 5.4). Such categories largely overlap between the entire dataset and the nonSE subset and are related to lung or autoimmune diseases. However all those results lost statistical significance as FDR equal to 0.05 when removing SNPs falling into the HLA locus (tables S5.4, S5.5 and S5.6). While the results in the nonSE subset mostly replicate those obtained on the entire dataset the SE subset doesn’t show most of the signals detected in the entire dataset but shows a correlation between sSDS and betas for the “Bone mineral density category” category which has an FDR corrected p-value of 0.0505 even after removing linked SNPs and those in the HLA locus thus indicating a suggestive instance of polygenic selection specific to South-East Estonia. On the other hand, gene enrichment results show broadly the same results in all the datasets.

To conclude, here we show evidence of potential very recent and geographically localized selection providing an important case for our understanding of ongoing natural selection in humans.

**Conclusions**

Here we describe a dataset of more than 2300 high-coverage Estonian genomes making it one of the smallest populations to date with such high-resolution data available. We show that the Estonian population, despite occupying a small area with no strong geographic barriers, is genetically structured and exhibits rather pronounced interregional differences with respect to recent admixture with neighbouring groups, population dynamics and potential action of natural selection. These observations together with results of other studies suggest that population stratification may be ubiquitous in human populations, and should be taken into account in any large-scale genetic study including reconstructions of recent population history. We also show that we are able to accurately link effective population size to actual census size based on some simple assumptions about human population age structure and reproduction patterns.

Ultimately, the results of our study bring us to a fundamental question about the limits of the concept of discrete populations when studying human genetic diversity as datasets that uniformly cover broad geographic areas become common. Specifically, given the current opportunity to study very recent history including ongoing natural selection new theoretical and methodological advances might be needed to deal with spatial genetic structure directly rather than approximating it by clustering.

**METHODS**

Data reporting

No statistical methods were used to predetermine sample size. The experiments were not randomized and the investigators were not blinded to allocation during experiments and outcome assessment.

Whole Genome Sequencing data

We used whole genome sequences of 2,535 Estonian Biobank participants reported in Kals et al.^10^. Detailed information about the dataset and the way the samples were sequenced and filtered can be found in the corresponding publication while a brief description is provided in the supplementary text 1.1. Additionally to sample filtering applied by Kals et al.^10^, we removed seven samples with missing call over 3% as well as relatives up to third degree. This resulted in a dataset consisting of 2,305 individuals that was used for all downstream analyses. For all manipulations with vcf files bcftools-1.8^32^ was used unless specified otherwise while PLINK-1.9^33^ and KING-2.1.6^34^ was used to estimate relatedness.

For analyses that require phased and/or imputed data (CHROMOPAINTER, SDS) phasing and imputation was done using Eagle v2.3^35^ on the dataset consisting of 2,420 samples to benefit from the presence of related individuals and subsequently relevant samples were extracted.

All Estonian Biobank participants have signed a broad informed consent which allows research in the fields of genetic epidemiology, disease risk factors and population history. All work at Estonian Biobank is conducted according to the Estonian Human Gene Research Act. The original study generating the WGS data^10^ was approved by the Research Ethics Committee of the University of Tartu (application number 234/T-12).

The ‘Rural above 50 years old’ (R50+) panel

As information on parents’ and grandparents’ birthplace is mostly unavailable for the samples used here, we subset the 2,305 dataset for individuals born in rural areas and sampled at the age of 50 or older as we expect this cohort to be the least affected by recent migration. This resulted in a dataset of 474 individuals which we further pruned for PCA outliers (see below) and samples with more than 10,000 singletons (supplementary text 1.1-1.3). We ended up with a panel of 468 individuals, which we call “R50+”.

Non-Estonian samples

To place the Estonian population genetic variation in Eurasian context we compiled two datasets, one for PCA and one for CP/FS/GT, containing the R50+ Estonian samples each and samples from various populations predominantly representing West Eurasia. The datasets are described in tables S1.2 and S3.1. These datasets include both sequenced and genotyped samples so only overlapping positions (around 450K SNPs in both cases) are used in corresponding analyses.

Principal component analysis

We ran principal component analysis (PCA) for the entire Estonian dataset in two settings: with only the 2,305 Estonians and combining the 2,305 Estonians with 521 non-Estonian samples from 18 European populations (table S1.2). In both cases *smartPCA* from EIGENSOFT-7.2.0^37^ was used. See supplementary text 1.2 for details.

CHROMOPAINTER/fineSTRUCTURE/GLOBETROTTER

To study genetic similarities between Estonians and other European populations we used the CHROMOPAINTER/fineSTRUCTURE (CP/FS) pipeline^18^. Initial chromosome painting parameters were estimated using 30% of the phased dataset of 1068 Estonian and non-Estonian samples (table S3.1). FS was run for 15 million MCMC iterations in two parallel runs to assess convergence. The tree-building step was performed using the approach from Leslie et al.^1^ and the run with the highest observed posterior likelihood was used to cluster the samples into genetic groups. Inferred FS groups were further manually inspected and clustered into the higher-order FS populations (supplementary text 3.1). These FS groups were used as surrogate populations to infer admixture with GLOBETROTTER and estimate ancestry profile with NNLS.

Next, GLOBETROTTER (GT)^18^ was used to detect signals and dates of admixture for the Estonian groups defined using the approach described above. GT inference was performed using a “regional” approach^18,38^.

Finally, we used non-negative least-squares (NNLS)^1^ to assign relative ancestral proportions to each individual in the R50+ panel using the non-Estonian surrogate groups identified by FS as sources. NNLS values for CP/FS Estonian groups were extracted from GT output while for individual samples these were calculated with an in-house R script. Obtained results were then summarized across Estonian parishes as well as across IBD/FS clusters.

Detecting segments identical-by-descent (IBD segments)

To detect IBD segments in the Estonian dataset we applied *IBDseq* version r1206^13^ with default settings to the non-phased non-imputed dataset consisting of 2,305 Estonians. As *IBDseq* software reports only physical coordinates of a segment’s start and end we interpolated segments’ genetic length in cM using GRCh37 recombination map using R^40^. When working with the R50+ panel corresponding IBD segments were retrieved from the general output obtained on the 2,305 dataset. Homozygosity-by-descent segments were also inferred with *IBDseq*.

IBD segments between Estonians and non-Estonian individuals were detected by applying *refined IBD* version 12Jul18.a0b^39^ with default parameters except for length=1.0to the same dataset that was used for CP/FS/GT, as in this case the dataset is highly structured. This was followed by applying the *merge-ibd* utility version 12Jul18.a0b to merge together segments separated by at most 1 cM long gaps and no more than 2 positions with genotypes discordant with IBD.

Both for *IBDseq* and *refined IBD/ibd-merge* results segments shorter than 2 cM were discarded, as longer segments are detected with higher reliability.

MAPS

In order to evaluate the extent of gene flow across the whole country together with local population densities, we estimated migration surfaces using MAPS^16^, which harnesses a matrix summarizing the total number of IBD segments shared in a given population. In doing so, we used the IBD segments shared among pairs of individuals inferred with *IBDseq* as described in the previous section. Subsequently we have classified the shared genetic fragments as “short” (between 2 and 6 cM) and “long” (more than 6 cM), and performed two independent MAPS runs for each length class to assess convergence. Estonian territory was modeled as having a total of 200 demes. Each run had 5 million iterations thinned every 10,000 and preceded by a burn-in of 2 million discarded cycles. The obtained migration surfaces were subsequently plotted using the plotmaps R package^16^. We repeated the whole procedure after removing samples belonging to clusters from Figure 2 with mean sharing above 60 cM to assess their effect on MAPS results.

IBD-based fineSTRUCTURE (IBD/FS)

We used total genetic length of IBD segments longer than 2 cM as a measure of genetic similarity between pairs of individuals as described in supplementary text 2.1 and 2.2. When running fineSTRUCTURE for both R50+ and the entire dataset the first 2,000,000 MCMC iterations were removed as burn-in and subsequently MCMC was run for additional 2,000,000 MCMC iterations sampling every 10,000th run. When building the tree we used the approach described in Leslie et al., 2015^1^..

We applied this approach to the R50+ dataset (468 samples) and the entire dataset (2,305 samples). In both cases fineSTRUCTURE was run twice to assess convergence (supplementary text 2.3, tables S2.1 and S2.2). dataset to reduce the number of clusters revealed by the fineSTRUCTURE algorithm we have hierarchically joined together clusters with short terminal branches by cutting the tree at such a level so as to avoid having clusters consisting of less than 5 samples In the case of the R50+ and 50 in the case of the entire dataset.

Fst calculations

Fst between Estonian clusters was calculated using smartpca from the EIGENSOFT package v7.2.0^37^ after LD-pruning (r^2^ > 0.4, windows of 1,000 SNPs) and removing sites with MAF < 0.05 and missing rate > 0.1. Per-site Weir and Cockerham^41^ Fst estimator between SE and nonSE subsets was calculated using vcfttools^36^ after filtering sites for LD, MAF and missing rate the same way as described above.

Geographic data visualization

Geographic coordinates of the corresponding birth town/parish were assigned to each sample with birth place information available (2,168 out of 2,305 samples). For MAPS these coordinates were used directly. When plotting IBD/FS and NNLS results for the R50+ panel, coordinates of the samples were changed manually to avoid over-plotting. When plotting samples from the entire dataset jittering was used for the same purpose. Shp objects used to plot maps of Estonia with parish and county borders were retrieved from the Estonian Land Board website (Administrative and settlement units, 2018.11.01, https://geoportaal.maaamet.ee/eng/Spatial-Data/Administrative-and-Settlement-Division-p312.html). Geographic data were visualized in R^40^ with the aid of the following packages: sp^42,43^, sf^44^, rgdal^45^, rgeos^46^ and ggplot2^47^.

IBDNe

In order to reconstruct recent Ne dynamics we used *IBDNe* version 07May18.6a4^15^ with default settings. IBD segments used as input for *IBDNe* were detected with *IBDseq*^13^.

To get independent evidence of regional differences in Ne dynamics we applied *IBDNe* to samples from the People of the British Isles^1^ dataset grouped by the region of origin of individuals’ grandparents. The following regions were used: Scotland, Wales and North-East, North-West, South-East and South-West England. For the list of counties comprising these regions see table S4.1.

Genetic simulations

To simulate genetic data under various demographic scenarios to test the behavior of IBDNe we used mspms which is an ms-compatible version of msprime^48^. Commands used for simulation are provided in the supplementary text section 4.1.

Estimating actual census size based on Ne

Several lines of evidence, based both on theoretical reasoning^49^ and empirical comparisons^15^ suggest that in industrial human societies census size (Nc) is roughly 3 fold the Ne assuming a panmictic and isolated population. To obtain a more universal conversion method we adapted the approach from^22^ which incorporates inbreeding coefficient (Fis), relative fraction of males (m) and excess in variance of reproductive success compared to the Poisson distribution (DV):

$N_{b(t)}=\frac{(1+Fis)}{4}\times(\frac{1}{(1-m)\times m}+DV)\times N_{e(t)}$ [1]

In order to apply this formula to human populations we explored the possible range of the corresponding parameters to obtain the minimal and maximal values of the conversion coefficient: 0.75 (with m=0.5 and DV=−1) and 3.53 (with m=0.1 or 0.9 and DV=3) respectively (see supplementary text section 4.4)To provide a single point estimate of Nc we rewrite formula [1] as

$N_{b(t)}=1.63\times N_{e(t)}$ [2]

using a geometric mean between 0.75 and 3.5 and thus making our estimate slightly more than 2-fold away from the provided range boundaries. Note, that although there are indications that in some human populations DV can be higher than 3^51^, such cases can be considered to be at the very extreme of human reproductive behaviour spectrum as even hypothetical “super-male” populations would have a sex-average DV of 1.8 given m equals to 0.5^52^.

The value estimated using [2] corresponds to the number of individuals in reproductive age. It can be converted into total census size (Nc) of a human population at a given time point by dividing it by the estimated fraction of breeding individuals, which we here assume to be roughly 0.33 (supplementary text section 4.4). Incorporating this idea into [2] results in equation [3].

$N_{c(t)}=4.89\times N_{e(t)}$ [3]

which we used to obtain the curve in Figure 4D. Sources of historical estimates of Estonian population size used in that figure are provided in figure S4.10.

Singleton density score (SDS) selection scan

SDS^20^ analysis was applied to three datasets separately, namely, the entire dataset and it’s two subsets, Estonia SE and Estonia nonSE. The latter two were defined based on the IBD/FS results (Figure 4a): SE (individuals with South-East Estonian ancestry belonging to clusters eSE_1-eSE_5) and nonSE (individuals coming from the other parts of the country and belonging to other clusters). Data processing as well as the way SDS was run follow the guidelines of the authors of the original study^20^ and are described in supplementary text section 5.1.

Predicted functional effect of the test SNPs was assessed using Combined Annotation-Dependent Depletion tool (CADD)^31^. In addition, two alternative enrichment tests were performed to see whether candidate SNPs are enriched in a certain category of genes^29,30^ or in certain GWAS catalogue categories (<http://www.ebi.ac.uk/gwas/home>;^54^). Candidate SNPs were also checked for known e-QTL effects using the eQTLGen Consortium^55^ (http://www.eqtlgen.org/) database. Details of SNP annotation and enrichment analyses are specified in supplementary text section 5.2.

**DATA AVAILABILITY**

The sequencing data are available on demand. The procedure of applying for the access to the data can be found under the following link: <https://www.geenivaramu.ee/en/biobank.ee/data-access>.

**REFERENCES**

1 Leslie S, Winney B, Hellenthal G *et al.* The fine-scale genetic structure of the British population. *Nature* 2015; **519**: 309–314.

2 Martin AR, Karczewski KJ, Kerminen S *et al.* Haplotype Sharing Provides Insights into Fine-Scale Population History and Disease in Finland. *Am J Hum Genet* 2018; **102**: 760–775.

3 Bycroft C, Fernandez-Rozadilla C, Ruiz-Ponte C *et al.* Patterns of genetic differentiation and the footprints of historical migrations in the Iberian Peninsula. *Nat Commun* 2019; **10**: 551.

4 Raveane A, Aneli S, Montinaro F *et al.* Population structure of modern-day Italians reveals patterns of ancient and archaic ancestries in Southern Europe. *Sci Adv* 2019; **5**: eaaw3492.

5 Saint Pierre A, Giemza J, Alves I *et al.* The genetic history of France. *Eur J Hum Genet* 2020; : 1–13.

6 Berg JJ, Harpak A, Sinnott-Armstrong N *et al.* Reduced signal for polygenic adaptation of height in UK Biobank. *eLife* 2019; **8**: e39725.

7 Sohail M, Vakhrusheva OA, Sul JH *et al.* Negative selection in humans and fruit flies involves synergistic epistasis. *Science* 2017; **356**: 539–542.

8 Haworth S, Mitchell R, Corbin L *et al.* Apparent latent structure within the UK Biobank sample has implications for epidemiological analysis. *Nat Commun* 2019; **10**: 333.

9 Kerminen S, Martin AR, Koskela J *et al.* Geographic Variation and Bias in the Polygenic Scores of Complex Diseases and Traits in Finland. *Am J Hum Genet* 2019; **104**: 1169–1181.

10 Kals M, Nikopensius T, Läll K *et al.* Advantages of genotype imputation with ethnically matched reference panel for rare variant association analyses. *bioRxiv* 2019; : 579201.

11 Nelis M, Esko T, Mägi R *et al.* Genetic Structure of Europeans: A View from the North–East. *PLOS ONE* 2009; **4**: e5472.

12 Haller T, Leitsalu L, Fischer K *et al.* MixFit: Methodology for Computing Ancestry-Related Genetic Scores at the Individual Level and Its Application to the Estonian and Finnish Population Studies. *PLoS ONE* 2017; **12**. doi:10.1371/journal.pone.0170325.

13 Browning BL, Browning SR. Detecting Identity by Descent and Estimating Genotype Error Rates in Sequence Data. *Am J Hum Genet* 2013; **93**: 840–851.

14 Lawson DJ, Hellenthal G, Myers S, Falush D. Inference of Population Structure using Dense Haplotype Data. *PLoS Genet* 2012; **8**: e1002453.

15 Browning SR, Browning BL. Accurate Non-parametric Estimation of Recent Effective Population Size from Segments of Identity by Descent. *Am J Hum Genet* 2015; **97**: 404–418.

16 Al-Asadi H, Petkova D, Stephens M, Novembre J. Estimating recent migration and population-size surfaces. *PLOS Genet* 2019; **15**: e1007908.

17 Kallio P. The Diversification of Proto-Finnic. In: Ahola J, Frog, Tolley C (eds). *Fibula, Fabula, Fact: The Viking Age in Finland*. BoD - Books on Demand, 2018.

18 Hellenthal G, Busby GBJ, Band G *et al.* A genetic atlas of human admixture history. *Science* 2014; **343**: 747–751.

19 Loit A. Invandringen från Finland till Baltikum under 1600-talet. *Hist Tidskr För Finl* 1982; : 194–195.

20 Field Y, Boyle EA, Telis N *et al.* Detection of human adaptation during the past 2000 years. *Science* 2016; **354**: 760–764.

21 Laporte V, Charlesworth B. Effective Population Size and Population Subdivision in Demographically Structured Populations. *Genetics* 2002; **162**: 501–519.

22 Charlesworth B. Fundamental concepts in genetics: effective population size and patterns of molecular evolution and variation. *Nat Rev Genet* 2009; **10**: 195–205.

23 Li H, Durbin R. Inference of human population history from individual whole-genome sequences. *Nature* 2011; **475**: 493–496.

24 Okada Y, Momozawa Y, Sakaue S *et al.* Deep whole-genome sequencing reveals recent selection signatures linked to evolution and disease risk of Japanese. *Nat Commun* 2018; **9**. doi:10.1038/s41467-018-03274-0.

25 Minassian BA, Lee JR, Herbrick JA *et al.* Mutations in a gene encoding a novel protein tyrosine phosphatase cause progressive myoclonus epilepsy. *Nat Genet* 1998; **20**: 171–174.

26 Serratosa JM, Gómez-Garre P, Gallardo ME *et al.* A novel protein tyrosine phosphatase gene is mutated in progressive myoclonus epilepsy of the Lafora type (EPM2). *Hum Mol Genet* 1999; **8**: 345–352.

27 Nitschke F, Ahonen SJ, Nitschke S, Mitra S, Minassian BA. Lafora disease - from pathogenesis to treatment strategies. *Nat Rev Neurol* 2018; **14**: 606–617.

28 Palamara PF, Terhorst J, Song YS, Price AL. High-throughput inference of pairwise coalescence times identifies signals of selection and enriched disease heritability. *Nat Genet* 2018; **50**: 1311–1317.

29 Chen EY, Tan CM, Kou Y *et al.* Enrichr: interactive and collaborative HTML5 gene list enrichment analysis tool. *BMC Bioinformatics* 2013; **14**: 128.

30 Kuleshov MV, Jones MR, Rouillard AD *et al.* Enrichr: a comprehensive gene set enrichment analysis web server 2016 update. *Nucleic Acids Res* 2016; **44**: W90-97.

31 Kircher M, Witten DM, Jain P, O’Roak BJ, Cooper GM, Shendure J. A general framework for estimating the relative pathogenicity of human genetic variants. *Nat Genet* 2014; **46**: 310–315.

32 Li H, Handsaker B, Wysoker A *et al.* The Sequence Alignment/Map format and SAMtools. *Bioinforma Oxf Engl* 2009; **25**: 2078–2079.

33 Purcell S, Neale B, Todd-Brown K *et al.* PLINK: a tool set for whole-genome association and population-based linkage analyses. *Am J Hum Genet* 2007; **81**: 559–575.

34 Manichaikul A, Mychaleckyj JC, Rich SS, Daly K, Sale M, Chen W-M. Robust relationship inference in genome-wide association studies. *Bioinformatics* 2010; **26**: 2867–2873.

35 Loh P-R, Palamara PF, Price AL. Fast and accurate long-range phasing in a UK Biobank cohort. *Nat Genet* 2016; **48**: 811–816.

36 Danecek P, Auton A, Abecasis G *et al.* The variant call format and VCFtools. *Bioinforma Oxf Engl* 2011; **27**: 2156–2158.

37 Patterson N, Price AL, Reich D. Population structure and eigenanalysis. *PLoS Genet* 2006; **2**: e190.

38 Hudjashov G, Karafet TM, Lawson DJ *et al.* Complex Patterns of Admixture across the Indonesian Archipelago. *Mol Biol Evol* 2017; **34**: 2439–2452.

39 Browning BL, Browning SR. Improving the Accuracy and Efficiency of Identity-by-Descent Detection in Population Data. *Genetics* 2013; **194**: 459–471.

40 R Core Team. *R: A language and environment for statistical computing*. R Foundation for Statistical Computing: Vienna, Austria, 2018https://www.R-project.org/.

41 Weir B, Clark Cockerham C. Estimating F-Statistics for the Analysis of Population-Structure. Evolution 38: 1358-1370. *Evolution* 1984; **38**: 1358–1370.

42 Pebesma E, Bivand R. Classes and methods for spatial data in R. *R News* 2005; **5**: 9–13.

43 Bivand RS, Pebesma E, Gómez-Rubio V. *Applied Spatial Data Analysis with R*. 2nd ed. Springer-Verlag: New York, 2013https://www.springer.com/gp/book/9781461476177 (accessed 18 Jun2019).

44 Pebesma E. Simple Features for R: Standardized Support for Spatial Vector Data. *R J* 2018.https://journal.r-project.org/archive/2018/RJ-2018-009/.

45 Bivand R, Keitt T, Rowlingson B *et al.* *rgdal: Bindings for the ‘Geospatial’ Data Abstraction Library*. 2019https://CRAN.R-project.org/package=rgdal (accessed 18 Jun2019).

46 Bivand R, Rundel C, Pebesma E *et al.* *rgeos: Interface to Geometry Engine - Open Source ('GEOS’)*. 2019https://CRAN.R-project.org/package=rgeos (accessed 18 Jun2019).

47 Wickham H. *ggplot2: Elegant Graphics for Data Analysis*. Springer-Verlag: New York, 2009https://www.springer.com/gp/book/9780387981413 (accessed 18 Jun2019).

48 Kelleher J, Etheridge AM, McVean G. Efficient Coalescent Simulation and Genealogical Analysis for Large Sample Sizes. *PLOS Comput Biol* 2016; **12**: e1004842.

49 Felsenstein J. Inbreeding and Variance Effective Numbers in Populations with Overlapping Generations. *Genetics* 1971; **68**: 581–597.

50 The 1000 Genomes Project Consortium. A global reference for human genetic variation. *Nature* 2015; **526**: 68–74.

51 Austerlitz F, Heyer E. Social transmission of reproductive behavior increases frequency of inherited disorders in a young-expanding population. *Proc Natl Acad Sci U S A* 1998; **95**: 15140–15144.

52 Heyer E, Chaix R, Pavard S, Austerlitz F. Sex-specific demographic behaviours that shape human genomic variation. *Mol Ecol* 2012; **21**: 597–612.

53 Browning SR, Browning BL, Daviglus ML *et al.* Ancestry-specific recent effective population size in the Americas. *PLoS Genet* 2018; **14**: e1007385.

54 MacArthur J, Bowler E, Cerezo M *et al.* The new NHGRI-EBI Catalog of published genome-wide association studies (GWAS Catalog). *Nucleic Acids Res* 2017; **45**: D896–D901.

55 Võsa U, Claringbould A, Westra H-J *et al.* Unraveling the polygenic architecture of complex traits using blood eQTL metaanalysis. *bioRxiv* 2018; : 447367.

**Author Contribution**

### VP, LP, AM and MM designed the study. LS, TE, RM, LP and AM conducted sample management and provided access to data. MS provided historical data. VP, FM, AK, GH, FJ, RF, DM, MK, AA, DJL and LP analyzed the data. VP, FM, AK, GH, FJ, LS, RF, DM, MS, UV, MK, CT, MMo, LM, AA, DJL, TE, RM, LP, AM, MM contributed to the interpretation of results. VP, FM, AK, GH, FJ, RF, DM, MS, CT, DJL, LP, MM wrote the manuscript.

**Acknowledgements**

### This work was supported by the Estonian Research Council grants PRG243 (GH, RF, LS, LP, MM), PUT1339 (AK), PUTJD817 (MK), MOBTP108 (UV), IUT20-60 (AM, RM), IUT24-6 (AM, RM), PUT1660 (TE) and PRG184 (LM); by the European Union through the European Regional Development Fund Projects No. 2014-2020.4.01.16-0030 (FM, MM, VP), No. 2014-2020.4.01.15-0012 (AM, RM, TE, LM, GH, LS, MM), No. 2014-2020.4.01.16-0024 (DM, LP, VP), No. 2014-2020.4.01.16-0125 (RF), No. 2014-2020.4.01.16-0271 (RF) and 2014-2020.4.01.16-0125 (AM, RM, TE, LM); by NIH GIANT (AM, TE); by ERA-CVD grant Detectin-HF (AM), by NIH-BMI Grant 2R01DK075787-06A1 (TE), by the Wellcome Trust No. WT104125MA (DJL) as well as by the European Union through Horizon 2020 grant no. 810645 (MM) and PRESICE4Q (LM).

### Data analysis was performed on the High-Performance Computing Center of University of Tartu.

### The authors would like to thank the Genome Aggregation Database (gnomAD) and the groups that provided exome and genome variant data to this resource. A full list of contributing groups can be found at <https://gnomad.broadinstitute.org/about>.

### The authors would like to thank Bayazit Yunusbayev for fruitful discussion and valuable advice.

**Competing interests**

### Authors declare no competing interests.

**Figures captions**

**Figure 1. Principal components analysis of 2,305 Estonian samples.** a. Principle component analysis of the Estonian dataset. The first two PCs are shown. Individual dots are colored according to the donor’s place of birth. Estonian counties were divided into 4 groups (SE – South-East, SW – South-West, NW – North-West and NE – North-East) as shown in the map. This map was created in R (https://www.R-project.org/)^16^ using an shp object of the Administrative and settlement units provided by the Estonian Land Board, 2018.11.01 (https://geoportaal.maaamet.ee/eng/Spatial-Data/Administrative-and-Settlement-Division-p312.html). See Methods for more details. The individuals with no information available regarding their place of birth are shown in grey. b. Projecting Estonian samples onto PC space defined by European samples (Methods, Supplementary text section 1). Red crosses correspond to medians of European populations while empty circles represent individual samples. Populations are labeled as follows: Ita – Italians, Spa – Spaniards, Fre – French, Ger – Germans, Hun – Hungarians, Eng – British, Swe – Swedes, Ukr – Ukrainians, Bel – Belarusians, RuCS – Russians from Central and Southern Russia, Pol – Poles, Lit – Lithuanians, Lat – Latvians, Mor – Mordvins, RuN – Russians from Northern Russia, Estonian samples are shown in colour reflecting their position along PC1 in panel a. In both panels percentages in the axis labels show the proportion of the total variance explained by the corresponding PC.

**Figure 2. Genetic clustering of R50+ samples based on pairwise sharing of IBD segments. a**, Hierarchical relationships (tree) and the average total length of IBD segments shared between cluster members (heatmap) as inferred by fineSTRUCTURE. The length of the tree branches does not reflect any relationship between the clusters. Clusters are named to reflect their geographic distribution (E - “East”, NW - “North-West”, NE - “North-East”, SW - “South-West”, SE - “South-East”). Numbers in grey next to cluster names refer to the sample size of each cluster. **b**, Geographic distribution of inferred genetic clusters. Each symbol on the Estonian map corresponds to one individual from the R50+ subset. See section 2.3 of the supplementary text for details. This map was created in R (https://www.R-project.org/)^40^ using an shp object of the Administrative and settlement units provided by the Estonian Land Board, 2018.11.01 (https://geoportaal.maaamet.ee/eng/Spatial-Data/Administrative-and-Settlement-Division-p312.html). See Methods for more details.

**Figure 3. Relative proportions of “Baltic”, “Slavic”, Finnish and Swedish ancestry in the R50+ subset.** Modelled relative ancestral proportions of «Balts» (Latvians and Lithuanians), «Slavs» (Belarusians, Poles, Russians, Ukrainians), Finns, and Swedes attributed by applying non-negative least squares approach (NNLS) to CHROMOPAINTER/fineSTRUCTURE (CP/FS) results are shown. See supplementary text section :3.1 for details.. The colour of each parish reflects mean values of samples coming from this parish. Parishes with no samples in the R50+ dataset are filled with grey. Names in rectangles show directions to neighbouring countries. These maps were created in R (https://www.R-project.org/)^40^ using an shp object of the Administrative and settlement units provided by the Estonian Land Board, 2018.11.01 (https://geoportaal.maaamet.ee/eng/Spatial-Data/Administrative-and-Settlement-Division-p312.html). See Methods for more details.

**Figure 4. Genetic clusters of the entire Estonian dataset (2,305 samples) and their** r**ecent Ne dynamics. a**: Clustering of the entire dataset obtained the same way as in figure 2. The heatmap shows the average total length of IBD segments shared between clusters. The length of the tree branches does not reflect any relationship between the clusters. Numbers in grey next to cluster names show the number of samples in each cluster. **b**: Geography of inferred clusters. Each dot within the contour of Estonia corresponds to one individual, while waffle plots show samples for 15 major Estonian towns with each dot corresponding to 5 individuals. This map was created in R (https://www.R-project.org/)^40^ using an shp object of the Administrative and settlement units provided by the Estonian Land Board, 2018.11.01 (https://geoportaal.maaamet.ee/eng/Spatial-Data/Administrative-and-Settlement-Division-p312.html). See Methods for more details. **c**. Effective population size estimates obtained by applying *IBDNe*^15^ to the entire dataset and to 4 clusters from **a**: eNW_1, eNE, eSW_2 and eSE_5. **d**, Comparison of historical and genetic estimates of Estonian population size. Historical estimates combine census data and reconstructions based on written or archaeological sources (figure S4.6). Genetic estimates are derived from *IBDNe* results, for which Est1527 subset was used (figure S4.9) and refer to the broader population that contributed over time to the genomes of contemporary Estonians. When converting time points of the *IBDNe* curve into actual years we used the same logic as in the original publication^15^ and set generation 0 to correspond to the year when individuals in our sample had a mean age of 25 (1988). Generation time of 29 years was assumed. For year 1200 the minimum and maximum estimates are provided. In panel **c** shaded areas show 95% confidence intervals. In panel **d** shaded area corresponds to the range between the minimum and maximum genetic estimates of Nc (Methods), while the light blue line shows the geometric mean between the two. In both panels on the y axis, “k” stands for “thousands” and “M” for millions.

**Figure 5. Genome-wide plots of p-values corresponding to standardized SDS scores for the entire dataset (a) as well as SE (b) and nonSE (c) subsets.** Conditional suggestive (blue) and genome-wide (red) significance lines are drawn. Gene names are highlighted for intragenic variants with –log10(p) > 5. Datasets are described in the text and Supplementary Information SI1:5.1.
