# Supplementary material for: Differences in local population history at the finest level: the case of the Estonian population
Source: Eur J Hum Genet. 2020 Jul 25;28(11):1580–91. doi: 10.1038/s41431-020-0699-4 (PMC7575549; doi:10.1038/s41431-020-0699-4)
Supplement: Supplementary file 3 — Supplementary text [file 41431_2020_699_MOESM3_ESM.docx]

**Differences in local population history at the finest level: the case of the Estonian population**

*Vasili Pankratov^a,*^, Francesco Montinaro^a^, Alena Kushniarevich^a^, Georgi Hudjashov^a,b^, Flora Jay^c^, Lauri Saag^a^, Rodrigo Flores^a^, Davide Marnetto^a^, Marten Seppel*^d^*, Mart Kals^a^, Urmo Võsa^a^, Cristian Taccioli^e^, Märt Möls^f^, Lili Milani^a^, Anto Aasa^g^, Daniel John Lawson^h^, Tõnu Esko^a^, Reedik Mägi^a^, Luca Pagani^a,e,1^, Andres Metspalu^a,1^, Mait Metspalu^a,1^*

^a^Institute of Genomics, University of Tartu, Tartu, 51010, Estonia;

^b^Statistics and Bioinformatics Group, School of Fundamental Sciences, Massey University, Palmerston North 4474, New Zealand;

^c^Laboratoire de Recherche en Informatique, CNRS UMR 8623, Université Paris-Sud, Université Paris-Saclay, Orsay 91405, France

^d^Institute of History and Archaeology, University of Tartu, Tartu 51005, Estonia;

^e^ Department of Biology, University of Padova, Padova 35131, Italy;

^f^Institute of Mathematical Statistics, University of Tartu, Tartu 50409, Estonia;

^g^Institute of Geography University of Tartu, Tartu 51003, Estonia;

^h^Medical Research Council Integrative Epidemiology Unit, Department of Population Health Sciences, Bristol Medical School, University of Bristol, Bristol BS8 2BN, United Kingdom;

*Corresponding author vasilipankratov@gmail.com

^1^Contributed equally

**Supplementary text (for supplementary tables see the attached additional excel file)**

Table of content

[**1.** **Exploratory data analysis and dataset characteristics** 3](#_Toc43888145)

[1.1 Dataset 3](#_Toc43888146)

[1.2 Principal component analysis 3](#_Toc43888147)

[1.3 Assessing per-sample singleton count 4](#_Toc43888148)

[**2.** .**Analyses of genetic structure within Estonia** 6](#_Toc43888149)

[2.1 FineSTRUCTURE for arbitrary counting matrices 6](#_Toc43888150)

[2.2 IBD-based fineSTRUCTURE (IBD/FS) clustering on simulated data 8](#_Toc43888151)

[2.3 IBD/FS on the Estonian data 11](#_Toc43888152)

[2.4 Migration And Population-size Surfaces (MAPS) 17](#_Toc43888153)

[**3.** **Genetic consequences of interactions between Estonians and non-Estonian populations** 23](#_Toc43888154)

[3.1 CHROMOPAINTER/fineSTRUCTURE/GLOBETROTTER 23](#_Toc43888155)

[3.2 Levels of IBD segments sharing between Estonians and non-Estonian populations 30](#_Toc43888156)

[**4.** **Reconstructions of effective population size dynamics** 34](#_Toc43888157)

[4.1 Effective population size dynamics reconstruction on simulated data 34](#_Toc43888158)

[4.2 Reconstructing effective population size dynamics in Estonia 40](#_Toc43888159)

[4.3 Reconstructing effective population size dynamics in Britain 44](#_Toc43888160)

[4.4 Estimating actual census population size based on Ne 45](#_Toc43888161)

[**5.** **Singleton density score selection scan** 49](#_Toc43888162)

[5.1 Datasets 49](#_Toc43888163)

[5.2 Functional annotation of test SNPs and enrichment analyses 50](#_Toc43888164)

[5.3 Results for the entire dataset 51](#_Toc43888165)

[5.4 Regional differences in the SDS results in Estonians 58](#_Toc43888166)

[**6.** **References** 63](#_Toc43888167)

1. **Exploratory data analysis and dataset characteristics**

1.1 Dataset

The whole genome sequences used here are a subset of the Estonian Biobank which currently includes about 52,000 individuals with the majority of them being genotypes using SNP arrays. The samples to be sequenced were selected so as to represent all Estonian counties. Sequencing and data procession was done at the Broad Institute of MIT and Harvard. First we filtered individuals based on sequencing quality control filters, match between WGS and chip genotype, total number of SNVs and self-reported Estonian ethnicity as described in the original study resulting in a sample of 2,420 individuals. Next we excluded individuals based on missing genotype rate an relatives up to 3^rd^ degree reducing the number of samples to 2,305 individuals. In this dataset each Estonian county is represented by more than 50 samples except for the sparsely populated islands of Saaremaa and Hiiumaa (table S1.1).

1.2 Principal component analysis

Principal component analysis (PCA) was run using smartPCA from the EIGENSOFT [1] package in two different settings: **a)** directly performing PCA on 2,305 Estonians to detect outliers in the dataset and **b)** by projecting 2305 Estonians onto the first two PCs space defined using samples from various Eurasian populations (table S1.2) to place Estonians in the genetic context of West Eurasia. In setting **a** we directly ran PCA on the dataset filtering for MAF below 0.01, no-call above 0.03 and positions in LD (r^2^ > 0.4 within sliding windows of 200 positions). Results obtained in setting **a** were used to identify Estonian samples with extreme position in the PCA plot to be removed from the R50+ panel and from the dataset used for SDS. In setting **b** we used 255,536 bi-allelic SNPs that overlap between the different datasets and passed LD-pruning (r^2^ > 0.6 within sliding windows of 1,000 positions), MAF (<0.05) and no-call (>0.05) filters. We first calculated the principal components (PCs) based on all non-Estonian samples and then projected the Estonian individuals onto the first two PCs.

The results for setting a are shown in figures 1a and S1.1. The difference between the two is that while figure 1a shows the position of samples coming from different regions of Estonia, figure S1.1 shows the distribution of R50+ samples relative to the entire dataset and indicates the 4 samples which were removed as PCA outliers from R50+ set and from samples used for the Singleton Density Score (SDS) selection scan..


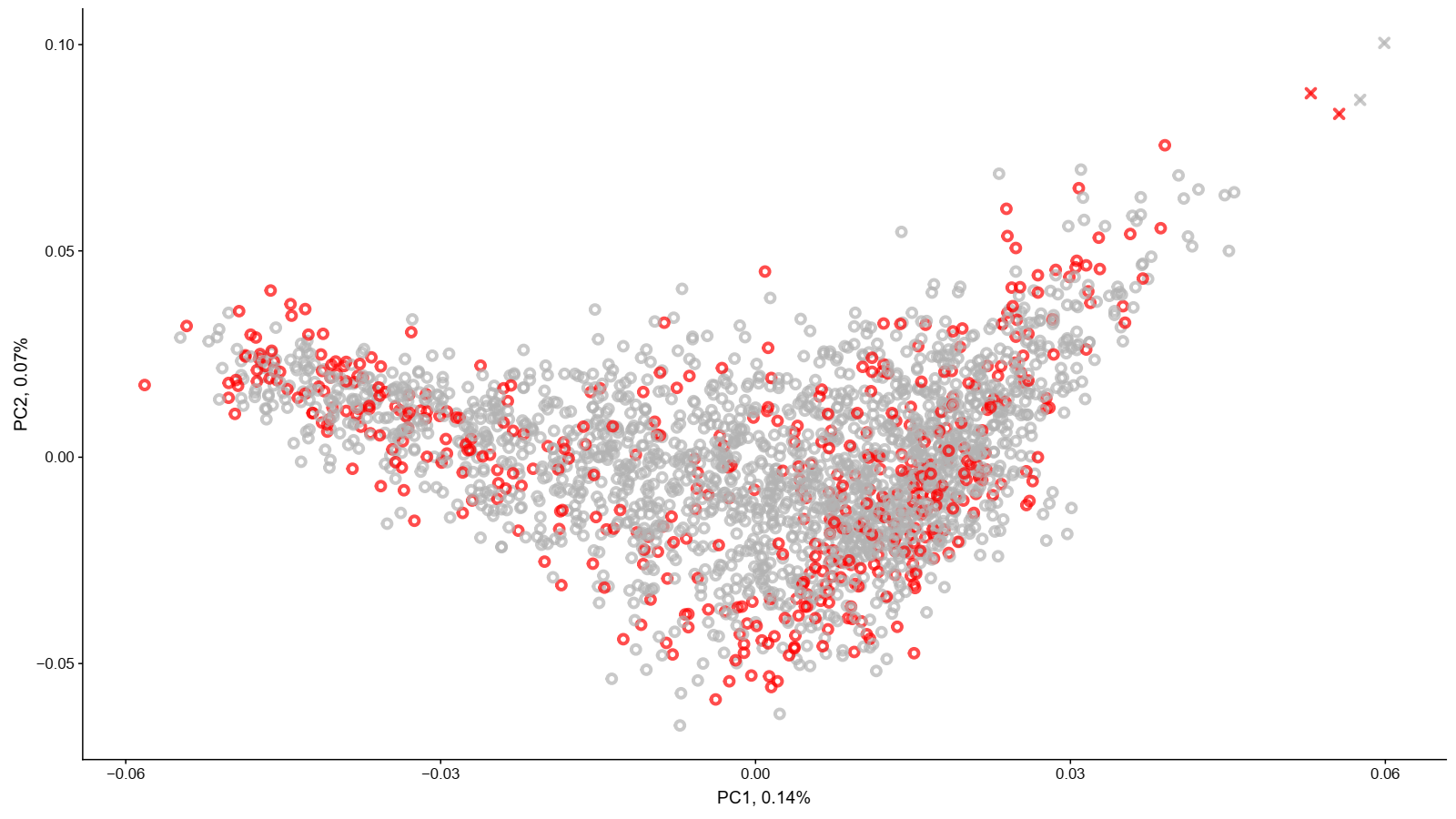


**Figure S1.1. Principal component analysis of 2305 Estonian genomes**. Results for the first two PCs are shown. Samples meeting the criteria for the R50+ panel (see Methods) are in red while the remaining samples are in grey. The samples marked as PCA outliers are depicted as crosses in the right top corner.

Figure 1a illustrates that the position of samples in the Estonians-only PCA reflects geography with the first PC being defined by the North-East and South-East regions of the country. Figure 1b shows that on a broader scale Estonians, as expected, are close to other Eastern European populations such as Latvians, Lithuanians, Finns, Russians etc. However, there is certain variability among Estonian samples in their position along PC2 with some individuals being closer to Finns, while others to Latvians and Lithuanians. A similar pattern has already been described in earlier studies [2,3,4]. Notably, there is a high concordance between PC1 in figure 1a and PC2 in 1b indicating that the North – South genetic cline in Estonia is a part of a larger-scale European cline.

1.3 Assessing per-sample singleton count

For the R50+ panel and the panels used for SDS selection scan, we removed outliers based on singleton counts. This approach is complementary to PCA as it can detect individual samples with a genetic background different from those defining the higher order PCs. Singletons were detected with vcftools 0.1.14 [5] as described in Methods. When selecting individuals for the R50+ panel a cut off of 10,000 was chosen (figure S1.2). This resulted in a removal of 4 samples from the R50+ panel. In the case of the panels used for SDS (entire dataset, SE and nonSE, see Methods) samples with singletons count between 5^th^ and the 95^th^ quantiles for the corresponding dataset were kept (figure S5.1).


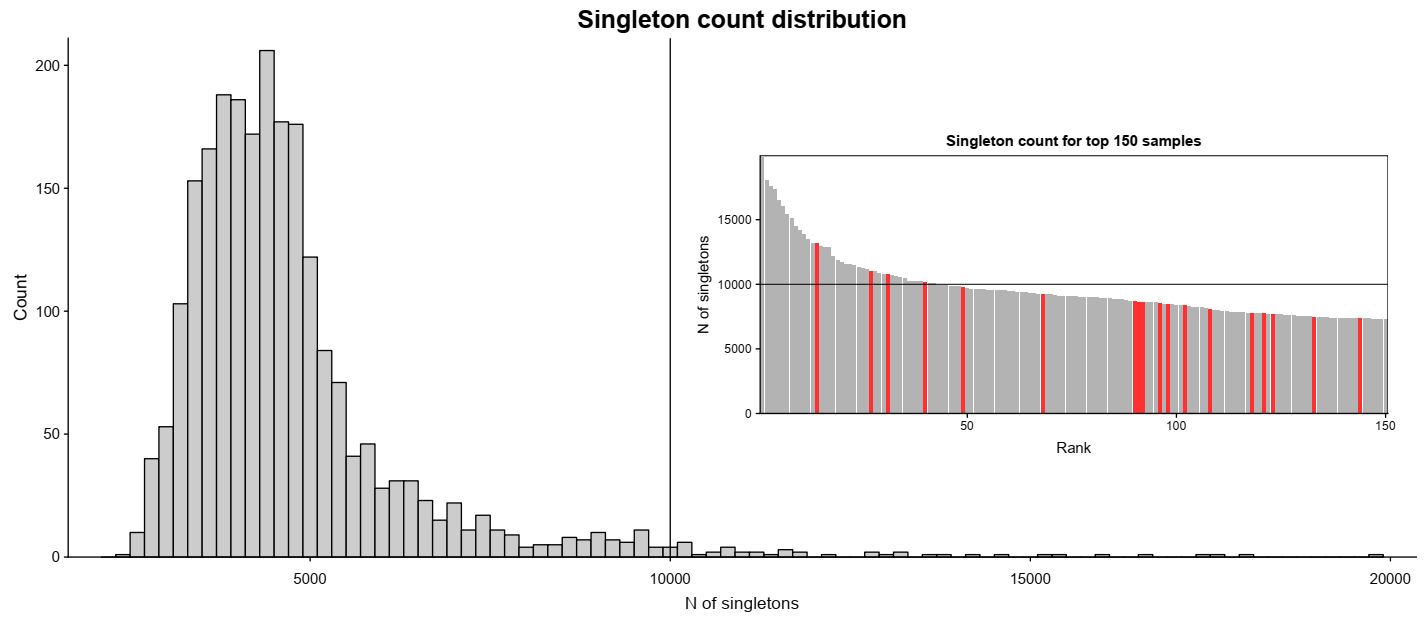


**Figure S1.2. Singleton count distribution in the entire (2,305 samples) dataset.** The inset shows the per-sample number of singletons for the top 150 samples. Red bars represent individuals falling into R50+ panel based on their age and place of birth. The chosen cut off is shown with a black line both in the main plot and in the inset. The samples removed from the R50+ panel correspond to the four red bars above the horizontal black line in the inset.

1. .**Analyses of genetic structure within Estonia**

2.1 FineSTRUCTURE for arbitrary counting matrices

In Lawson et al., 2012 [6], the number of “haplotype chunks” or “chunk counts” was modelled in general by using a hidden-Markov Model (HMM) *CHROMOPAINTER* to count these chunks in the presence of linkage disequilibrium. Every individual receives an expected number of chunks from every other individual, leading to an N by N matrix $Y_{ij}$ called the “coancestry matrix”. The *fineSTRUCTURE* model describes the sharing of chunks within a single population and across populations, leading to a K by K matrix of population sharing:

$$X_{ab}=\sum_{i\in a,j\in b} \frac{Y_{ij}}{c},$$

Where $c$ is a constant describing non-independence between chunks (detailed below). This is modelled via

$$X_{a\cdot}\sim Multinomial\left( \beta_{ab} \right),$$

Where $X_{ab}$ is simply the empirical proportion of chunks received in total by population $a$*,* summed from individuals in population $b$, accounting for $X_{ii}=0$, and $\beta_{ab}$ is learned via MCMC. The purpose of *fineSTRUCTURE* is to learn the clustering, i.e. the assignment of individuals into clusters $q_{i}\in[1,K]$ (where $K$ is learned from the data).

The asymptotic behaviour of this model was studied with respect to the central limit theorem. Specifically, the supplementary material of Lawson et al., 2012 [6] shows that when the loci are unlinked, we can derive the exact form of the corresponding Normal distribution in terms of the underlying SNP frequencies of populations. However, the central limit theorem can be expected to hold even for linked data, and therefore the likelihood will converge to a Normal distribution with parameters:

$$X_{ab}\sim Normal(\mu_{ab},\sigma_{ab}^{2})$$

Exploitation of this relationship allowed Lawson et al., 2012 [6] to rescale the likelihood in order to account for non-independence of chunks that are present in the data in practice. This was achieved through the “effective number of chunks” *c*. This leads to a simple representation for each population – a single parameter, instead of the usual two normal parameters of the mean and variance – that could be integrated out in the *fineSTRUCTURE* model. This is appropriate because the parameters are strongly related as they originate from count data.

The *fineSTRUCTURE* model is not specific to *CHROMOPAINTER* but can be applied to any matrix of counts. The primary barrier to doing so is to estimate the effective number of independent counts so that the distribution of counts within each population pair is correctly modelled.

In this paper, we propose to estimate $c$ for arbitrary count data matrices, which we then apply to the case of IBD matrices. Specifically, we use a bootstrap procedure across chromosomes in order to estimate $c$. Intuitively, this can be done since the observed number of counts between two individuals has a known mean and corresponding predicted variance, for a given genome length. Therefore, as in Lawson et al., 2012 [6] we simply scale the mean to create the correct variance.

Specifically, we consider:

$$Z_{ijr}=\frac{{\alpha Y}_{ijr}}{\sum_{j=1}^{N} Y_{ijr}}$$

Where

$$\alpha=\frac{\sum_{r=1}^{R} \sum_{i,j=1}^{N} Y_{ijr}}{R}.$$

To interpret this, note that $Y_{ijr}$ is multiplied by $\alpha$ which is $O(N)$ and divided by $\sum_{j=1}^{N} Y_{ijr}$, which is also on O(N). Therefore $Z$ is on the same scale as $Y$ but standardized in scale across the $R$ bootstrap entries.

The empirical and theoretical variance of the individual elements of $Z_{ijr}$ are then calculated and used, exactly as in Lawson et al., 2012 [6], to estimate *c* via the standard jackknife procedure. Specifically, the empirical variance is:

$$E_{ij}=\frac{\sum_{r=1}^{R} Z_{ijr}^{2}}{R-1}-\frac{\left( \sum_{r=1}^{R} Z_{ijr} \right)^{2}}{R\left( R-1 \right)},$$

And the theoretical variance is:

$$T_{ij}=\frac{N_{i}}{R}P_{ij}(1-P_{ij}),$$

Where $N_{i}=\sum_{j=1}^{N} \sum_{r=1}^{R} Z_{ijr}$, is the total count for row $i$ (hence needing to be divided by $R$ above to describe the number in each block). $P_{ij}=\sum_{r=1}^{R} Z_{ijr}/N_{i}$ is the empirical probability of each entry.

The scaling factor $c$ is then:

$$c=\frac{\sum_{i=1}^{N} \sum_{j=1,j\neq i}^{N} E_{ij}/T_{ij}}{N(N-1)}.$$

This function is available in the software FS available from [www.paintmychromosomes.com](http://www.paintmychromosomes.com) as:

fs combine -C -o <outputfileroot> <list> <of> <input> <files>

Due to the scaling procedure, the estimate of $c$ provided by this approach is appropriate when the different input matrices may vary in scale by a modest amount. If they vary too much, the empirical variance will be high and therefore $c$ will be large; this leads to a conservative estimate of the effective number of chunks. For use in human genetics, this is a favourable property.

2.2 IBD-based fineSTRUCTURE (IBD/FS) clustering on simulated data

*CHROMOPAINTER/fineSTRUCTURE* (CP/FS) [6] pipeline is currently the golden standard in population genetics when it comes to detecting population structure and assigning individuals to genetically defined groups based on patterns of haplotype sharing. However, at fine geographic scales, older haplotypes will tend to be evenly distributed in the population due to gene flow and hence carrying little information about population differentiation. One approach to gain more power to detect subtle population structure by focusing on a younger genetic signal was undertaken in a recent study by Bycroft et al., 2019 [7], where the total genetic length of CP chunks instead of their count was used as a measure of pairwise relatedness, thus giving more weight to longer and hence younger chunks. Here we undertook a similar approach but used long (> 2 centiMorgans, cM) identical-by-descent segments (IBD segments) instead of CP chunks because a) this allows focusing on an even younger signal and b) as we aimed at applying *IBDNe* [8] to the resulting groups, we would benefit from having groups as homogeneous in terms of patterns of IBD segments sharing as possible.

We validated this approach using the simulated dataset from Lawson et al., 2012 [6]. We used *refined IBD* [9] and *IBD-merge* with default settings to detect IBD segments in this simulated data and filtered the resulting output to keep only segments longer than 2 cM. Next, we summed up the genetic length (in cM) of IBD segments shared between each pair of individuals and used the resulting matrices (one matrix per each simulated “chromosome”) as input for FS. We used *fs combine -C* command to combine results from individual chromosomes simultaneously calculating *c* (see the section above). Note that a similar approach was justified in Lawson and Falush [10] with a less precise IBD measurement tool and *c* estimation procedure.

When running FS clustering the first 2,000,000 MCMC iterations were removed as burn-in and subsequently MCMC was run for additional 2,000,000 MCMC iterations sampling every 10,000th step. When building the tree we used the maximum concordance state approach (“1” value of the -T option).

When using all 200 simulated “chromosomes”, individuals were clustered into 5 groups (figure S2.1) in concordance with the simulation setting [6]. Moreover, each individual was correctly assigned to its cluster, resulting in a correlation with truth coefficient of 1, calculated the same way as in the original study [6]. Next, we used fewer “chromosomes” (10, 20, 30, 50 and 100) to investigate the effect of the amount of genetic information used (figure S2.2). We reached perfect individual assignment already using 30 “chromosomes” and obtained correlation values of 0.67 and 0.80 when 10 and 20 “chromosomes” were used correspondingly. So we show that our approach results in accurate genetic clustering. Note, however, that its’ efficiency depends on the demography of the population studied as in populations with large effective population size the amount of IBD segments may be too low for this inference to work.


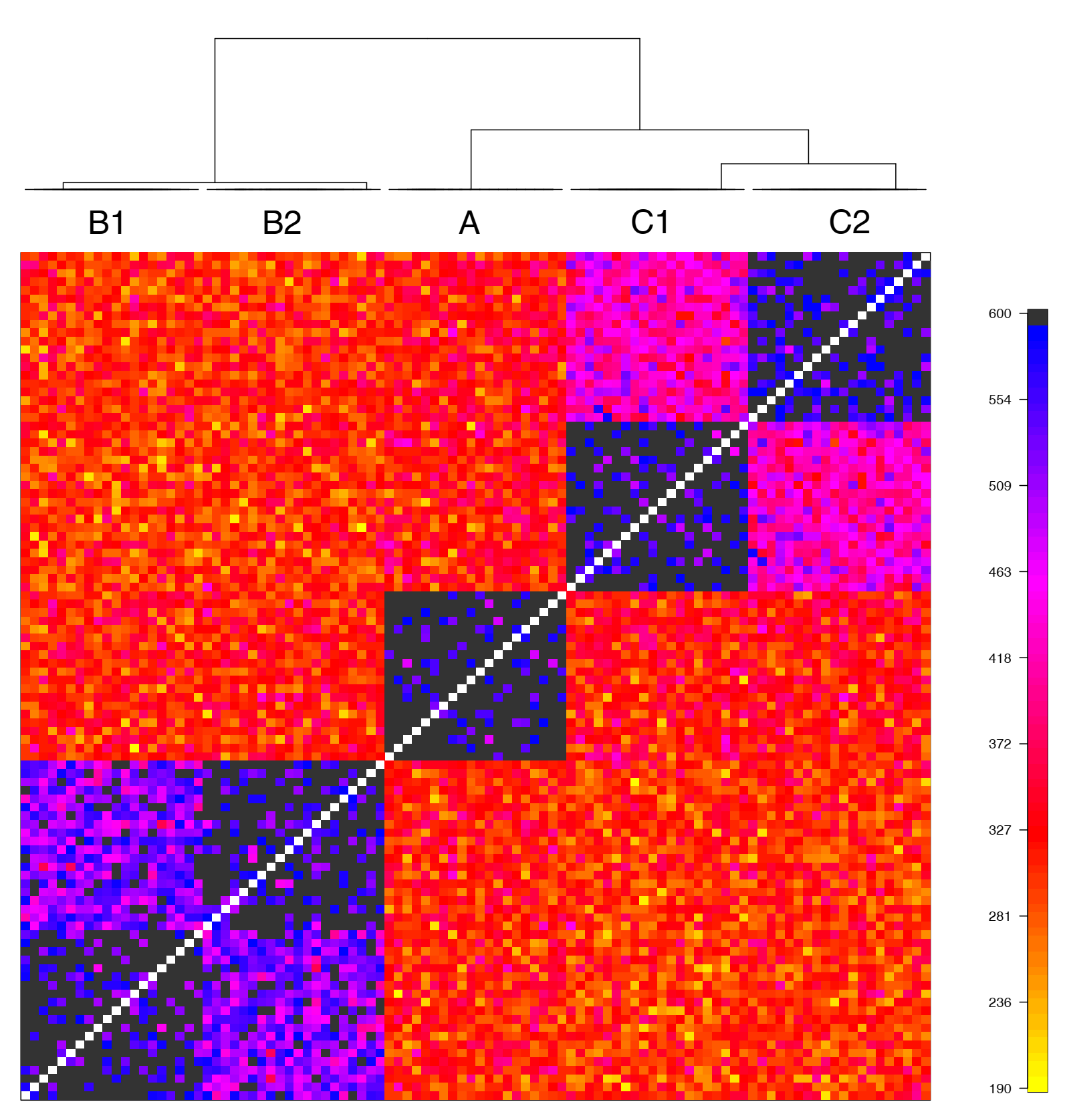


**Figure S2.1. Results of FS clustering of simulated genomes based on a pairwise total length of IBD segments.** IBD/FS was run on 200 simulated “chromosomes” each 5 Mb long. The heat map shows the total length of IBD segments in cM shared between each pair of “individuals”.


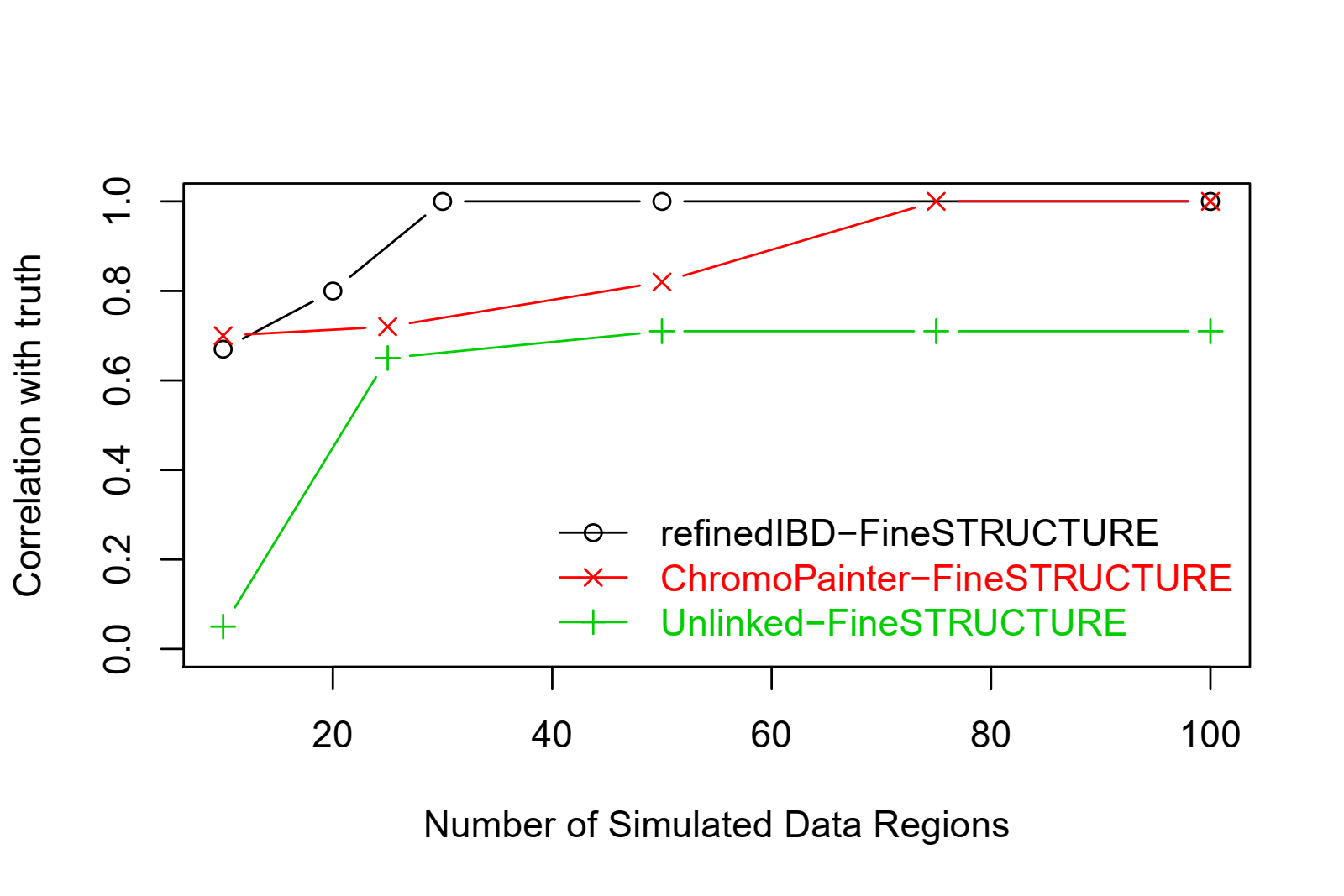


**Figure S2.2. Correlation between population structure inferred with different clustering approaches and true population assignment in the simulation setting of Lawson and Falush, 2012 [10].** Similarities between individuals were assessed using either *refined IBD*, *CHROMOPAINTER*, or Unlinked (as implemented by *CHROMOPAINTER*’s unlinked model), by summing over a number of independent simulated regions. For refined IBD total genetic length of IBD segments was used, while for CHROMOPAINTER and Unlinked we used chunk count as similarity measure. We then infer *c* as recommended for each method, using "*fs combine*” (see text). We then use *fineSTRUCTURE* to infer population structure and evaluate the correlation with truth as described in Lawson and Falush (2012) [10]. The results for *CHROMOPAINTER* and Unlinked data are those in that publication. We note that performance by those metrics in this simulation scenario might not replicate across all situations.

2.3 IBD/FS on the Estonian data

Having shown good performance of our approach we applied it to the Estonian dataset in the same way as described above. In this case IBD segments were detected with *IBDseq* [11] instead of *refined IBD.* Choosing *IBDseq* over *refined IBD* here is justified by working with samples coming from a relatively homogeneous population, which makes *IBDseq* frequency model applicable, while *IBDseq* has the advantage of not requiring phasing as well as having sequencing errors and rare alleles being explicitly accounted for. *IBDseq* was applied to the entire dataset of 2,305 samples and FS was applied to two datasets, R50+ and the entire dataset. In the former case, IBD segments shared between samples corresponding to R50+ subset were retrieved from the general output. In both cases, FS was run twice to assess convergence. This resulted in 33 and 31 clusters for R50+ and 90 and 89 for the entire dataset respectively (figures S2.3 and S2.4).


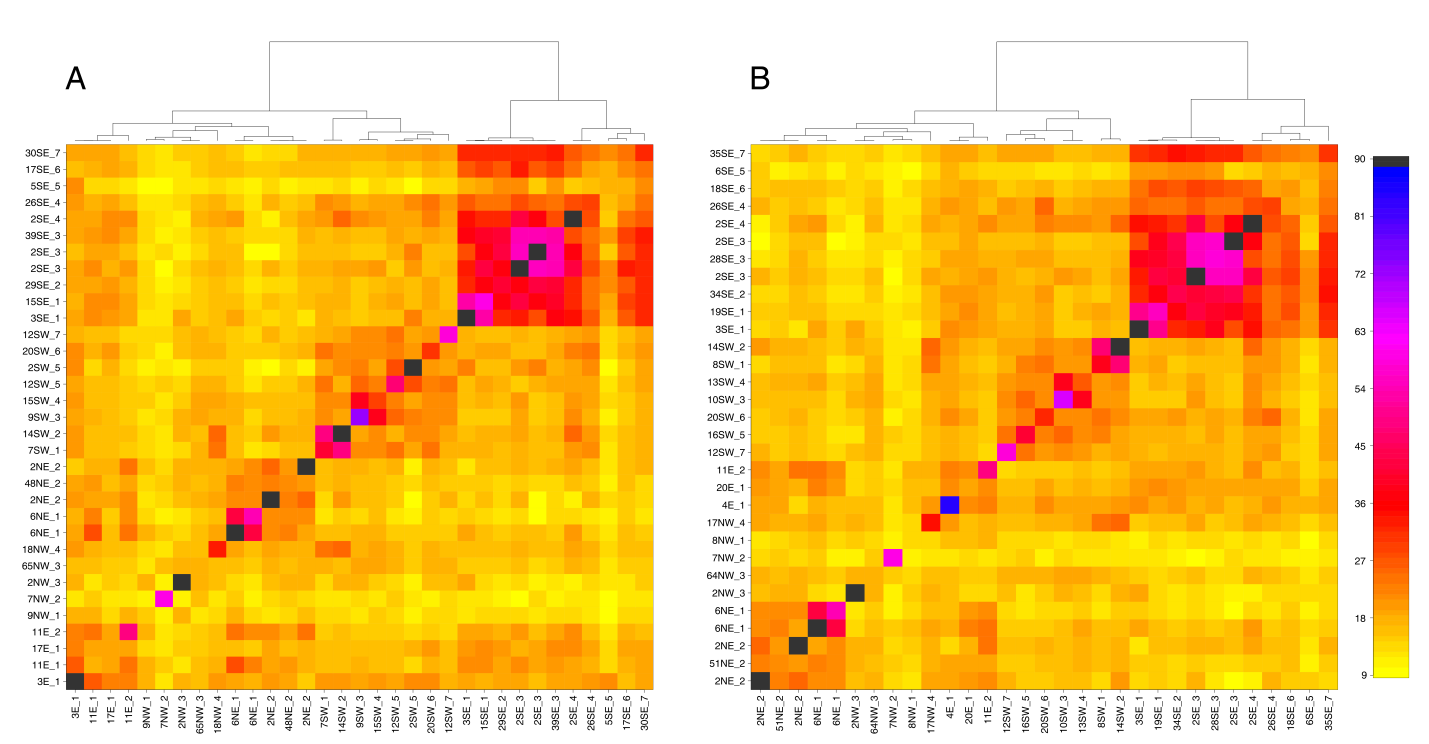


**Figure S2.3. FS clusters inferred in R50+ dataset in two independent runs (A and B).** The heat map shows the average total length in cM of IBD segments shared between members of different clusters. Segments shorter than 2 cM were discarded (see Methods). Values in the heatmap were capped at 90 cM. The first number in the rows/column names reflects the number of samples falling into the corresponding cluster, followed by a cluster name consistent with cluster names in figure 2 and S2.5 for A and B respectively. For instance, clusters 3E_1, 11E_1 and 17E_1 in the bottom left corner of panel A consist of 3, 11 and 17 samples respectively and were pulled together into cluster E_1 in figure 2.

**Figure S2.4. FS clusters inferred in the entire (2305 samples) dataset in two independent runs (A and B).** The heat map shows the average total length in cM of IBD segments shared between members of different clusters. Segments shorter than 2 cM were discarded (see Methods). Values in the matrix were capped at 90 cM. The first number in the rows/column names reflects the number of samples falling into the corresponding cluster, followed by a cluster name consistent with cluster names in figure 4a and S2.6 for A and B respectively, analogous to figure S2.3. Red horizontal lines show how the trees were cut to obtain the final clusters used in the main text. Black lines next to cluster names mark a group of samples assigned to cluster eNE in the first run but to cluster eNW_1 in the second run.

Next, to reduce the number of clusters we cut each tree and pooled together clusters below the cut line. In detail, for R50+ we cut the trees to result in clusters composed of 5 samples or more. This resulted in 22 clusters for each of the two runs. For the entire dataset, we cut the trees as shown by red lines in figure S2.4 to avoid clusters with fewer than 50 individuals, ending up with 12 clusters for each run. These are the clusters that were used in corresponding analyses. Clusters were named according to the geographical origin of the samples (figures 2, 4a and b, S2.5 and S2.6). To distinguish between clusters from the R50+ panel and the entire dataset the latter have an “e” prefix e.g. eNW_1. Concordance in cluster assignment between runs was assessed (tables S2.1 and S2.2), showing very high concordance for the R50+ dataset (430 of 468 samples were assigned to the same cluster in the two runs) and less so for the entire dataset (1777 out of 2305). In the latter case, the highest number of samples with uncertain assignment belongs to eNE/eNW_1 and eSW_1/eSW_2 clusters pairs, with clusters in each pair being geographically adjacent or even overlapping. Nevertheless, overall both runs resulted in the same geographic pattern of clusters distribution (figures 4a and b and S2.6).


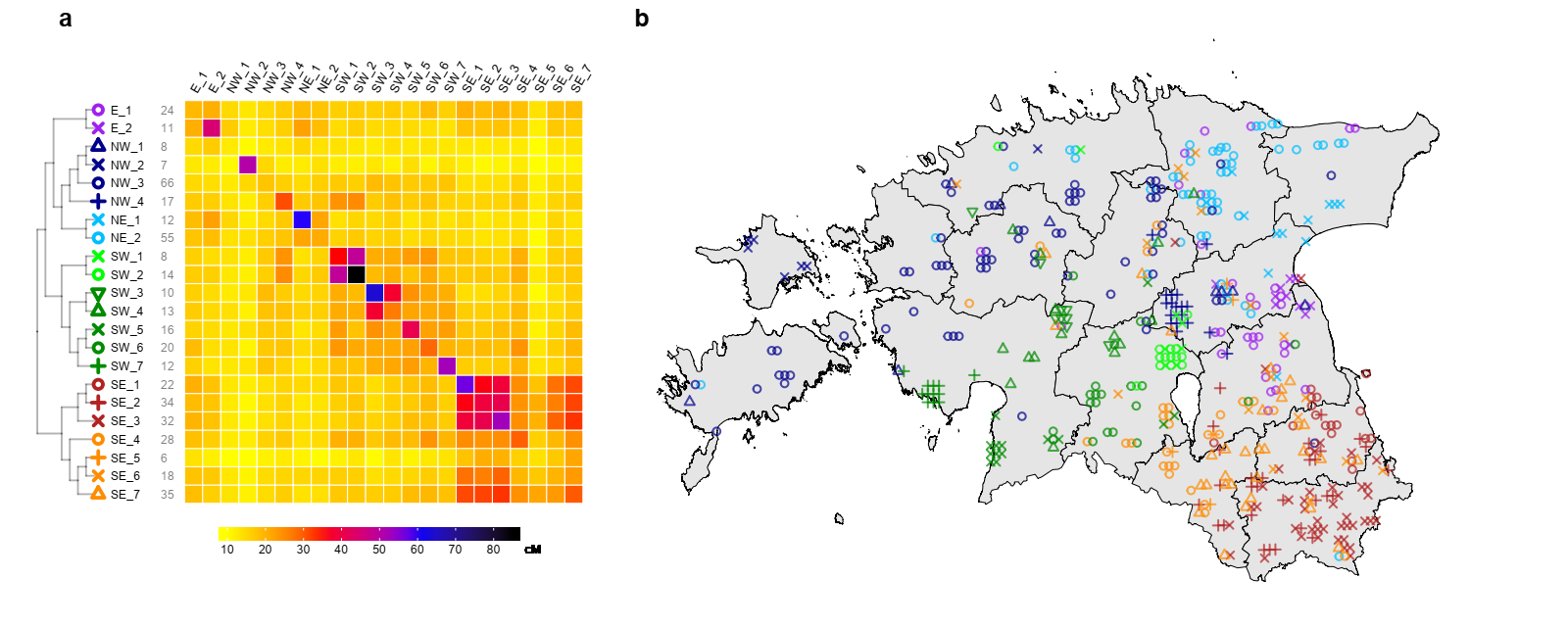


**Figure S2.5. Genetic clustering of the R50+ dataset in the second IBD/FS run. A:** Hierarchical relationships (tree) and the average total length of IBD segments shared between clusters (heatmap). Clusters obtained after cutting the tree and joining close clusters as described in the text are shown. The length of the tree branches does not reflect any relationship between the clusters. Numbers in grey next to cluster names show the number of samples in each cluster. **B:** Geographic distribution of the inferred cluster. Each dot corresponds to one individual. Corresponding results for the first run are shown in figure 2 in the main text. This map was created in R (https://www.R-project.org/) using an shp object of the Administrative and settlement units provided by the Estonian Land Board, 2018.11.01 (https://geoportaal.maaamet.ee/eng/Spatial-Data/Administrative-and-Settlement-Division-p312.html). See Methods for more details.


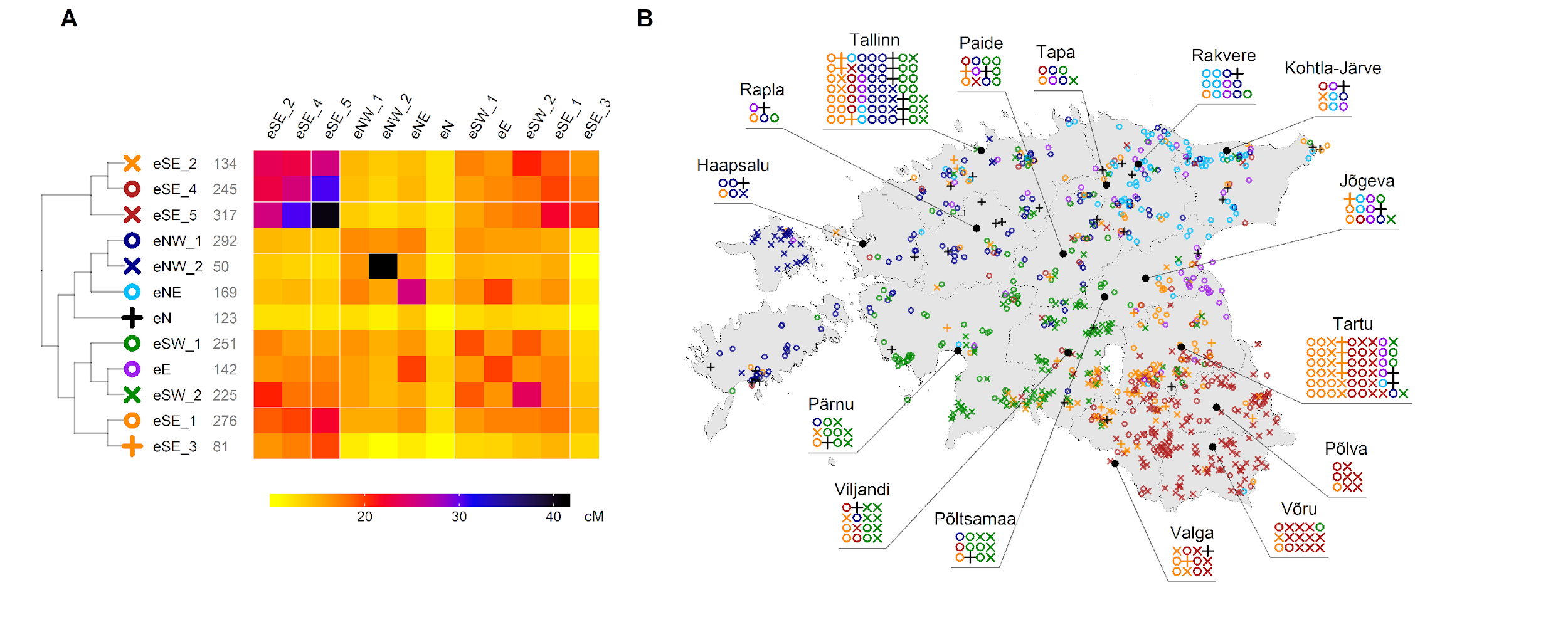


**Figure S2.6. Genetic clustering of the entire dataset (2305 samples) in the second IBD/FS run**. **A:** Hierarchical relationships (tree) and the average total length of IBD segments shared between clusters (heatmap). Clusters obtained after cutting the tree and joining close clusters as described in the text are shown. The length of the tree branches does not reflect any relationship between the clusters. Numbers in grey next to cluster names show the number of samples in each cluster. Values in the heatmap are capped to 40 cM. **B:** Geographic distribution of the inferred cluster. Each dot within the contour of Estonia corresponds to one individual, while in the waffle plots showing samples for 15 major Estonian towns each dot corresponds to 5 individuals. Corresponding results for the first run are shown in figure 4a,b. This map was created in R (https://www.R-project.org/) using an shp object of the Administrative and settlement units provided by the Estonian Land Board, 2018.11.01 (https://geoportaal.maaamet.ee/eng/Spatial-Data/Administrative-and-Settlement-Division-p312.html). See Methods for more details.

Overall the clustering results show the presence of subtle (see Fst values in tables S2.3 and S2.4) yet clear geography-driven genetic structure in Estonia. Most of the towns have profiles similar to their rural surrounding (figures 4b and S2.5) suggesting that long-distance migration from rural areas to towns was modest in most of the cases. A clear exception is Tallinn, the capital, populated by individuals with various genetic backgrounds, including South-East Estonian. However, note that Northern Estonia has a higher diversity with many individuals belonging to clusters with geographic foci in other regions of the country. This is consistent with not only Tallinn but North Estonia, in general, being an attractor for within-Estonia migrations.

Patterns of IBD-sharing presented in the heatmaps in figures 2a, 5a, S2.5 and S2.6 provide additional insight into details of population structure in Estonia. For instance, revealed clusters differ substantially in the within-cluster levels of IBD-sharing, reflecting differences in Ne which we further supported by looking at total length of homozygosity-by-descent tracks retrieved from the *IBDseq* output (figure S2.7) as well as by applying MAPS and IBDNe (See sections 2.4 and 4.2 of the Supplementary Information). Some clusters (E_1, SW_1, SW_4, SE_5, SE_6 and SE_7 in figure 2a and clusters eSE_1, eSE_3 and eSE_4 in figure 4a) have a within-cluster sharing lower than what they share with some other clusters, pointing at a potentially admixed origin of the former. This may suggest an expansion of a number of local low Ne sub-populations with subsequent admixture with other Estonians, being especially important in the case of South-East Estonia, as discussed in section 4.2.


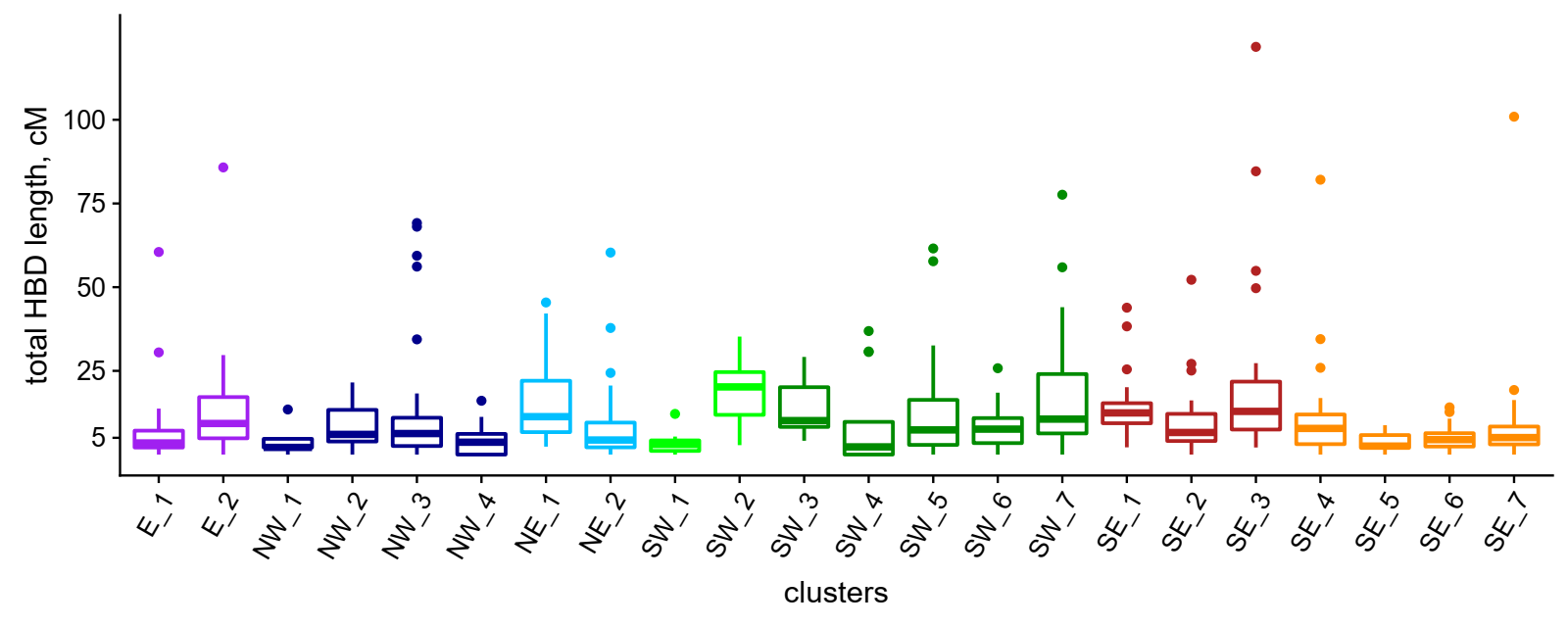


**Figure S2.7.** **Homozygosity-by-descent in the R50+ dataset.** Boxplots show distribution of per-genome total length of Homozygosity-By-Descent (HBD) tracts within clusters shown in figure 2. HBD tracts were detected using *IBDseq* [11]. The boxes show 25^th^, 50^th^ and 75^th^ quantiles, while the whiskers show values within 1.5 times the inter-quantile range (IQR) and Individual dots show outliers (values out of the range shown by the whiskers).

In order to see whether we actually gained in resolution when applying our clustering as opposed to the classical fineSTRUCTURE based on CHROMOPAINTER chunk count matrix we clustered the R50+ subset using CP/FS. Then the clusters were joined together based on their position on the tree so as to avoid clusters with less than 5 individuals, just as it was done for IBD/FS and compared the resulting clustering to the one from figure 2 (table S2.5). It can be seen that while CP/FS differentiates between South-East (clusters B, C and D) and the rest of the country (cluster A) as well as detects highly inbred clusters from the South-West (clusters E and F corresponding to SW_2 and SW_3) it fails to detect any structure in the North and South-West of the country which are potentially characterized by stronger gene flow and higher population density (see the next section). Thus indeed, at least in this particular case, our approach allowed for a finer-scale clustering resolution.

2.4 Migration And Population-size Surfaces (MAPS)

Spatial pattern of IBD segments sharing between individuals is informative about local differences in gene flow intensity and population density while focusing on IBD segments of different length enables one to get this information for different time layers. This idea is implemented in MAPS [12], which we applied to the R50+ dataset, using two length windows, 2-6 cM and more than 6 cM (figures S2.8 and S2.9). As overrepresentation of clusters with extremely high levels of IBD-sharing (figure 2) might potentially affect MAPS inference, we investigated the effect of removing the clusters with an average total length of shared IBD segments above 60 cM, which are NE_1, SW_2 and SW_3 (figures S2.10 and S2.11). Each analysis was run twice with virtually no differences between replicates (figures S2.8 – S2.11), so below we refer to two plots, figures S2.8 and S2.10. Results for the shorter segments (calculated to have an average age of 50 generations using the approach from the original study) do not differ between runs with and without clusters NE_1, SW_2 and SW_3 and reveal a relatively old (potentially more than 50 generations ago) partial isolation of South-East Estonia and the islands of Hiiumaa and Saaremaa from the rest of the country and higher population density in those islands compared to the mainland. Migration surfaces inferred from the pattern of sharing of IBD segments longer than 6 cM (figure S2.8B) generally reveal isolation of the Hiiumaa island from the mainland as well as more intensive migration along the northern coast. However for this more recent time bin we observe differences between runs that did and did not include the three clusters with high IBD-sharing, mostly affecting estimates of migration rates between Saaremaa and Northern Estonia as well as of population density in South-West Estonia (compare S2.8B to S2.10 and S2.8D to S2.10D respectively). Based on the results of our clustering (figure 2) which doesn’t distinguish between individuals from Saaremaa and North Estonia (cluster NW_3) we believe that the signal of migration between Saaremaa and North-West Estonia is genuine. As for population density in the more recent time period, both plots suggest somewhat higher density in the North compared to the South, but the difference is subtle if clusters with high IBD-sharing are not considered. Overall, our findings agree with historical evidence of Saaremaa having the biggest population density among other Estonian regions before the 1630s and active migration from there to the mainland [13], as well as with the fact that South-West Estonia was heavily affected by wars, plague and famine in the early 17th century [14, 15].


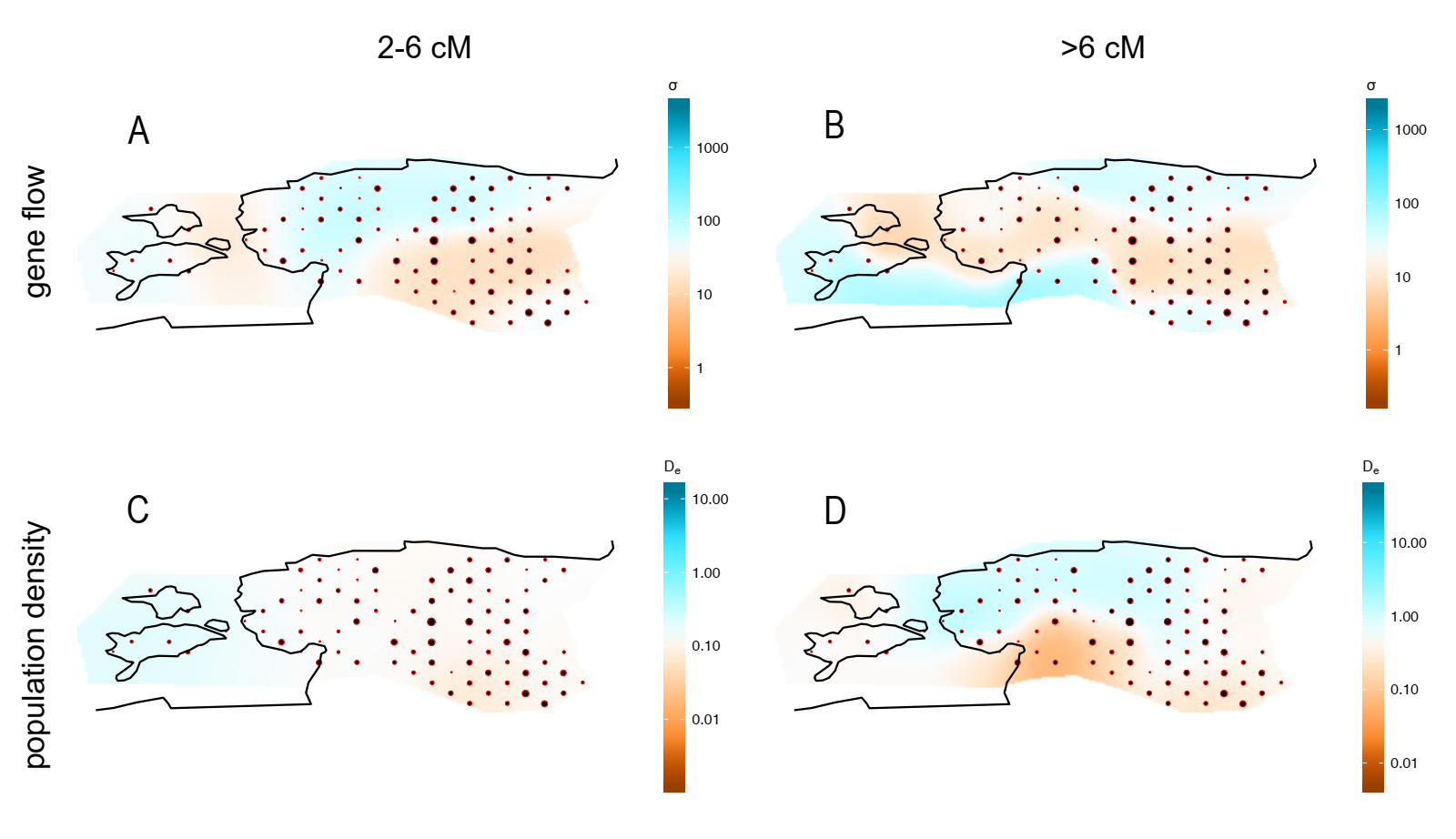


**Figure S2.8. Dispersal and population density surfaces in Estonia revealed using the R50+ dataset (run 1).** Dispersal surfaces (**A**, **B**) and population densities (**C, D**) were inferred using MAPS applied to *IBDseq* output. Panels **A** and **C** correspond to IBD segments 2-6 cM long, while panels **B** and **D** correspond to segments longer than 6 cM with an expected mean age of 50 and 12.5 generations accordingly. Samples from the R50+ dataset were used. Grid nodes’ size is proportional to the number of samples from the corresponding locations. The images including the Estonian contour were created in R (https://www.R-project.org/) using the ‘plotmaps’ package [12]. See Methods for more details.


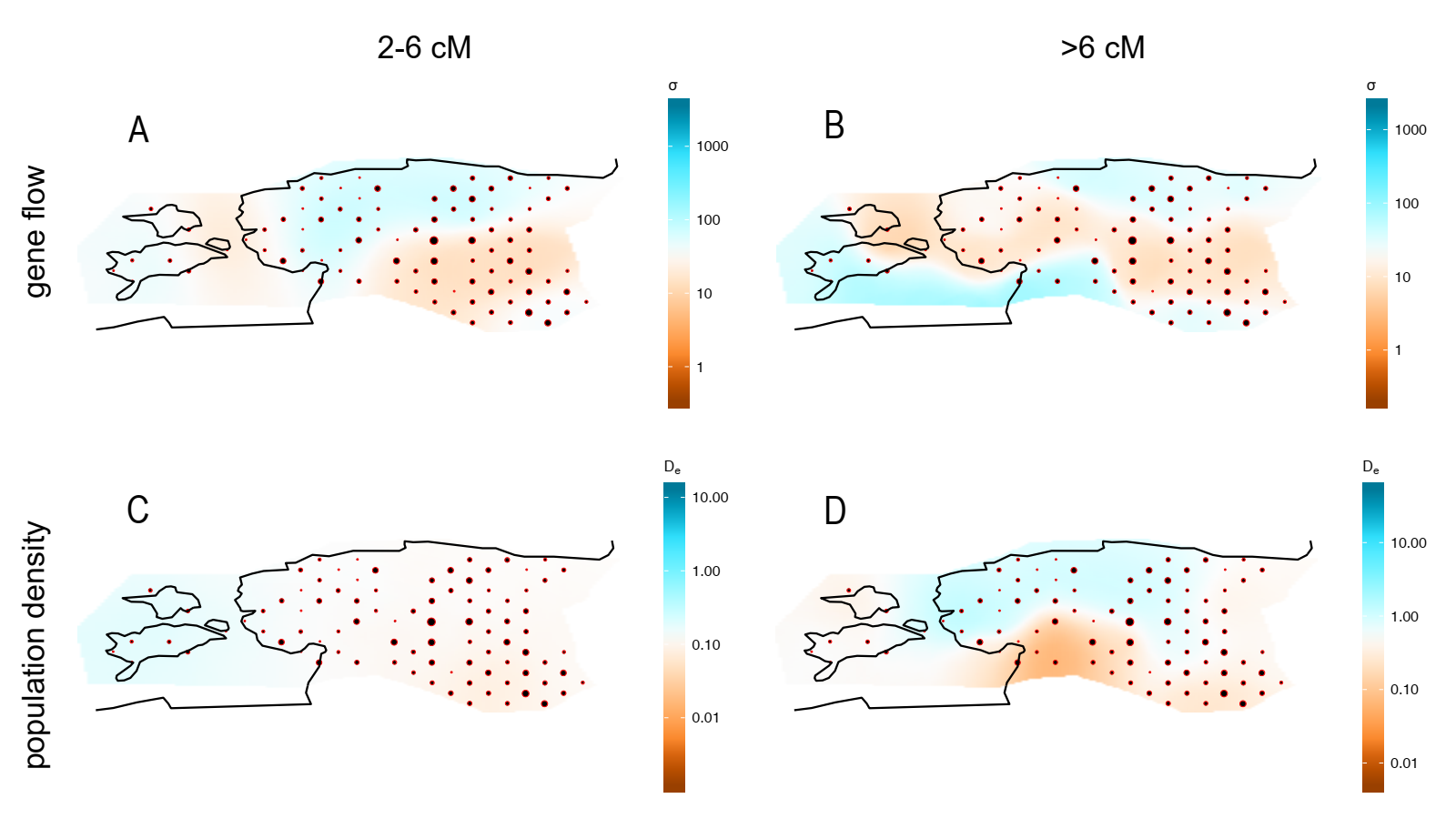


**Figure S2.9. Dispersal and population density surfaces in Estonia revealed using the R50+ dataset (run 2).** Dispersal surfaces (**A**, **B**) and population densities (**C, D**) were inferred using MAPS applied to *IBDseq* output. Panels **A** and **C** correspond to IBD segments 2-6 cM long, while panels **B** and **D** correspond to segments longer than 6 cM with an expected mean age of 50 and 12.5 generations accordingly. Samples from the R50+ dataset were used. Grid nodes’ size is proportional to the number of samples from the corresponding locations. The images including the Estonian contour were created in R (https://www.R-project.org/) using the ‘plotmaps’ package [12]. See Methods for more details.


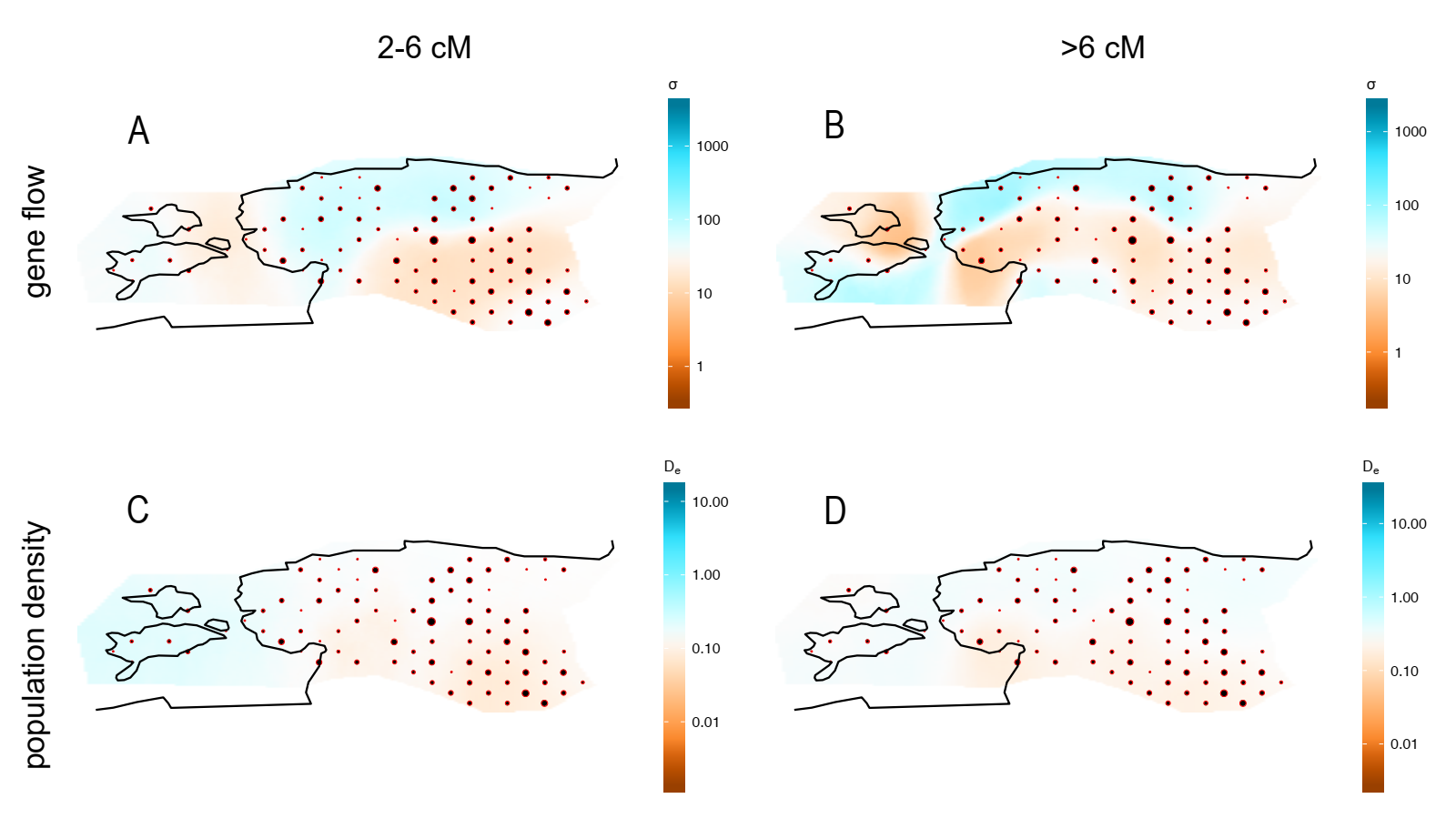


**Figure S2.10. Effect of removing clusters with high within-cluster IBD-sharing on MAPS inference (run 1).** Dispersal surfaces (**A, B**) and population densities (**C, D**) were inferred using MAPS applied to *IBDseq* output. Panels **A** and **C** correspond to IBD segments 2-6 cM long, while panels **B** and **D** correspond to segments longer than 6 cM with an expected mean age of 50 and 12.5 generations accordingly. Samples from the R50+ dataset were used, but samples belonging to clusters NE_1, SW_2 and SW_3 were removed due to high levels of within-cluster IBD-sharing. Grid nodes’ size is proportional to the number of samples from the corresponding locations. The images including the Estonian contour were created in R (https://www.R-project.org/) using the ‘plotmaps’ package [12]. See Methods for more details.


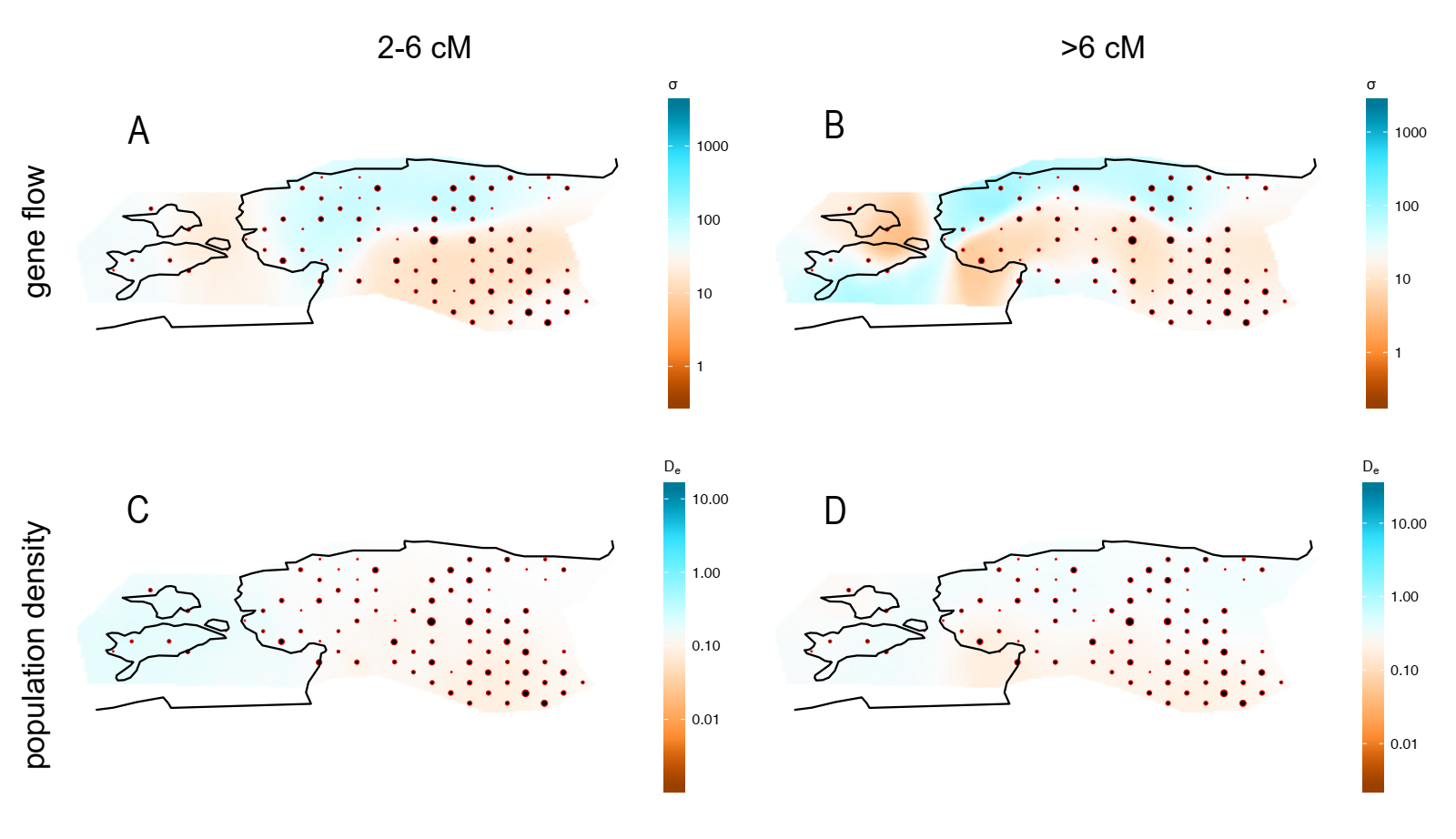


**Figure S2.11. Effect of removing clusters with high within-cluster IBD-sharing on MAPS inference (run 2).** Dispersal surfaces (**A, B**) and population densities (**C, D**) were inferred using MAPS applied to *IBDseq* output. Panels **A** and **C** correspond to IBD segments 2-6 cM long, while panels **B** and **D** correspond to segments longer than 6 cM with an expected mean age of 50 and 12.5 generations accordingly. Samples from the R50+ dataset were used, but samples belonging to clusters NE_1, SW_2 and SW_3 were removed due to high levels of within-cluster sharing. Grid nodes’ size is proportional to the number of samples from the corresponding locations. The images including the Estonian contour were created in R (https://www.R-project.org/) using the ‘plotmaps’ package [12]. See Methods for more details.

1. **Genetic consequences of interactions between Estonians and non-Estonian populations**

3.1 CHROMOPAINTER/fineSTRUCTURE/GLOBETROTTER

To study the genetic consequences of interactions between Estonians and neighbouring populations, we applied standard *CHROMOPAINTER/fineSTRUCTURE/GLOBETROTTER* pipeline (CP/FS/GT) [16]. It involves a chromosome “painting” procedure which represents each chromosome of an individual (the recipient) as a mixture of chunks received (copied) from every other individual in the dataset (donor). The number of chunks copied by a recipient from each of the donors makes a “copying vector” which are used in the FS algorithm to group individuals into populations.

Figure S3.1 depicts deep population structure within the combined dataset of Estonian R50+ panel and 600 non-Estonian samples (the list of non-Estonian samples used for this analysis can be found in table S3.1). The dendrogram was visually inspected and individual clades were manually clustered into larger FS populations based on population of origin (coloured rectangles). These FS populations included samples with identical self-identified population label, or closely related groups of samples, for example, Slavs. Estonian samples formed 13 geographically closely related groups, which were treated separately. Generally, these results are consistent with clustering shown in figure 2, illustrating strong differentiation between South-East Estonia and other regions as well as finer division into South-West, North-West and North-East. However, note, that using total IBD length in figure 2, in fact, provides additional resolution compared to standard CP/FS shown here. Also by comparing these results to the one obtained when applying CP/FS to the R50+ samples only (table S2.5) one can see that adding the non-Estonian samples increases the sensitivity and results in detecting more clusters. This is most likely a consequence of Estonian individuals from different regions of the country having different copying vectors with respect the non-Estonian populations. Three Estonian groups (labelled E1, E2 and E11 in figure S3.1) included in total ten non-Estonian samples from neighbouring populations (Finns, Latvians and Poles). In addition, one Finnish group (F1) included two samples from northeastern Estonia. These ten non-Estonian and two Estonian samples were excluded from further analysis. Other than these few samples, Estonians form a dense cluster related to other Finno-Ugric speaking groups. Manually combined FS groups were used as surrogate populations to infer admixture with *GLOBETROTTER* (GT) and estimate ancestry profile with the non-negative least squares (NNLS) approach. GT allows the structure of unsampled source populations which were involved in the admixture event(s) to be assessed by modelling them as a mixture of sampled surrogate populations. Estonian clusters were only allowed to copy from external surrogates, but not from other Estonians. CHROMOPAINTER parameters were estimated for each Estonian target group individually and the average over all target populations was used to prepare input copying vectors for GT. Two separate runs, with and without standardization by “NULL” individual, were performed and consistency between runs was checked. To assess whether unbalanced surrogate population sample size could have biased our GT inference, we performed five additional GT runs by down sample both target and surrogate populations to 20 individuals.


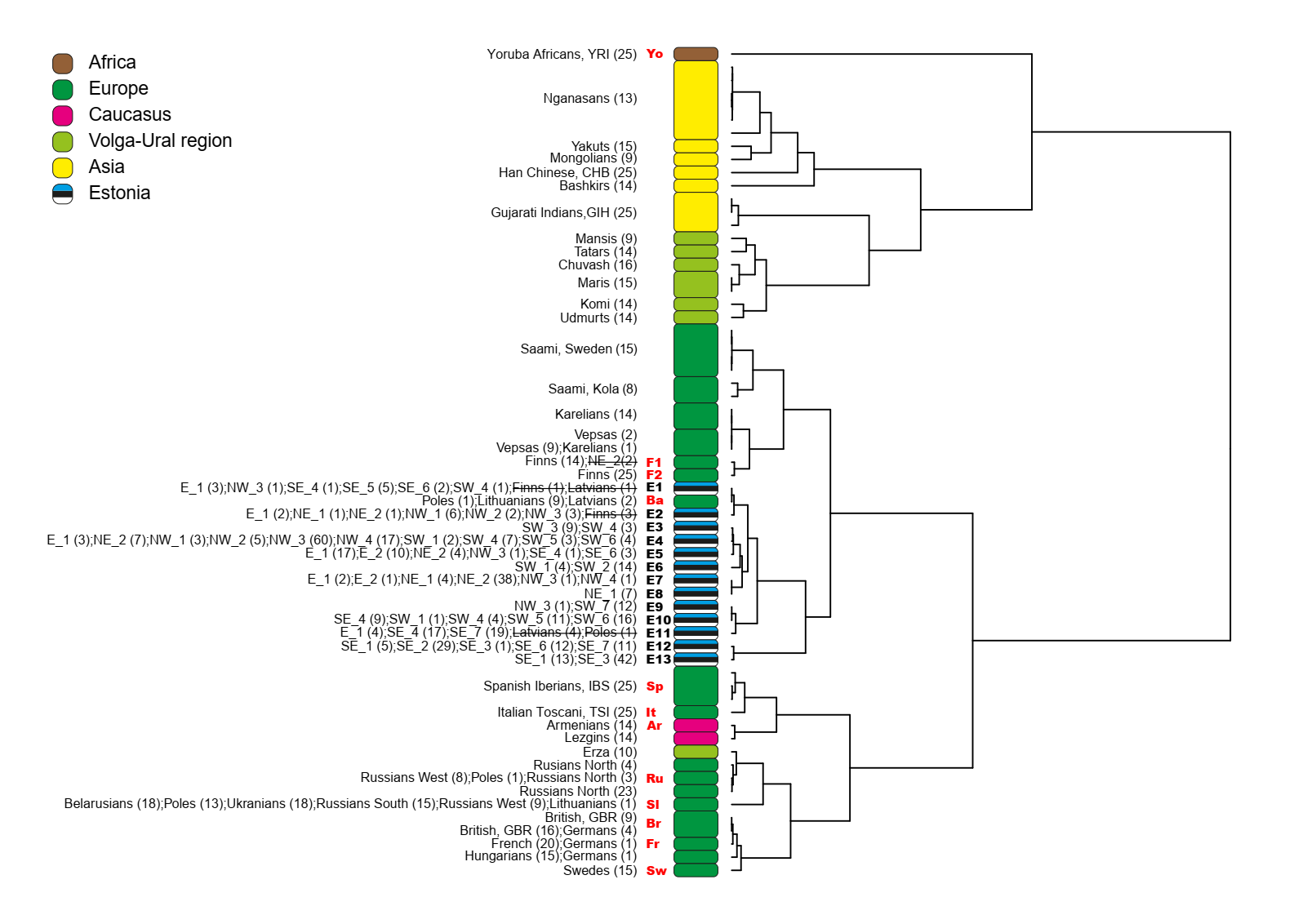


**Figure S3.1. CHROMOPAINTER/fineSTRUCTURE clustering of a joined dataset of 468 R50+ Estonian and 600 non-Estonian samples.** Estonian samples are labeled according to their cluster assignment in figure 2. The number of samples in each group is shown in brackets. Manual grouping used for GT and NNLS is shown by rectangles coloured according to geographical affiliation. Estonian clusters are labeled E1 to E13. Non-Estonian clusters revealed as potential source populations in GT (table S3.2) are marked in red and labeled according to the predominant population: Yo – Yoruba, F1 and F2 – Finns (in NNLS analysis values for these were summed together), Ba – «Balts», Sp – Spanish, It – Italians, Ar – Armenians, Ru – Russians, mainly from Kostroma, Sl – «Slavs», Br – British, Fr – French and Sw – Swedes. Estonian samples within non-Estonian clusters and vice-versa (crossed out in the figure) were excluded from corresponding clusters when running GT and NNLS.

To get deeper insights into the admixture processes driven by non-Estonian populations, “regional” GT approach was applied [16,17] – Estonian samples were only allowed to copy from non-Estonian surrogate populations, but not from other Estonian groups. All inferred admixture events were statistically significant and were classified as single date admixture between two sources. The inferred sources included Finns on one hand and Slavs or Balts on the other (table S3.2). All admixture events were dated very closely, about 18 generations (520 years) ago on average (range 13–32 generations ago) matching historical evidence of Finns’ settlement in North-Eastern Estonia [18]. However, the obtained goodness-of-fit (R^2^) estimates for a single date of admixture were poor (mean 0.59, range 0.32–0.88) likely reflecting complex historic admixture between Estonians and other European populations characterized by continuous gene flow from external sources, rather than single pulse admixture events.

We note that there is considerable variability in the sample size between clusters used as source for GT ranging from 8 to 74 individuals. For instance, the Slavic cluster (Sl in figure S3.1) is the largest among all sources tested was identified as the best-match source population of admixture in five out of 13 Estonian groups (main run, null.ind 0 in table S3.2). To assess whether our GT results could be biased by unbalanced sample sizes, we performed five additional “regional” GT runs by randomly downsampling, where possible, both surrogate and target populations to 20 individuals and repainting Estonian samples. All balanced GT runs returned results similar to the main inference and showed highly compatible inferred types of events, admixture dates, goodness-of-fit scores and inferred best-match source populations (table S3.2).

We also compared different groups revealed by CP/FS using total variation distance (TVD) (table S3.3). According to this metric, Estonian groups show high similarity to each other as well as to other populations from the region of Eastern and North-Easter Europe, namely Russians and other Slavic populations, Latvians and Lithuanians, Hungarians and Swedes. Estonian groups E7 and E8 consisting mostly of individuals from North-East Estonia show the most distinct behaviour compared to other Estonians due to higher similarity to Finns and lower similarity to Balts and Slavs.

Next, to quantify the genetic impact that contacts with different external populations had on the Estonian gene pool we applied NNLS [19] first to Estonian clusters revealed by CP/FS (table S3.4) and then to individual samples from the R50+ panel (figures 3 and S3.2 – S3.5). Results obtained for Estonian clusters agree with GT showing that the main source of admixture are populations represented by clusters Ba (Balts), Sl (Slavs) and F1 (Finns), and to a lesser extent – F2 (a second group of Finns) and Sw (Swedes) (table S3.4), accounting together for more than 90% of the NNLS signal. Note that NNLS simply represents each individual as a combination of provided non-Estonian sources. Therefore, these results only show relative genetic input from tested populations, but should not be interpreted as an indication for a lack of private Estonian ancestry. Applying NNLS to individual samples enabled us to assess the distribution of admixture signals both among IBD/FS clusters from figure 2 and among geographic regions of Estonia (figures 3 and S3.2 – S2.5). In these figures results for the most informative source groups are provided and NNLS scores for F1 and F2 are summed together as there two clusters likely represent the same source population. The geographic distribution of NNLS scores matches geography and the history of interactions between Estonians and tested population: the Baltic signal is strongest in the South along the Latvian border (figure S3.2); the Finnish signal is focused in North-East Estonia (figure S3.3) and Swedish - in the North-West, especially the islands (figure S3.5) in perfect agreement with historical records of Finnish and Swedish migrations [18]. On the other hand, the signal of admixture with Slavs doesn’t display any strong geographic pattern but exhibits a high variance between individuals (figure S3.4). This could result from unlocalized recent admixture, which is expected to happen between Estonians and Slavs, mostly represented by Russians, during the course of the 20th century.


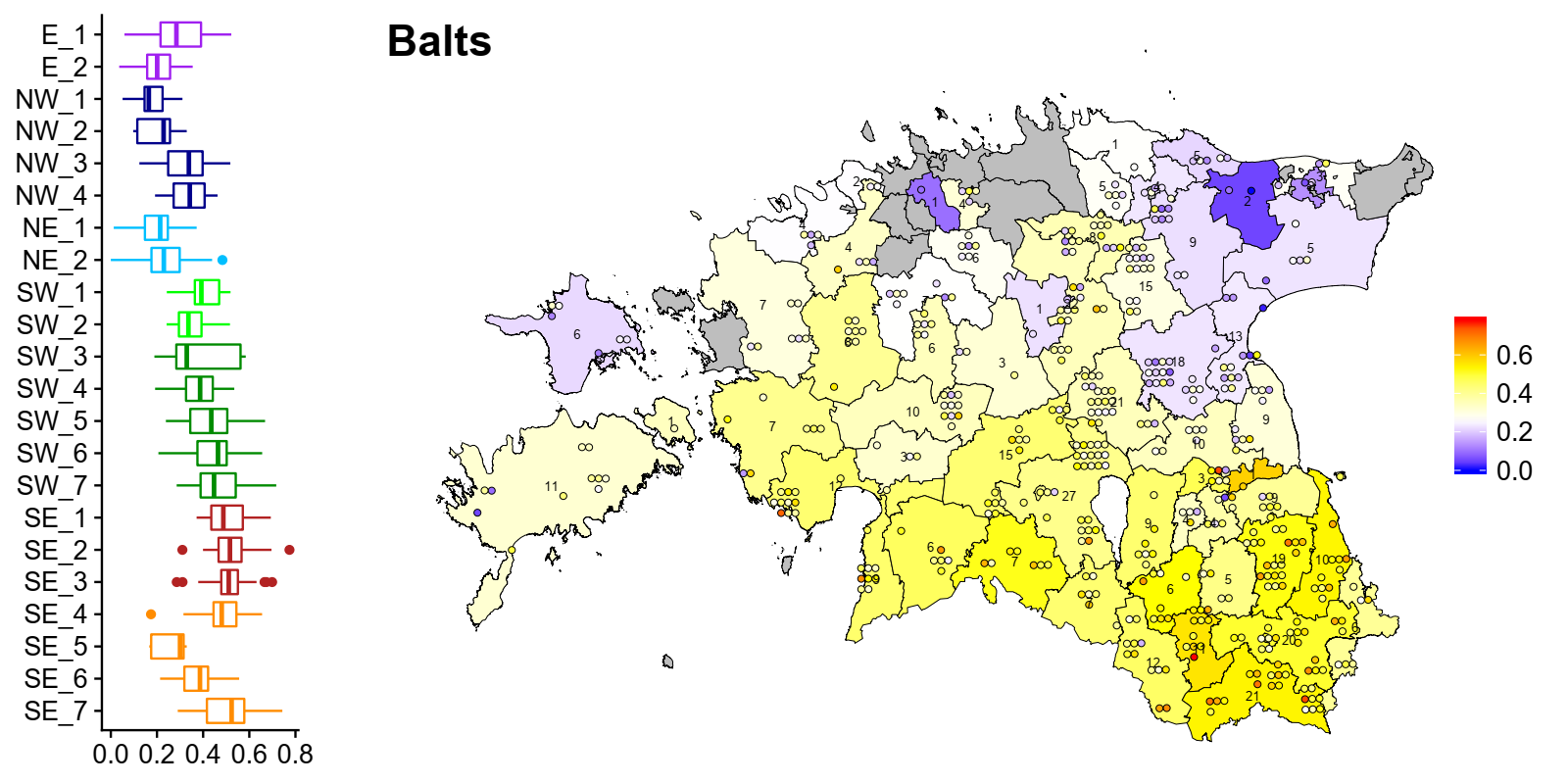


**Figure S3.2. Relative proportion of “Baltic” ancestry in the R50+ subset.** NNLS values for «Balts» (“Ba” cluster in figure S3.1) as surrogate source are shown. Two Estonians samples clustering together with Finns (figure S3.1) were excluded. NNLS values of individual samples are shown with coloured dots on the map. The fill of each parish reflects mean value for samples coming from the corresponding parish. The number of samples from each parish is shown on the map. Parishes with no samples in the R50+ dataset are filled with grey. On the left boxplots showing NNLS values for the clusters from figure 2 are presented. The boxes show 25^th^, 50^th^ and 75^th^ quantiles, while the whiskers show values within 1.5 times the inter-quantile range (IQR) of 25^th^ and 75^th^ quantiles. Individual dots show outliers (values out of the range shown with whiskers). Clusters are labeled as in figure 2 referring to their geographic localization: E – East, NW – North-West, NE – North-East, SW – South-West, SE – South-East.


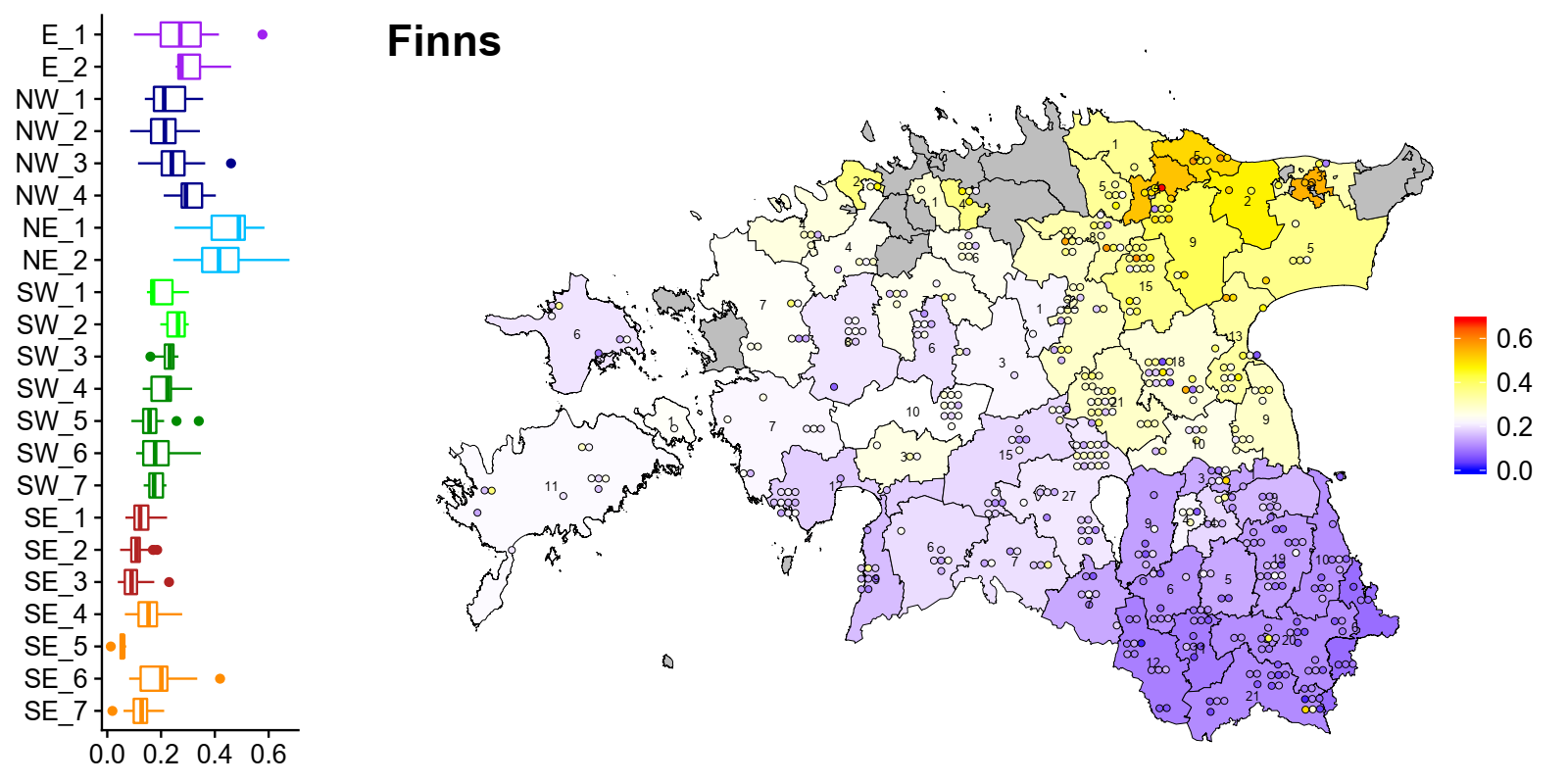


**Figure S3.3. Relative proportion of Finnish ancestry in the R50+ subset.** NNLS values for Finns (clusters “F1” and “F2” in figure S3.1, values for the two are summed together) as surrogate source are shown. Two Estonian samples clustering together with Finns (figure S3.1) are excluded. NNLS values of individual samples are shown with coloured dots on the map. The fill of each parish reflects mean values of samples coming from the corresponding parish. The number of samples from each parish is shown on the map. Parishes with no samples in the R50+ dataset are filled with grey. On the left boxplots showing NNLS values for the clusters from figure 2 are presented. The boxes show 25^th^, 50^th^ and 75^th^ quantiles, while the whiskers show values within 1.5 times the inter-quantile range (IQR) of 25^th^ and 75^th^ quantiles. Individual dots show outliers (values out of the range shown with whiskers). Clusters are labeled as in figure 2 referring to their geographic localization: E – East, NW – North-West, NE – North-East, SW – South-West, SE – South-East.


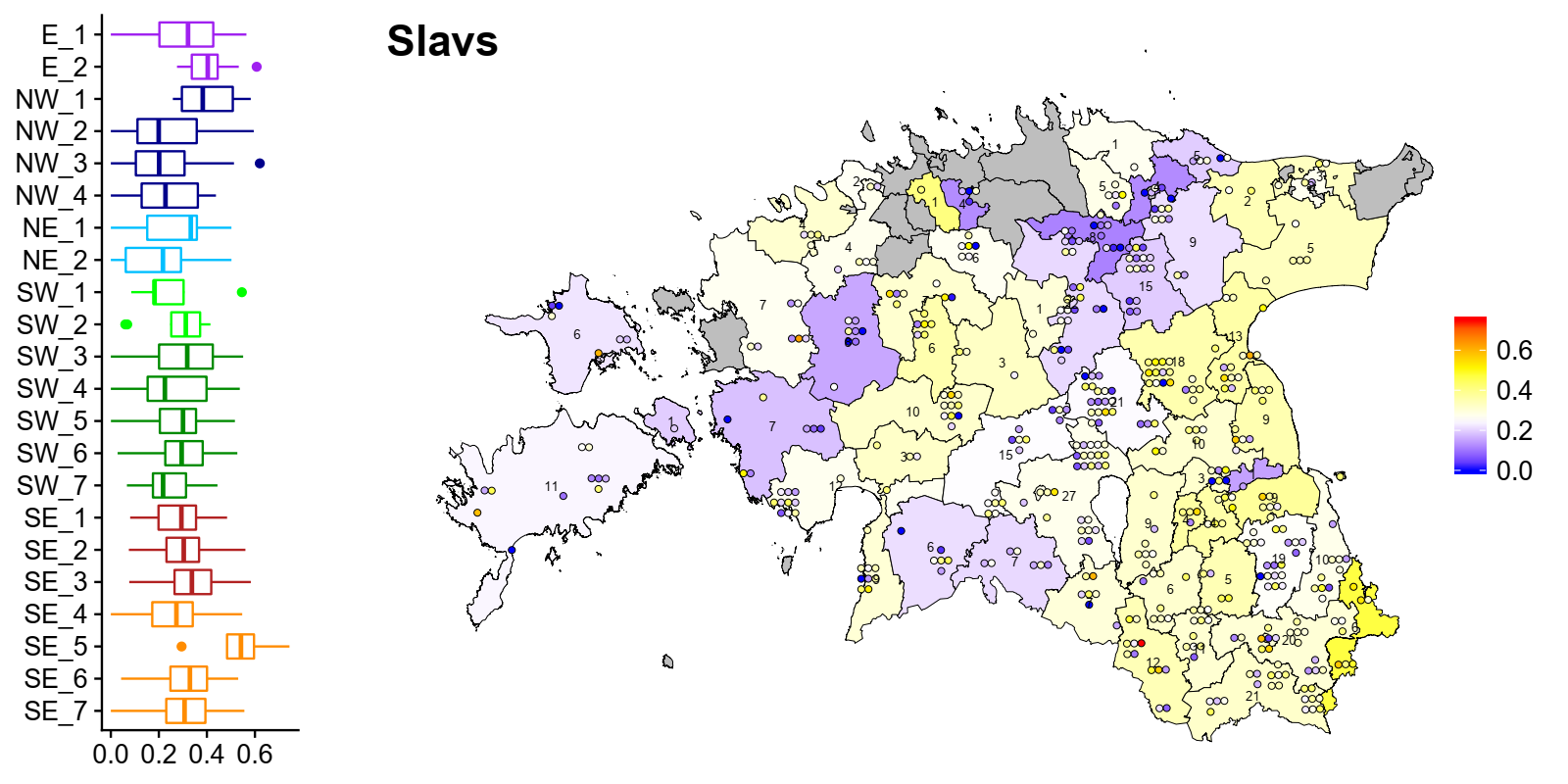


**Figure S3.4. Relative proportion of “Slavic” ancestry in the R50+ subset.** NNLS values for «Slavs» (cluster “Sl” in figure S3.1) as surrogate source are shown. Two Estonians samples clustered together with Finns (figure S3.1) and were not used here. NNLS values of individual samples are shown with coloured dots on the map. The fill of each parish reflects mean values of samples coming from the corresponding parish. The number of samples from each parish is shown on the map. Parishes with no samples in the R50+ dataset are filled with grey. On the left boxplots showing NNLS values for the clusters from figure 2 are presented. The boxes show 25^th^, 50^th^ and 75^th^ quantiles, while the whiskers show values within 1.5 times the inter-quantile range (IQR) of 25^th^ and 75^th^ quantiles. Individual dots show outliers (values out of the range shown with whiskers). Clusters are labeled as in figure 2 referring to their geographic localization: E – East, NW – North-West, NE – North-East, SW – South-West, SE – South-East.


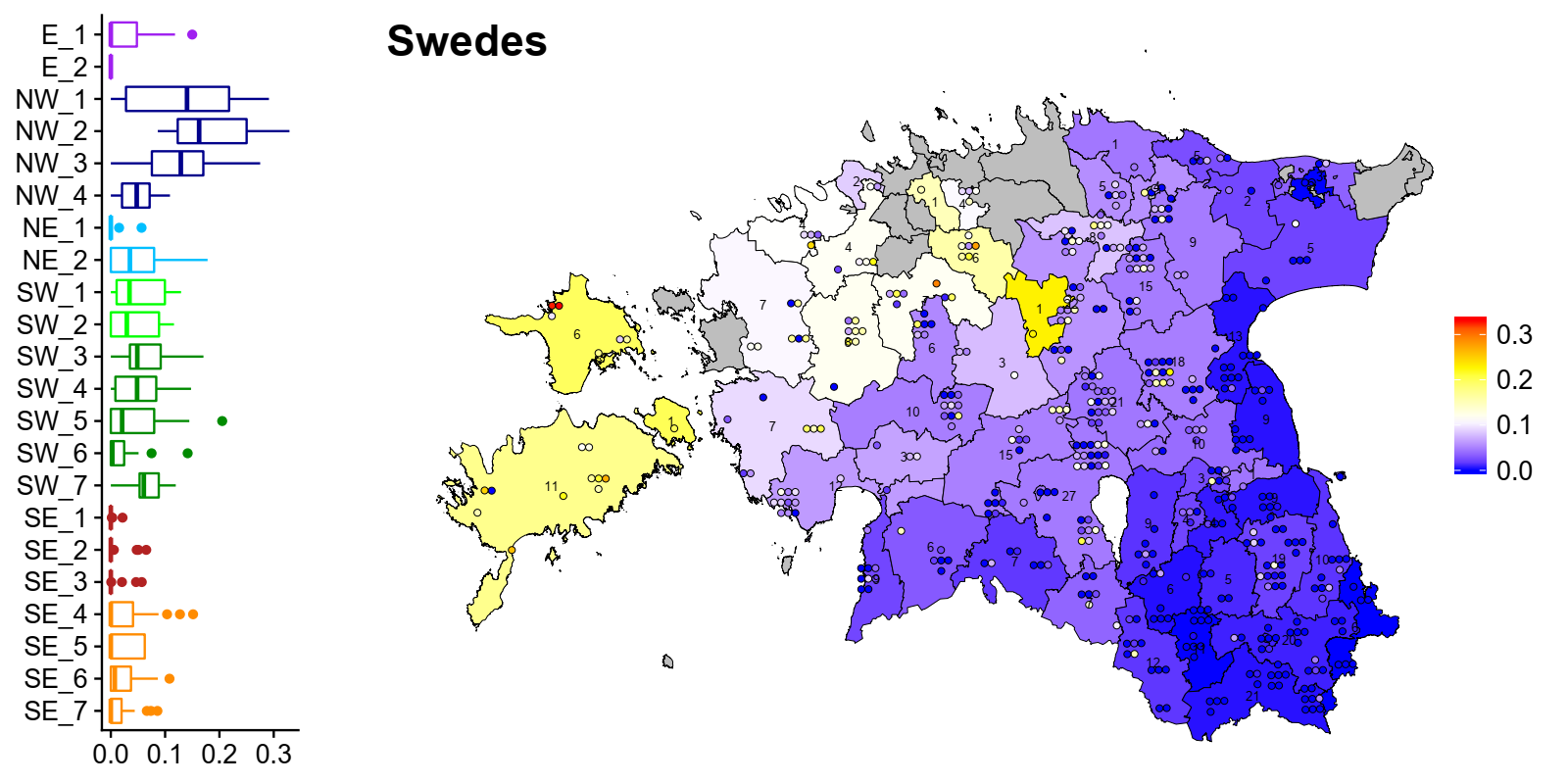


**Figure S3.5. Relative proportion of Swedish ancestry in the R50+ subset.** NNLS values for Swedes (“Sw” in figure S3.1) as surrogate source are shown. Two Estonian samples clustering together with Finns in figure S3.1 were excluded. NNLS values of individual samples are shown with coloured dots on the map. The fill of each parish reflects mean values of samples coming from the corresponding parish. The number of samples from each parish is shown on the map. Parishes with no samples in the R50+ dataset are filled with grey. On the left boxplots showing NNLS values for the clusters from figure 2 are presented. The boxes show 25^th^, 50^th^ and 75^th^ quantiles, while the whiskers show values within 1.5 times the inter-quantile range (IQR) of 25^th^ and 75^th^ quantiles. Individual dots show outliers (values out of the range shown by the whiskers). Clusters are labeled as in figure 2 referring to their geographic localization: E – East, NW – North-West, NE – North-East, SW – South-West, SE – South-East.

3.2 Levels of IBD segments sharing between Estonians and non-Estonian populations

We also assessed levels of IBD segments sharing, expressed as total genetic length of segments longer than 2 cM, between Estonian samples and the relevant non-Estonian groups revealed by CP/FS, namely Balts (Ba), Finns (F1 and F2 pooled together), Slavs (Sl) and Swedes (Sw) (figures S3.6 and S3.7). IBD segments were detected in the same sample that was used for CP/FS/GT (2305 Estonians and 600 non-Estonians) with *refined IBD* [9] and segments potentially broken by phasing and/or sequencing errors were merged together with *merge IBD* utility as described in Methods. Resulting segments shorter than 2 cM were filtered out and a pairwise sum of genetic length of the remaining ones was calculated. Next, for each Estonian sample, we averaged its’ IBD-sharing for each of the four non-Estonian groups, resulting in a 2305 x 4 matrix, which was used to create boxplots in figures S3.6 and S3.7 by grouping Estonian samples into clusters from either figure 2 or figure S3.1 respectively.


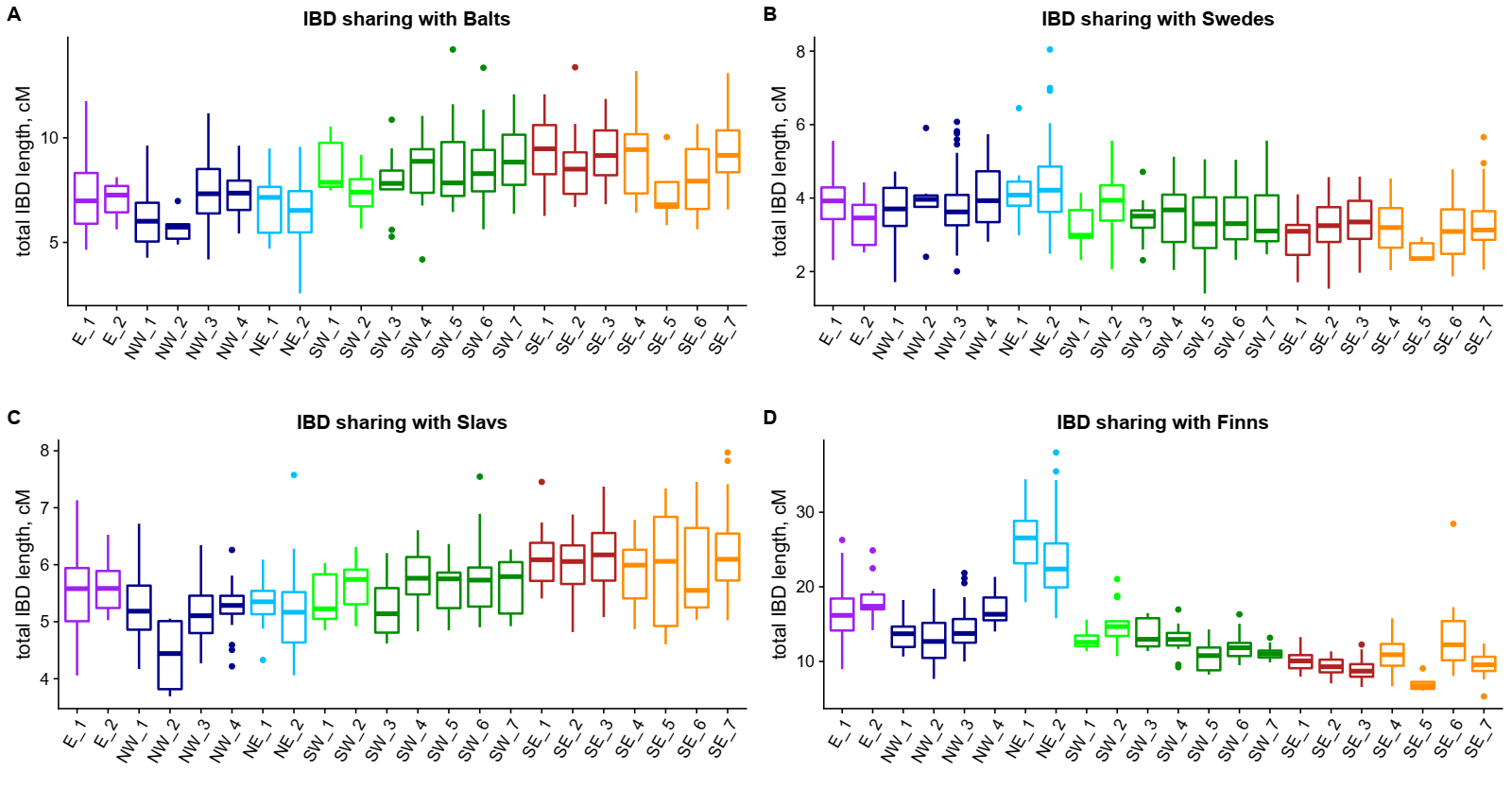


**Figure S3.6. Levels of IBD-sharing between R50+ Estonian clusters and neighbouring populations**. Box-plots show distribution of average total length of IBD segments in cM shared between Estonian from the R50+ subset and four non-Estonian groups: Balts (**A**), Swedes (**B**), Slavs (**C**) and Finns (**D**). Estonian are grouped as in figure 2 while the non-Estonian groups are the same as those used for NNLS (figure 3). The boxes show 25^th^, 50^th^ and 75^th^ quantiles, while the whiskers show values within 1.5 times the inter-quantile range (IQR) of 25^th^ and 75^th^ quantiles. Individual dots show outliers (values out of the range shown by the whiskers).


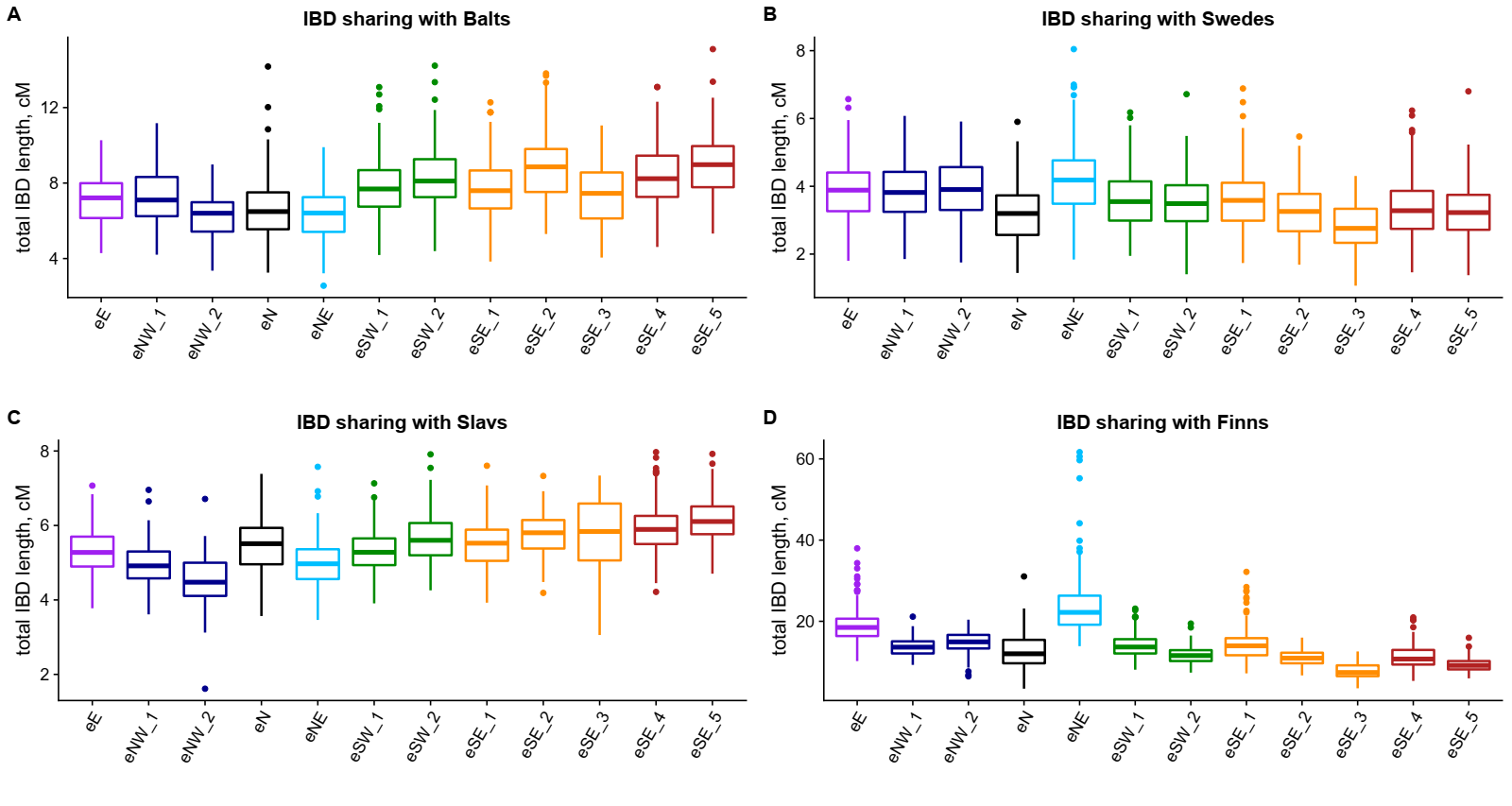


**Figure S3.7. Levels of IBD-sharing between clusters of the entire Estonians dataset and neighbouring populations**. Box-plots show distribution of average total length of IBD segments in cM shared between Estonian from the entire dataset and four non-Estonian groups: Balts (**A**), Swedes (**B**), Slavs (**C**) and Finns (**D**). Estonian are grouped as in figure 4 while the non-Estonian groups are the same as those used for NNLS (figure 3). The boxes show 25^th^, 50^th^ and 75^th^ quantiles, while the whiskers show values within 1.5 times the inter-quantile range (IQR) of 25^th^ and 75^th^ quantiles. Individual dots show outliers (values out of the range shown by the whiskers).

In general, the metric of IBD-sharing provided in figures S3.6 and S3.7 is less informative about patterns of genetic interactions between populations than NNLS because the former is more dependent on demographic histories of populations studied. For instance, IBD-sharing between Estonians and a lower Ne population like Finns would be higher compared to a higher Ne population like Slavs even if the actual migration rate was the same simply because IBD-sharing between Finnish individuals is higher than between Slavic, complicating inter-source comparisons. However, comparing with a given external population between Estonian clusters is more valid. The most important conclusion from such a comparison is an excess of IBD-sharing between Finns and members of the clusters NE_1, NE_2 (figure S3.6) and eNE (figure S3.7), in agreement with NNLS results (figure 3). In fact, the level of IBD-sharing between NE_2 members (21 cM on average) is comparable and for some samples even lower than their sharing with Finns, which can potentially indicate that this sharing is due to directional gene flow from the Finnish population to North-East Estonians and not due to common ancestry or gene flow in an opposite direction [7].

1. **Reconstructions of effective population size dynamics**

4.1 Effective population size dynamics reconstruction on simulated data

In our study we used *IBDNe*, an approach to estimate effective population size (Ne) in past generations using IBD segments shared between modern samples due to common ancestry a certain number of generations ago [8]. *IBDNe* is the method of choice for very recent time periods, allowing estimating Ne between 4 and up to 200 generations ago [8]. We first applied *IBDNe* to data simulated with *mspms* [20] to test its’ performance under complex demographic scenarios (figures S4.1 – S4.4). In particular, we wanted to test its’ power to detect recent (10-15 generations ago) and relatively short population decline episodes of various intensity either preceded or not by a period of growth, as well as effects of population subdivision, including differences in demography between subpopulations, and gene flow. We show, that if a short period of decline happens in between two periods of exponential growth, which is the case for Estonia based on historical records (figures 4c and S4.6), it would be revealed with *IBDNe* only if strong enough (figure S4.1). Also, note that in this scenario the *IBDNe* curve doesn’t drop as low as the actual simulated trajectory. On the other hand, if the population decline was not preceded by growth, even a 2x decline was readily detected with the curve reaching the real minimum of the effective population size (figure S4.2). Importantly, this figure also shows that under this scenario a severe bottleneck (blue curve in figure S4.2) does not erase the information about the pre-bottleneck population size. These observations illustrate that, as expected, short-term fluctuations in Ne cannot be accurately reconstructed by *IBDNe*, but it still can be used to detect short but drastic population collapses. Again, as anticipated, gene flow increases Ne estimates of the subpopulations for time points not only during the migration period but also preceding it (figure S4.3). These results also show that strong post-bottleneck migration may make the bottleneck less apparent (figure S4.3C). Finally, in agreement with previous theoretical reasoning [21] population subdivision has a complex influence on Ne estimates. In a simple case of a population consisting of isolated demes of the same size, *IBDNe* estimates for the total population is the sum of Ne values of the subpopulations when time points distant enough from the split time are considered (figure S4.3A). Intense gene flow makes each subpopulation effectively a random sample from the same gene pool and, hence, Ne values estimated for a subpopulation and for the total population appear to be the same (figure S4.3C). However, in the case of subpopulations with different demography, Ne inferred for the total population may in some cases be lower than the one for the bigger subpopulation (figure S4.4). As a rule of thumb, the shape of the Ne curve for the total population is influenced more by the trajectory of the lower Ne subpopulation given its’ share in the sample is big enough.

| 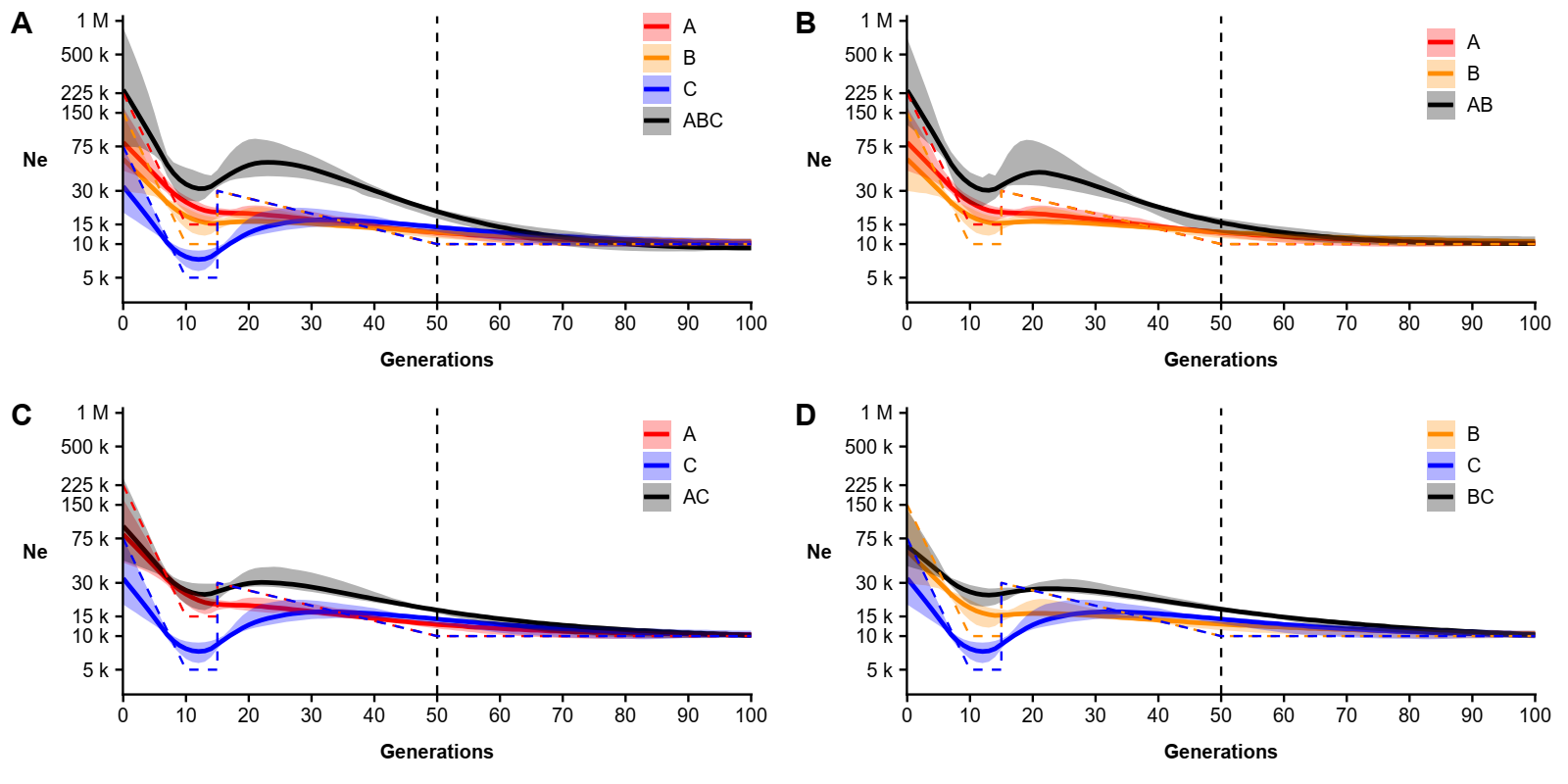 |
| --- |
| mspms 900 1 –seeds S1* S2* S3* –p 9 –t 50000 –r 40000 100000000 \  -I 3 300 300 300 \  -g 1 10832.2 –g 2 10832.2 –g 3 10832.2 \  -n 1 22.5 –n 2 15 –n 3 7.5 \  -en 0.00025 1 1.5 –en 0.00025 2 1 –en 0.00025 3 0.5 \  -en 0.000375 1 3 –en 0.000375 2 3 –en 0.000375 3 3 \  -eg 0.000375 1 1255.56 –eg 0.000375 2 1255.56 –eg 0.000375 3 1255.56 \  -en 0.00125 1 1 –en 0.00125 2 1 –en 0.00125 3 1 \  -ej 0.00125 3 1 –ej 0.00125 2 1 \  -en 0.00125 1 1  *S1-S3 – random seed values, different for each run  Each simulation was repeated 30 times each time with different random seed values to obtain 30 pairs of homologous 100Mb long “chromosomes” per “individual” |

**Figure S4.1. The effect of bottleneck depth on Ne trajectory reconstructions in a scenario with a pre-bottleneck exponential growth.** *IBDNe* was applied to simulated datasets (150 “individuals” each) from 3 populations that split 50 generations ago (dashed vertical line) with no subsequent migration as well as to different combinations thereof. Each population went through a bottleneck 10-15 generations ago, reaching a minimum size of 15, 10 and 5 thousands respectively, and then all recovered with equal growth rates. Dashed coloured lines show simulated trajectories while solid coloured lines show *IBDNe* results. Solid black lines show *IBDNe* results when using a dataset consisting of “individuals” from different populations (all three in panel A and pairwise combinations in panels B-D in proportions 50:50:50 and 75:75 respectively). The command used to run the simulation is provided below the plot.

| 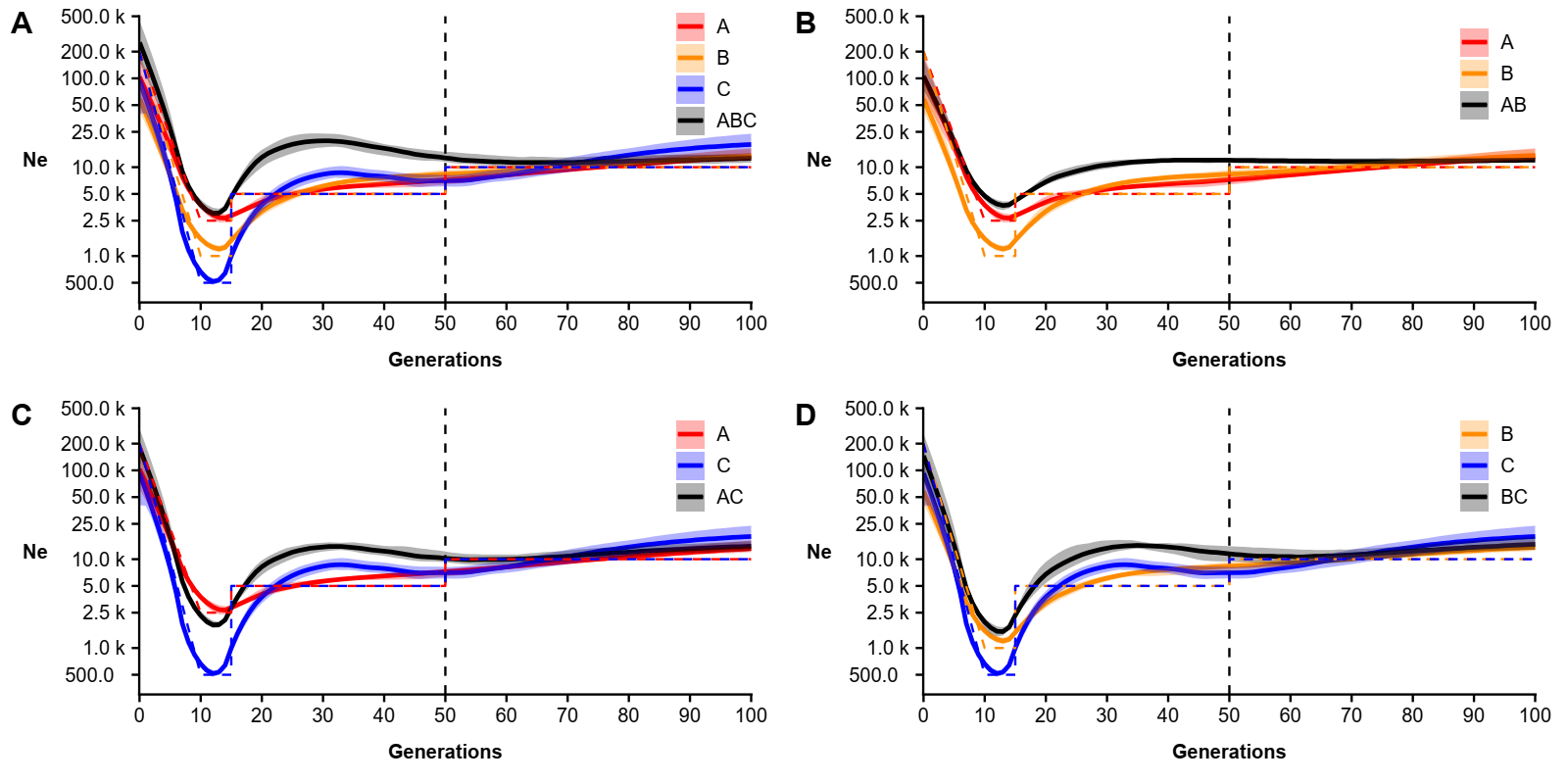 |
| --- |
| mspms 900 1 -seeds S1* S2* S3* -p 9 -t 50000 -r 40000 100000000 \  -I 3 300 300 300 \  -n 1 20 -n 2 20 -n 3 20 \  -g 1 17528.1 -g 2 21193.3 -g 3 23965.9 \  -eG 0.00025 0 \  -en 0.00025 1 0.25 -en 0.00025 2 0.1 -en 0.00025 3 0.05 \  -en 0.000375 1 0.5 -en 0.000375 2 0.5 -en 0.000375 3 0.5 \  -ej 0.00125 3 1 -ej 0.00125 2 1 \  -en 0.00125 1 1  *S1-S3 – random seed values, different for each run  Each simulation was repeated 30 times each time with different random seed values to obtain 30 pairs of homologous 100Mb long “chromosomes” per “individual” |

**Figure S4.2. The effect of bottleneck depth on Ne trajectory reconstructions with no pre-bottleneck growth.** *IBDNe* was applied to simulated datasets (150 “individuals” each) from 3 populations that split 50 generations ago (dashed vertical line) with no subsequent migration as well as to different combinations thereof. Each population went through a bottleneck 10-15 generations ago, reaching a minimum size of 2.5, 1 and 0.5 thousand respectively, and then each recovered to a size of 200 thousand. Dashed coloured lines show simulated trajectories while solid coloured lines show *IBDNe* results. Solid black lines show *IBDNe* results when using a dataset consisting of “individuals” from different populations (all three in panel A and pairwise combinations in panels B-D in proportions 50:50:50 and 75:75 respectively). The command used to run the simulation is provided below the plot.

| 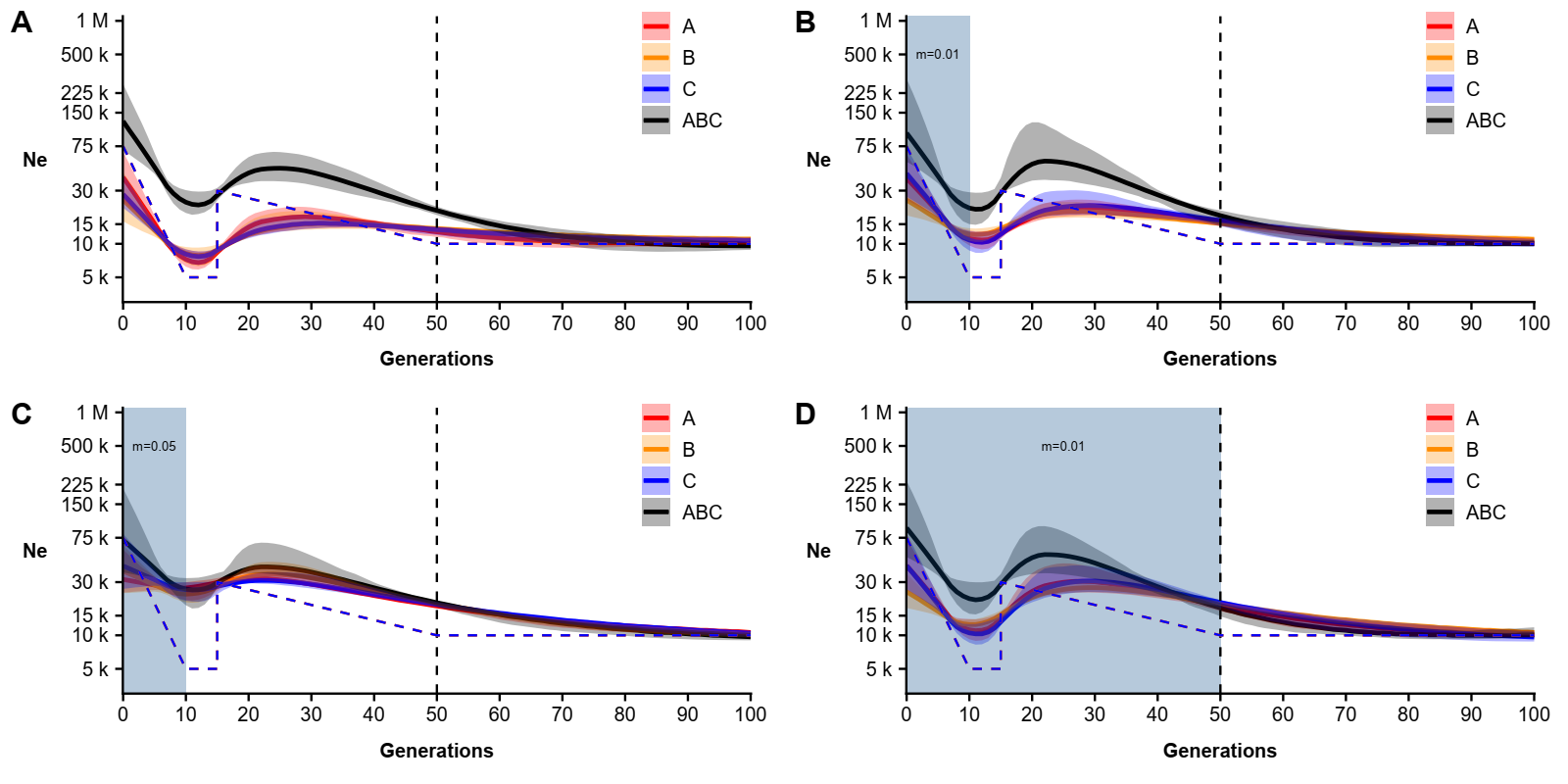 |
| --- |
| mspms 900 1 -seeds S1* S2* S3* -p 9 -t 50000 -r 40000 100000000 \  -I 3 300 300,300 \  -G 10832.2 \  -n 1 7.5 -n 2 7.5 -n 3 7.5 \  -ma M* M* M* M* M* M* \  -en 0.00025 1 0.5 -en 0.00025 2 0.5 -en 0.00025 3 0.5 \  -eM T* 0 \  -en 0.000375 1 3 -en 0.000375 2 3 -en 0.000375 3 3 \  -eg 0.000375 1 1255.56 -eg 0.000375 2 1255.56 -eg 0.000375 3 1255.56 \  -en 0.00125 1 1 -en 0.00125 2 1 -en 0.00125 3 1 \  -ej 0.00125 3 1 -ej 0.00125 2 1 \  -en 0.00125 1 1  *S1-S3 – random seed values, different for each chromosome  Each simulation was repeated 30 times each time with different random seed values to obtain 30 pairs of homologous 100Mb long “chromosomes” per “individual”  *M – migration rate, scaled in coalescent units, 0 in scenario A, 400 in B and D and 2000 in C  *T – time when migration rate is set to 0, scaled in coalescent units, 0.00025 in A, B and C and 0.000375 in D. |

**Figure S4.3. The effect of gene flow on Ne trajectory reconstructions.** *IBDNe* was applied to simulated datasets (150 “individuals” each) from 3 populations that split 50 generations ago (dashed vertical line) and went through a bottleneck 10-15 generations ago, reaching a minimum size of 5 thousand. Scenarios with no migration (A), symmetrical migrations at a rate of 0.01 and 0.05 during the last 10 generations (B and C) and at a rate of 0.01 during the last 50 generations (D) were simulated. Dashed coloured lines show simulated trajectories while solid coloured lines show *IBDNe* results. Solid black lines show *IBDNe* results when using a dataset consisting of “individuals” from all three populations in equal proportions (50:50:50). Shaded areas show time periods when migrations took place. The command used to run the simulation is provided below the plot.

| 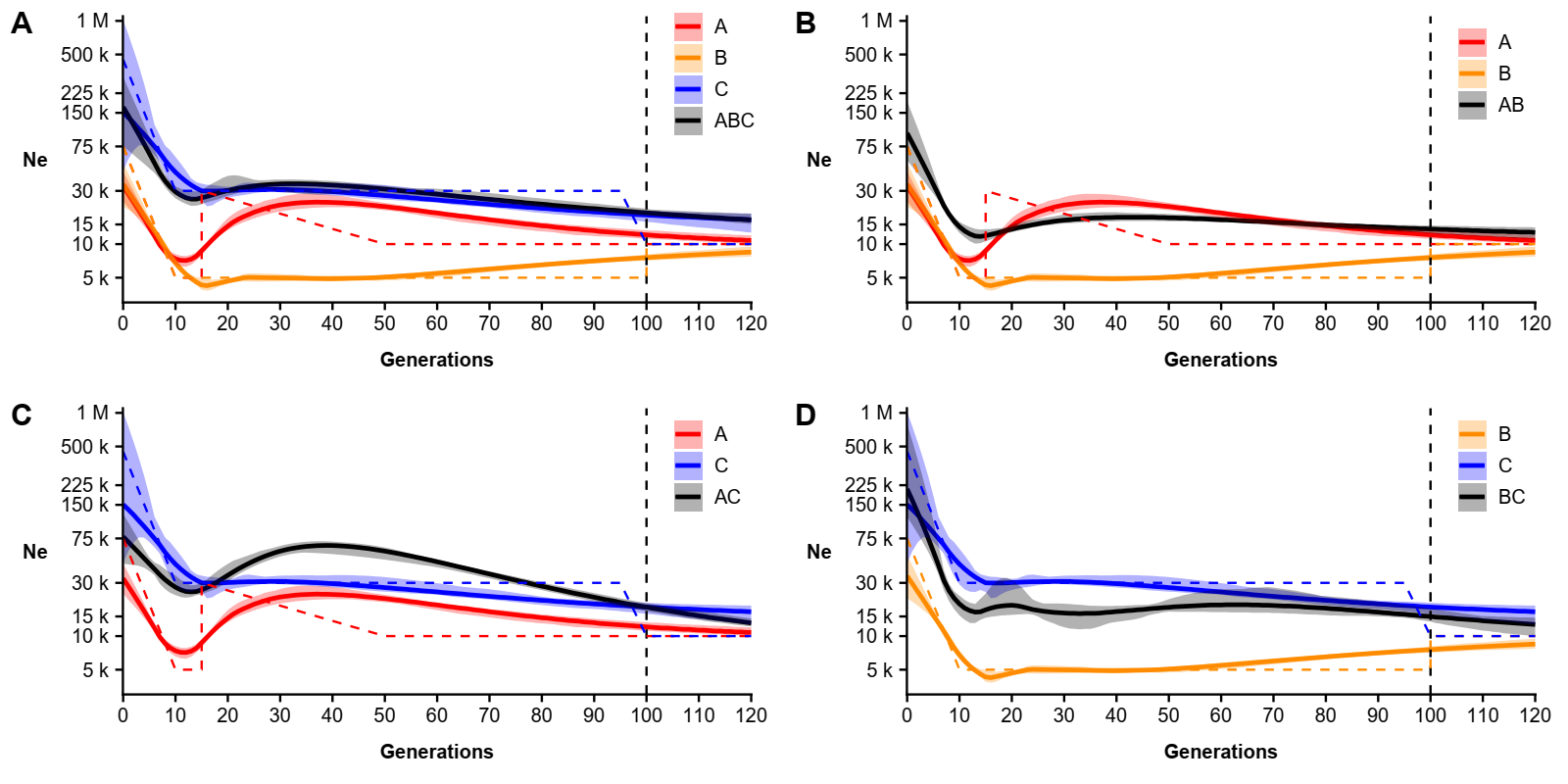 |
| --- |
| mspms 900 1 -seeds S1* S2* S3* -p 9 -t 50000 -r 40000 100000000 \  -I 3 300 300 300 \  -G 10832.2 \  -n 1 7.5 -n 2 7.5 -n 3 45 \  -en 0.00025 1 0.5 -en 0.00025 2 0.5 -en 0.00025 3 3 \  -en 0.000375 1 3 -en 0.000375 2 0.5 -en 0.000375 3 3 \  -eg 0.000375 1 516.994 \  -en 0.002375 3 3 \  -eg 0.002375 3 8788.9 \  -ej 0.0025 3 1 -ej 0.0025 2 1 \  -en 0.0025 1 1  *S1-S3 – random seed values, different for each chromosome  Each simulation was repeated 30 times each time with different random seed values to obtain 30 pairs of homologous 100Mb long “chromosomes” per “individual” |

**Figure S4.4. The effect of pooling together subpopulations with different demographic histories on Ne trajectory reconstructions.** *IBDNe* was applied to simulated datasets (150 “individuals” each) from 3 populations that split 100 generations ago (dashed vertical line) with no subsequent migration as well as to different combinations thereof. Dashed coloured lines show simulated trajectories while solid coloured lines show *IBDNe* results. Solid black lines show *IBDNe* results when using a dataset consisting of “individuals” from different populations (all three in panel A and pairwise combinations in panels B-D in proportions 50:50:50 and 75:75 respectively). The command used to run the simulation is provided below the plot.

4.2 Reconstructing effective population size dynamics in Estonia

We applied *IBDNe* to Estonian whole genome sequencing data as described in the Methods section. We first ran *IBDNe* on four of the clusters from the figure 4a chosen to represent geographically distinct regions of the country and observed substantial differences between the generated curves (figure 4c). Next, we tested whether the results for different clusters representing the same geographic region are consistent with each other by applying *IBDNe* to various clusters (figure S4.5). Most clusters corresponding to a certain region showed the same trends in Ne dynamics but differed in the depth of the 10-15 generations ago bottleneck. This might be explained by differences in levels of recent admixture between clusters with more admixed clusters showing less pronounced bottleneck (see figure S4.3 for the corresponding simulation). The only cluster that has an *IBDNe* curve with a clearly distinct shape as compared to other clusters from its region is eSE_2, which shows almost no recent growth, but higher Ne values between 10 and 40 generations ago. This can be explained by this cluster having admixed origin between SE and SW as suggested both by its geographical location and pattern of IBD-sharing (figure 4a,b). Note that in our simulations we also observed intense admixture after a bottleneck to result in an underestimation of recent growth (figure S4.3C).


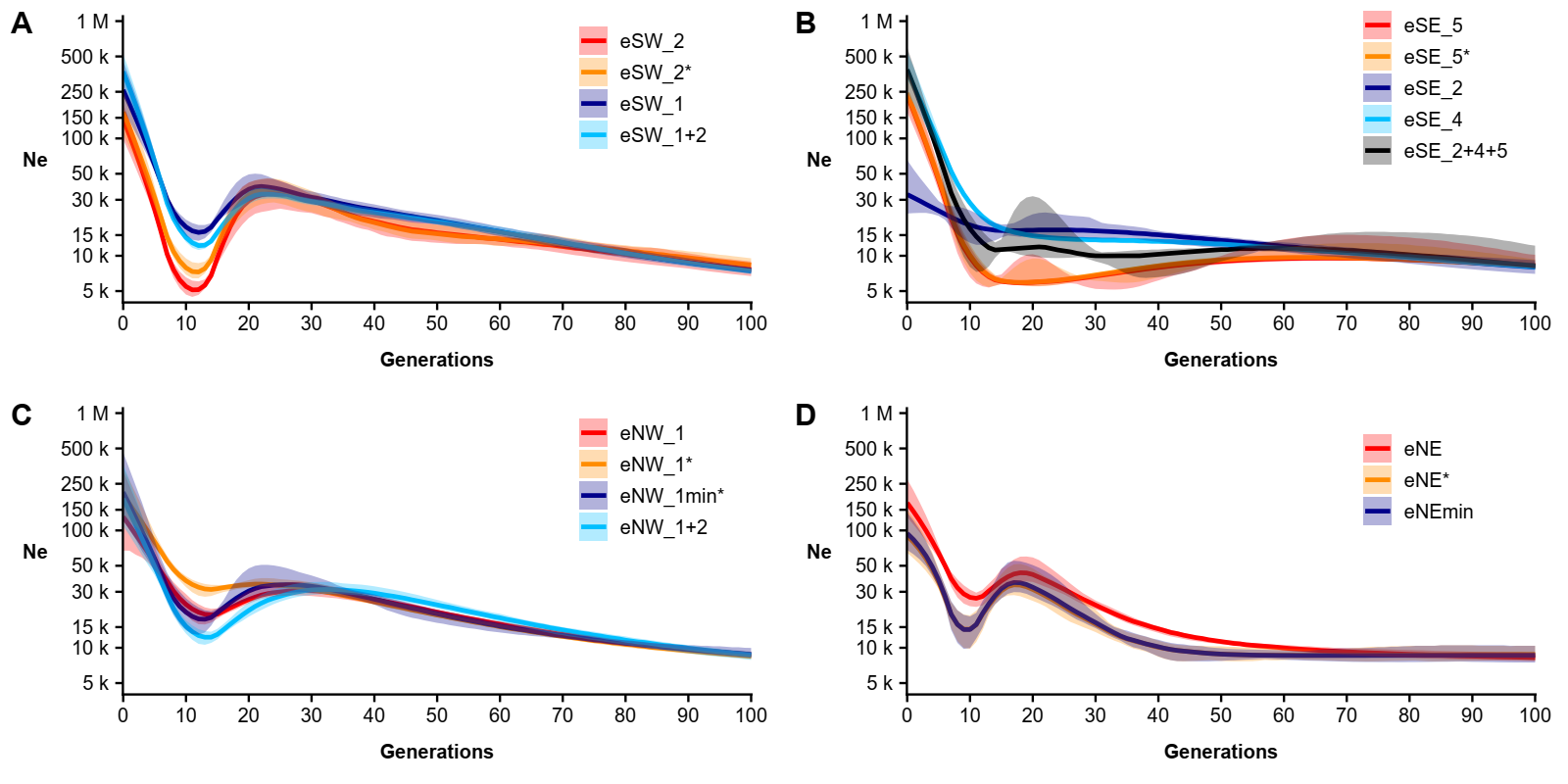


**Figure S4.5. Effective population size trajectory reconstructions for data subsets representing different regions of Estonia: South-West (A), South-East (B), North-West (C) and North-East (D).** Cluster names in the legends correspond to those from the first (figure 4a) or from the second IBD/FS run (figure S2.6, cluster names from the second run are marked with *). Notations like “eSW_1+2” mean that corresponding clusters (eSW_1 and eSW_2 in this case) were pooled together. As a group of about 125 samples (marked with a black line in figure S2.4) changed its cluster assignment between clusters eNE and eNW_1* between the runs we tested the effect of these individuals on Ne reconstructions by removing them from corresponding clusters resulting in groups denoted as eNEmin and eNW_1min*.

Having shown that differences between regional curves are genuine we went for a detailed comparison thereof. First, curves for all regions but South-East show evidence of a bottleneck around 10-15 generations ago. This is an agreement with historical data (figures 4c and S4.6) telling that the Estonian population declined 4-fold between 1550 and 1605 and almost 3-fold again between 1695 and 1712. The curve representing South-East stays constantly low before 10 generations ago and shows little evidence of the bottleneck and the preceding growth period (note, however, the shape of the confidence intervals). Our simulations (figure S4.1, populations A and B) show that in the case of “growth-decline-growth” dynamics with a relatively mild decline IBDNe smoothers the bottleneck and the preceding growth resulting in seemingly constant population size. Historical records suggest that the extreme South-Eastern part of modern Estonia, where most of the individuals falling into the eSE_5 cluster reside, did, in fact, suffer less than many other regions, losing about 50% of its’ population at the beginning of the 17^th^ century [13]. Thus we explain the curve for eSE_5 being different from others by less extreme population decline and likely also much slower growth before 15 generations ago.

| Year | Estonian population size | Source |
| --- | --- | --- |
| 1989 | 1,565,662 | Census results |
| 1959 | 1,196,791 | Census results |
| 1934 | 1,126,413 | Census results |
| 1911 | 1,086,000 | [22] |
| 1897 | 958,351 | Census results |
| 1881 | 881,455 | [22] |
| 1858 | 750,000 | [22] |
| 1800 | 500,000 | [23] |
| 1765 | 400,000 | [23] |
| 1712 | 170,000 | [23] |
| 1695 | 400,000 | [24] |
| 1640 | 140,000 | [24] |
| 1625 | 100,000 | [15] |
| 1605 | 75,000 | [25] |
| 1600 | 135,000 | [24] |
| 1550 | 300,000 | [14] |
| 1200 | 90,000-200,000 | [26] |

**Figure S4.6. Population size of Estonia based on historical data.** Census results were retrieved from https://www.stat.ee/dokumendid/62933. For other sources see references. For the year 1200 minimum and maximum estimates are given.

Second, we observe a high similarity between South-West and North-West curves (figure 4c, figure S4.5). As MAPS results suggest high levels of gene flow in Western and Northern Estonia (figure [S2.8A](https://www.dropbox.com/s/7awje8sau938q0a/ED3.pdf?dl=0)) we questioned whether SW and NW might represent effectively the same ancestral population 20 generations ago. As we see from our simulation results (figure S4.3D) intense gene flow makes Ne estimates based on samples from subpopulations and from the total population very close to each other. So we applied *IBDNe* to a joint sample of eSW_2 and eNW_1 and in fact observed very similar Ne values for the time period before 20 generations ago as those for the individual clusters (figure S4.6). As for the differences in the minimal Ne estimates at 10 generations ago between eNW_1 and eSW_2, it is likely to illustrate both stronger population decline in South-West [13] and more intense recent immigration to North-West.


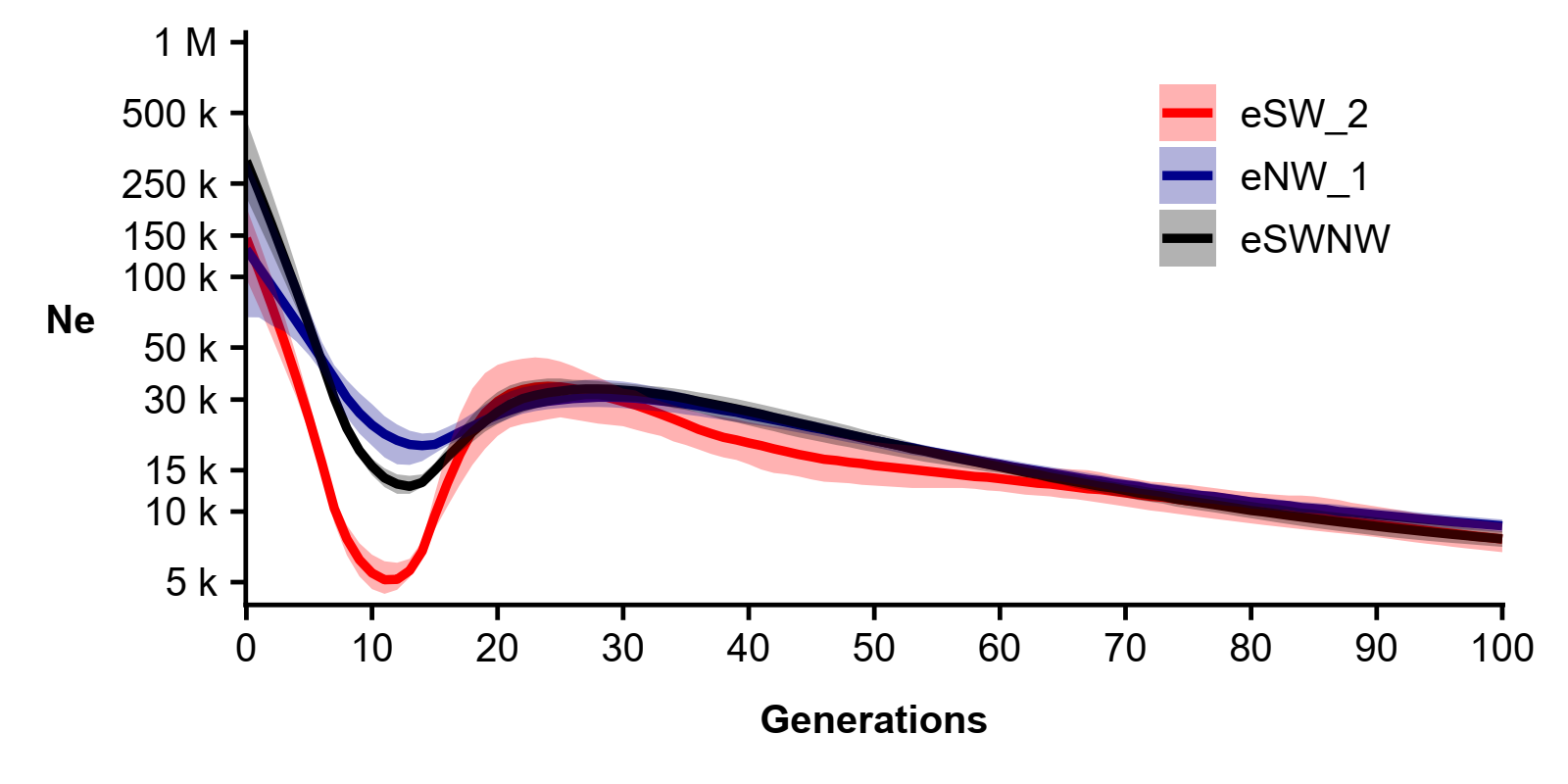


**Figure S4.6. Effective population size dynamics reconstruction from joined eSW_2 and eNW_1 sample (black curve).** Curves for eSW_2 and eNW_1 are provided for comparison.

Third, the curve corresponding to North-East Estonia also shows evidence for the described population decline but differs from South-West and North-West in a more recent onset of the pre-bottleneck growth as well as a more recent start of the decline (figures 4c and S4.5). We believe that the relatively strong admixture with Finns that we detect for North-East Estonians (figures 3 and S3.7) can explain this pattern. Indeed, the Finnish population has been shown to have an expansion start at around 35 generations ago while having an Ne below 10,000 before that [4].

4.3 Reconstructing effective population size dynamics in Britain

Having detected rather pronounced regional differences in effective population size dynamics in Estonia we wondered whether similar patterns can be observed in other populations and so to see if the phenomenon is not due to some characteristics of the Estonian population. To test for that we applied *IBDNe* to various groups from the People of the British Isles (PoBI) dataset [19] representing different geographic regions of the UK (table S4.1). And in fact, most regional groups have similar curve shapes with a constant growth until around 25-30 generations ago, a drop and then growth again starting from 10 generations ago. Interestingly enough, the population decline in England between 10 and 20 generations ago coincides with a period of recurrent plague episodes that started with the Black Death in 1348 [27]. However, the curves for Scotland and Wales show much slower growth before 15 generations ago and even no growth at all correspondingly (figure S4.7A). Note that the latter may be due to the growth being too subtle to be detected. Interestingly the case of Wales is somewhat similar to that of South-East Estonia: both have long-term low Ne and experienced very slow if any population growth until 10-15 generations ago, making each of them genetically rather distinct from other regional groups in their corresponding countries. Thus, based on the Estonian and the UK examples, we conclude that regional subpopulations within relatively small areas may have demographic histories that are qualitatively different. We also hypothesize that Ne dynamics may at least in part be driven by historical events such as wars, famine episodes and disease outbreaks, leading to an intriguing possibility of such events playing a role in shaping the genetic structure of modern human populations.


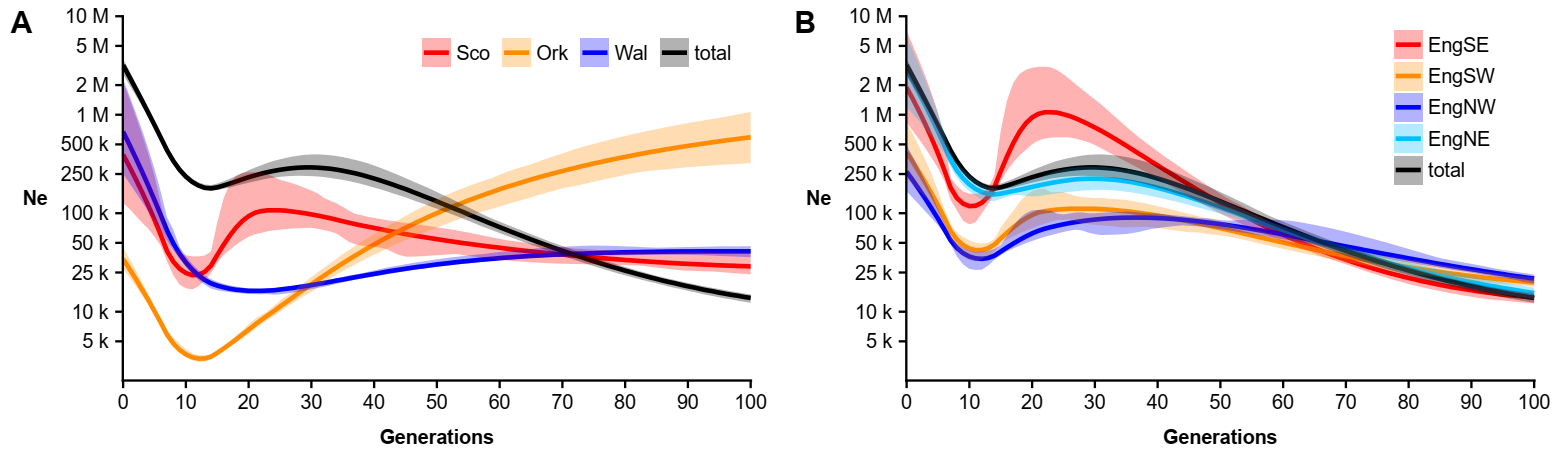


**Figure S4.7. Ne trajectories for different geographic groups of the PoBI dataset.** Sco – Scotland, Ork – Orkneys, Wal – Wales, EngSE – South-East England, EngSW – South-West England, EngNW – North-Western England, EngNE – North-Eastern England, total – all used groups pooled together. Plots are split into two panes to facilitate legibility. For counties included in each regional group and sample size see table S4.1.

4.4 Estimating actual census population size based on Ne

Effective population size estimates for past generations inferred from genetic data may be an additional and sometimes the only source of information about human population size at certain points in time and space. However, relationships between effective (Ne) and census (Nc) population sizes are complex and depend on a large number of factors, such as isolation/gene flow, population age and sex structure, mating patterns and variance in reproductive success to name a few [21,28,29]. Furthermore, such estimates are inevitably based on an assumption of population continuity, which is not always true. Nevertheless, here we propose an approach to convert coalescence-based estimates of Ne to census population size by accounting for genetic structure and external gene flow as well as by restricting the space of possible values of such parameters as inbreeding coefficient, sex ratio and variance in reproductive success to those reasonable for most human populations using the Estonian population as a test case. Specifically we start with the following formula (formula 1 in Methods)

$$N_{b(t)}=\frac{(1+Fis)}{4}\times(\frac{1}{(1-m)\times m}+DV)\times N_{e(t)}$$

The formula above yields the N_b,_ the number of breeding individuals (individuals capable of reproducing) at time t under the assumption of absence of gene flow and population structure, non-overlapping generations and equal variance of number of offspring between sexes. It is dependent on parameters such as m, Fis and DV (fraction of males, inbreeding coefficient and excess in variance of reproductive success compared to the Poisson distribution) that cannot be reliably estimated for each time bin. We therefore explored a range of plausible scenarios described by different values of m, Fis and DV based on the following assumptions: i) Fis calculated on chromosome 1 for contemporary human populations from the 1000 genomes dataset as well as for Estonians ranges from −0.016 to 0.004 (figure S4.9), leading to the conclusion that for most human populations the term (1+Fis) can be safely approximated to 1; ii) m, the relative fraction of reproducing males, must be comprised between 0.1 and 0.9, considering further polarizations of this parameter as implausible for our species; iii) DV, the difference between the expected and the observed variance in number of offspring per adult can be estimated to range between −1 and 3. The latter estimate was obtained by taking Poisson distributions constrained between 0 and 10 (considering 10 as the maximum number of surviving children per adult) with an average between 1 and 5, and by empirically inflating the most extreme bins (0 or 10 children per adult) 5-fold. Such an exercise yields DVs ranging between −0.2 and 2.5, which we conservatively rounded to −1 and 3, respectively. This range is also consistent with data from contemporary Estonians, available from the Estonian Biobank, and showing a DV of −0.76 based on 7,863 females born between 1900 and 1955 and in the age of menopause at the time of enrolment. Plugging the minimal and maximal values of m and DV outlined above gives the following range for N_b(t)_ which is the number of breeders (individuals capable of reproduction) at a given point in time

$${0.75\times N_{e(t)}\leq N}_{b(t)}\leq5.53\times N_{e(t)}$$

To convert Nb(t) to census size we need to include the individuals which are out of the reproducing age. To do so we divide the corresponding coefficients by 0.33. This value is supported by actual data on the Estonian population from the “Statistics Estonia” database ([http://andmebaas.stat.ee/Index.aspx?lang=en#](http://andmebaas.stat.ee/Index.aspx?lang=en)) showing that the fraction of people between 20 and 40 years old was between 0.33 to 0.38 during the period between 1970 and 2018.

Note that when using Ne as a proxy for actual population size one should keep in mind the potential effect of gene flow between populations. For example under a stepping stone model with constant population size and migration Ne estimated using samples from one deme is expected to increase when going back in time as more and more ancestors of sampled individuals would represent other demes^53^. In other words, coalescent-based Ne estimates reflect the number of ancestors of a sampled population, which may have lived in any location in space, rather than strictly the number of individuals in a given area at a given time point. So the match between our prediction and the historical estimates (figure 4d) may be attributed to i) our success in adequately controlling for events of recent gene flow and population structure; ii) the relatively recent time intervals considered, which limits the range of spatial interaction among the ancestors of contemporary Estonians.

There is a published approach to estimate ancestry-specific Ne in admixed populations [30] but we believe it to be not applicable in our case due to high genetic similarities between Estonians and the populations they experienced gene flow from. So we addressed the issues of external migrations and local differences in Ne by removing clusters with evidence of excessive gene flow from outside Estonia and/or of atypical Ne dynamics from our dataset. To do so we assessed the effect of including/excluding clusters with strong evidence for external admixture, low Ne or low sharing with other Estonians on Ne reconstructions for the total Estonian population (figures S4.8 and S4.9). We see that the overall impact of the dataset composition when reconstructing Ne dynamics for the total Estonian population is rather modest with the strongest effect observed for the period between 10 and 20 generations ago. We chose the Est1527 subset to represent Estonia when inferring actual census population size as it does not include clusters that show little IBD-sharing with other Estonian groups (eN, eSE_3), signals of strong external admixture (eNE) or atypically low Ne compared to other Estonian groups (eSE_5). We then converted those Ne values into Nc estimates as described in Methods.


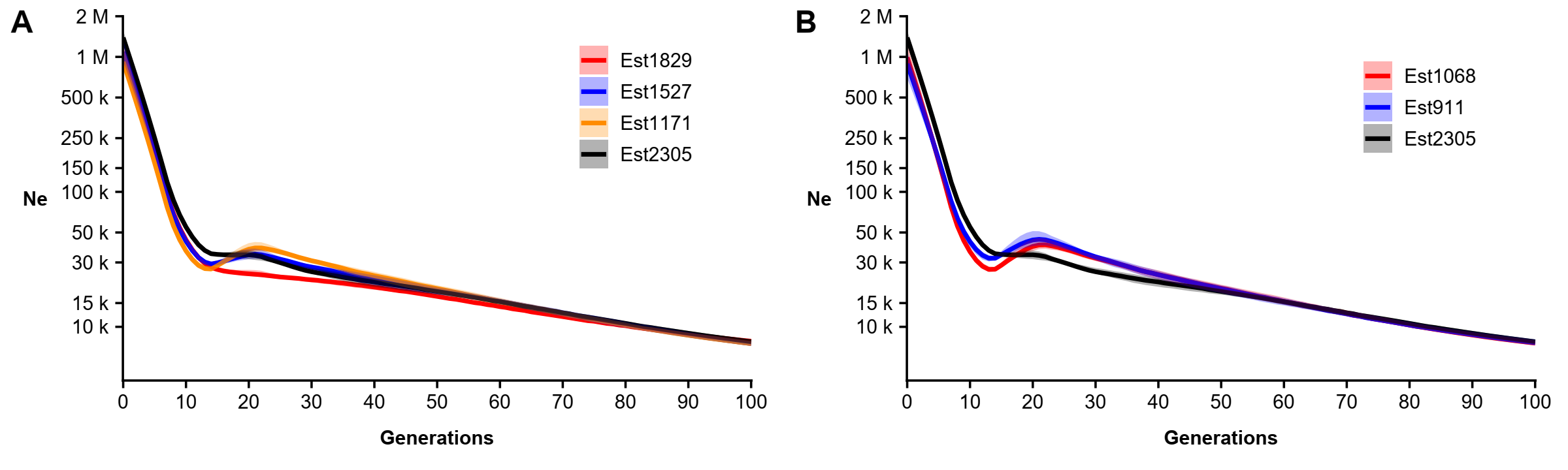


**Figure S4.8. Ne trajectories for different subsets of the Estonian dataset.** Composition of the subsets used can be found in figure S4.9. Plots are split into two panels to facilitate legibility. The curve for Est2305 (the entire dataset) is plotted in both panels for comparison.

| Cluster | Subsets | | | | | |
| --- | --- | --- | --- | --- | --- | --- |
|  | Est2305 | Est1829 | Est1527 | Est1171 | Est1068 | Est911 |
| eNW_1 | + | + | + | + | + | + |
| eNW_2 | + | + | + |  |  |  |
| eN | + |  |  |  |  |  |
| eNE | + |  |  |  |  |  |
| eE | + | + | + | + | + | + |
| eSW_1 | + | + | + | + | + | + |
| eSW_2 | + | + | + | + | + |  |
| eSE_1 | + | + | + | + | + | + |
| eSE_2 | + | + | + | + |  |  |
| eSE_3 | + |  |  |  |  |  |
| eSE_4 | + | + | + |  |  |  |
| eSE_5 | + | + |  |  |  |  |

**Figure S4.9. Samples subsets used in figure S4.8.** Clusters correspond to the first IBD/FS run (figure 4a). Clusters included into a given subset are marked with “+”.

One of the parameters needed to relate Nc to Ne is inbreeding coefficient Fis (formula 1 in Methods) which reflects deviations from expected heterozygosity due to non-random mating in the population. We estimated the value of Fis using PLINK-1.9 [31] for our sample of Estonians as well as for 7 populations from 1000 Genome Project [32] representing different continental groups and show that in the populations tested Fis is close to 0 (figure S4.10). The approaches used to account for differences in variance of reproductive success and male to female ratio are provided in the Methods.

| **Population** | **OH** | **EH** | **Fis** |
| --- | --- | --- | --- |
| **Estonians** | 0.064877 | 0.064714 | -0.00252 |
| **CEU** | 0.029549 | 0.02935 | -0.00677 |
| **CHB** | 0.028171 | 0.027727 | -0.01601 |
| **FIN** | 0.029549 | 0.029229 | -0.01093 |
| **YRI** | 0.039238 | 0.038891 | -0.00891 |
| **PEL** | 0.028148 | 0.028054 | -0.00334 |
| **CLM** | 0.031146 | 0.03128 | 0.004301 |
| **PJL** | 0.030503 | 0.030618 | 0.003753 |

**Figure S4.10. Inbreeding coefficient in different human populations.** OH - observed heterozygosity, EH - expected heterozygosity, Fis - inbreeding coefficient. All samples except for Estonians were taken from 1000 Genomes Project [32]. CEU - North-West Europeans, CHB - Chinese, FIN - Finns, YRI - Yorubu, PEL - Peruvians, CLM - Colombians, PJL - Punjabi Indians.

1. **Singleton density score selection scan**

In this study we use singleton density score (SDS) [33] to detect natural selection as this method catches signatures of very recent selection episodes and thus may potentially give insight into selective events that are specific to Estonia or even to certain Estonian regions.

5.1 Datasets

SDS analysis was applied to three Estonian datasets separately: the entire dataset of 2,305 samples (“entire dataset”) as well as to two regional subsets, South-East Estonia (“SE”, consisting of 1,029 samples belonging to clusters eSE_1 − eSE_5 in figure 4a) and the remaining 1,276 samples from other clusters (“nonSE”) (figure 4a). We used those two groups despite their heterogeneity because analyzing smaller clusters would result in a reduction of statistical power due to small sample sizes. All datasets were pre-processed in the same way including removing PCA-outliers (figure S1.1) and outliers in number of singletons per individual (figure S5.1). The final data set consisted of 2,076, 927, and 1,132 individuals for entire dataset, SE and nonSE, respectively. Input files for the SDS analysis were prepared and SDS analysis was run according to authors’ guidelines (Field et al., 2016 [33]; <https://github.com/yairf/SDS>). Standardization of raw SDS scores to correct for differences in derived allele frequency (DAF) was performed according to the original publication (Field et al., 2016 [33]). Approximate average tip branch length for the Estonian dataset used was estimated based on average number of singletons per genome as in (Field et al., 2016 [33]). Figure S5.1 shows the distribution of per-sample number of singletons in each dataset.

As mentioned in the Methods section, SDS was run on the genomes with the missing genotypes being imputed using Eagle v2.3 as otherwise all positions with at least one individual without a genotype could not be tested. Potentially imputation might result in false genotypes. In the case of singletons that might result in false-positives if missing genotypes in a singleton position is erroneously imputed to 0/0 or, although less likely, in false-negatives if some of the missing genotypes are erroneously imputed to 0/1. To estimate the potential impact of this effect on our results we looked into the distribution of the missing rate in singleton positions (figure S5.2). It can be seen that most of the singleton positions have a very low fraction of missing genotypes, with 95% of the positions having a missing genotype for 31 or fewer individuals corresponding to a missing rate of 1.5%. Thus we believe that our results are unlikely to be biased by imputation in singleton positions.


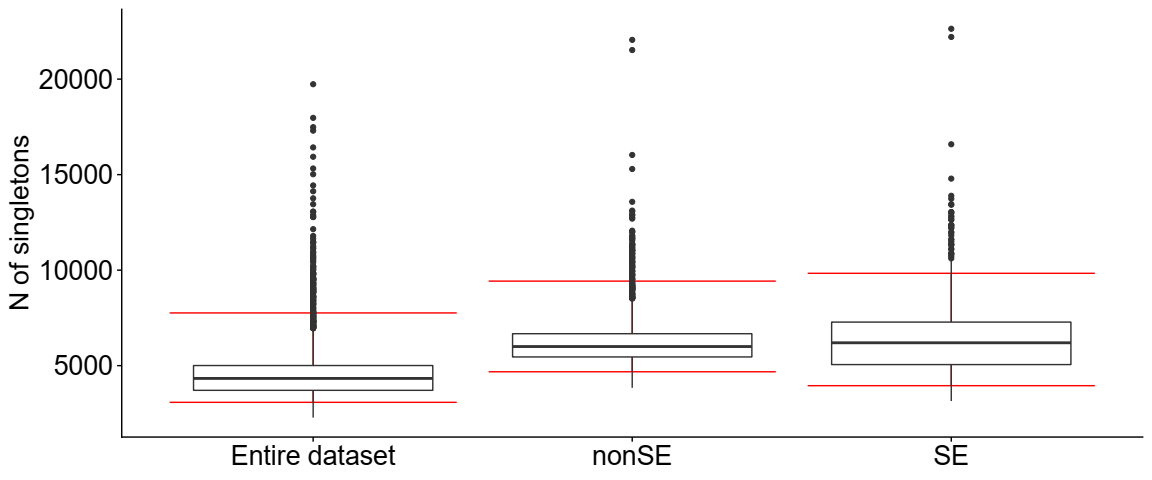


**Figure S5.1. Singleton count distribution in the datasets used for Singleton Density Score (SDS) analysis.** The boxplot shows the first, second and third quartiles with the whiskers showing the minimum and maximum values within ± 1.5 x the inter-quartile range, while the data points out of this range are shown as individual dots. The red horizontal lines show the 5^th^ and 95^th^ percentiles which were used as threshold when removing samples for the SDS analysis.

| **Percentile** | **Number of individuals with missing genotypes** |
| --- | --- |
| **50** | 2 |
| **75** | 4 |
| **95** | 31 |
| **99** | 164 |
| **99.9** | 1222 |

**Figure S5.2. Cumulative distribution of missing genotypes counts among singleton positions in the entire dataset after all filtering steps (2076 samples).**

5.2 Functional annotation of test SNPs and enrichment analyses

Combined Annotation-Dependent Depletion (CADD) [34] tool was used to assess a predicted functional effect of all test SNPs with SDS scores for three datasets. List of the genes associated with variants with absolute standardized SDS (sSDS) score higher than 2.5, CADD PHRED score higher than 10 and provided that the selected allele in SDS analysis and alternate allele in CADD annotation analysis are matching, was used in the enrichment analysis. In total, 638, 640 and 539 genes for the entire dataset, SE and nonSE, respectively, were subjected to the enrichment analysis with the *Enrichr* tool [35,36].

An alternative enrichment test using GWAS catalog categories (<http://www.ebi.ac.uk/gwas/home>; [37]) was also applied. Each category has been tested with a Wilcoxon-Mann-Whitney test for a difference in the distribution of absolute standardized SDS scores with respect to all other categories merged (i.e. only SNPs that are GWAS hits are considered). A one-tailed test was used thus testing for a given category being enriched in SNPs with absolute sSDS values deviating from zero compared to all other categories. In addition, GWAS enrichment test was re-calculated using two filters: a) removal SNPs in LD (for SNPs of the same category closer than 100kb from each other, pruning was performed by choosing the SNP with the lowest p-value and discarding the other) and b) exclusion of the HLA region (as the HLA region is highly enriched for GWAS catalog hits - harbors around 3% of hits - despite being around 0.2% of the human genome).

We also checked for correlation between absolute sSDS and absolute β scores which was done using SNPs passing the pruning filter. Note that in figure S5.5, conversely, raw values for both scores are used thus taking into account the directionality of the effect in GWAS and the allele (ancestral or derived) showing a signal of selection in SDS.

5.3 Results for the entire dataset

Distribution of genome-wide SDS scores indicates no single SNP hit when p-value of 5x10^-8^ is used as a significance threshold (figure 5a). Using a less strict threshold of 1x10^-5^ resulted in 33 SNPs from 10 genomic regions on chromosomes 2, 4, 6, 8, 9, 11, 13 and 20 (figure 5a, table S5.1). Out of these variants some lie in intergenic regions, whereas some are associated with certain genes (intronic, upstream variants,one 5' UTR variant of *CCL21* gene and one missense variant in MUCC2 gene) (table S5.1) and regions on chromosomes 4, 6, 9 and 11 were shown to be associated with expression levels of nearby genes (table S5.2; [38]). Although based on the rather high SDS p-values these SNPs can be at most viewed as suggestive candidate loci many of them are characterized by derived allele frequency (DAF) difference between Estonians and both UK and Finns concordant with SDS results: SNPs with DAF in Estonians higher than in the other two populations have high positive normalized SDS score and vice versa (table S5.1). This suggests that our SDS results do in fact capture genuine allele frequency changes that happened recently in the Estonian population. Out of these potential hits we consider regions 99.6 Mb on chromosome 4 and 34.7 Mb on chromosome 9 to be the most promising candidates for recent selection in the Estonian population as both are characterized by DAF out of the range between that of UK10K and the Finnish sample (table S5.1) and both are associated with expression levels of nearby genes (table S5.2).

It is interesting to draw parallels between our SDS results for the Estonian dataset and results by Field et al. [33] as both populations can be broadly considered as Northern European. First we looked at the correlation between normalized SDS scores in the UK10K dataset [33] and in the entire Estonian dataset (figure S5.3h) and observed a weak but significant correlation (Pearson’s correlation coefficient 0.12, p < 2.2x10^-16^).


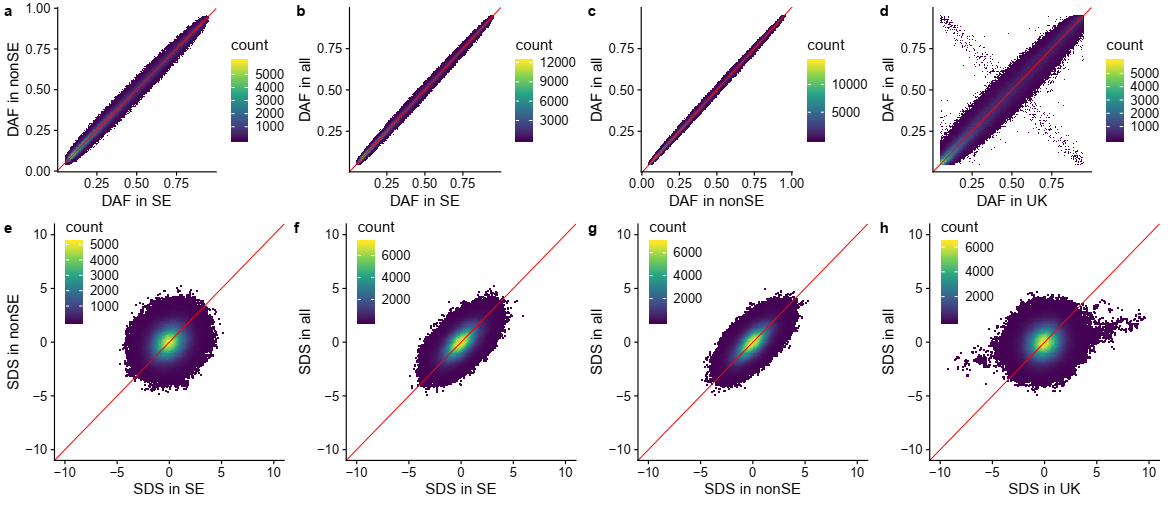


**Figure S5.3. Comparison of derived allele frequencies, (a-d) and normalized SDS scores (e-h) between different datasets.** Derived allele frequencies (**a-d**) and normalized SDS scores (**e-h**) were compared in SE vs nonSE (**a,e**), entire Estonian dataset vs SE (**b,f**), entire Estonian dataset vs nonSE (**c,g**) and entire Estonian dataset vs UK10K (**d,h**). SNPs forming a line perpendicular to the diagonal in panel **d** are annotated differently in terms of ancestral/derived allele due to different version of the ancestral genome used. The colour shows the density of data points in each region of the plot.

We next looked into the distribution of normalized SDS scores in the Estonian population binning the SNPs according to the normalized SDS score in the UK10K dataset (figure S5.4) to see if SNPs with strong evidence of selection in the UK population show any deviations from neutrality Estonians.


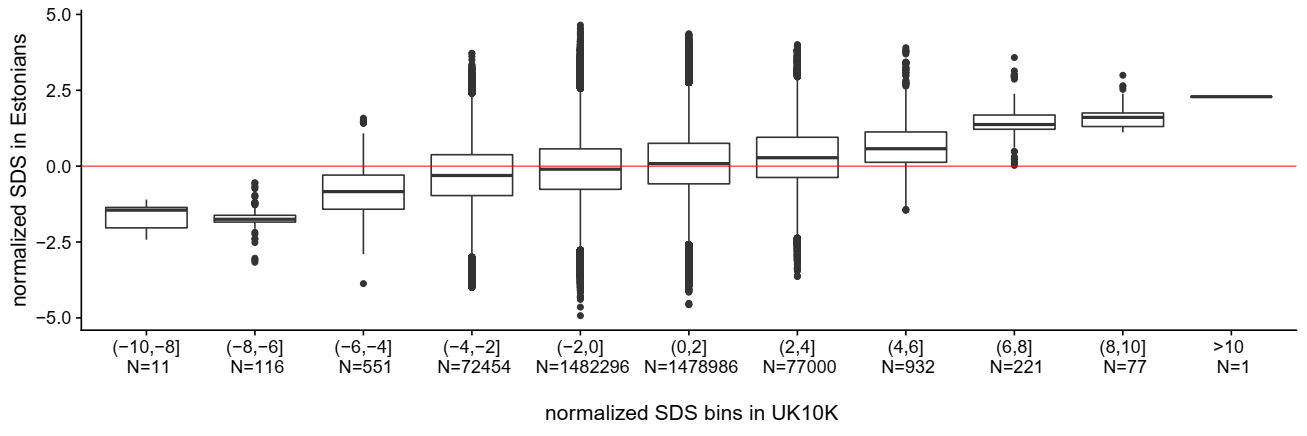


**Figure S5.4. Distribution of SDS scores in the entire Estonian dataset in different bins based on the SDS score in UK10K.** On both axes the normalized scores are used. Values after “N=” on the x axis show the number of SNPs in each bin.

As can be seen in figure S5.4, the bins with extreme SDS values in UK10K do, in fact, deviate from 0 in the corresponding direction while the central bins have medians close to 0 and rather high variance. This suggests that the SNPs reported to be under positive selection in the study of Field et al. do show some evidence of recent changes in allele frequency in the Estonian population but the signal is weak. Finally, we individually examined the three loci (LCT and WTFY4 genes and the MHC region) that had SNPs with a p-value below 5x10^-8^ in the UK10K dataset. LCT, one of the strongest selection signals detected in the study by Field et al. [33] and a classical example of positive selection in Europeans does not show strong evidence of being under positive selection in the Estonian population recently (the lowest p-value in the genomic region between 136 and 137 Mb of chromosome 2 being 5.5x10^-4^ corresponding to a normalized SDS score of -3.49).

Another strong selection signal in the UK10K dataset lies in the WDFY4 gene [33]. In the entire Estonian dataset the lowest p-value in this region (chr10:49.5-50.5 Mb) is 5x10^-4^ corresponding to the normalized SDS score of 3.47. However, in the SE subset (see below) there is a SNP in this region (rs2683608) with a p-value of 5.8x10^-6^ and normalized SDS of 4.53. (table S5.1, figure 5b). It is worth mentioning that DAF for this SNP in Estonia is lower than in the UK but higher than in Finns (table S5.1) so a higher proportion of Finnish ancestry in nonSE may explain the difference between the two Estonian subsets. There is also strong evidence for this SNP being associated with WDFY4 expression levels in the blood (table S5.2) further suggesting that it might indeed be under positive selection. There is a growing body of evidence that WDFY4 is involved in immune response toward viral and tumor antigens [39] as well as in autoimmune diseases [40,41,42] with direct indications of its’ involvement in B-cells development and differentiation [40].

Finally, the third genome-wide significant hit reported by Field et al. is the MHC locus. In the entire Estonian dataset there are five SNPs in this region with p-values below 1x10^-5^ in genes MUCC2 and PSORS1C1 (table S5.1).

Several factors may explain both the lack of genome-wide hits in our results and the difference from the results from Field et al. First, the statistical power of SDS depends on the sample size [33]. As the number of samples we use (2,076) is lower then what was used by Field et al. (3,195) this potentially could have resulted in less statistical power in our analysis. However this is unlikely to have a critical impact on our results as another study that applied SDS to a sample of 2,234 individuals from the Japanese population [43] has detected several genome-wide significant hits.

Second, SDS captures only recent selection within a time window roughly corresponding to the length of the terminal branches of the coalescent tree [33]. The length of terminal branches estimated in the original study ranged from 23-36 to 174 generation depending on the method used. In order to make a valid comparison between our sample and that from the UK population in terms of terminal branch length, we compared the mean number of singletons per sample between these datasets. This value is 8,200 in 3,195 UK samples controlling for low coverage and 4,500 in 2,305 Estonians, so applying mutation rate of 1.45x10^-8^ and assuming that 80% of the genome is properly mapped as was done by Field et al. [33] results in 112 generations for UK and 59 for Estonia. Bigger recent Ne and faster population growth during the last 100 generations in the UK compared to Estonia (figures S4.7 and S4.8) further support the idea of longer terminal branches in the UK10K dataset. As yet another way to compare the relative temporal windows that are analyzed in our study and the study of Field et al. we have tested for correlation between the difference in DAF between the Estonian and the UK10K datasets on one hand and the normalized SDS scores in either Estonians or UK10K (for SNPs with matching derived alleles in the two datasets). We observe that the SDS score in the Estonian dataset shows moderate correlation with the difference in DAF (Pearson correlation coefficient 0.46) while the SDS score in UK10K has low positive (0.05) correlation with it. Note that one would expect the value for the UK to be negative as the difference in DAF is defined as DAF (Estonia) – DAF(UK10K). All of this suggests that SDS scores in our study are more affected by very recent changes in allele frequencies that happened independently in the British and in the Estonian populations while SDS scores from Field et al. are mostly reflecting more ancient selection events. Hence we conclude that a) both studies, especially ours, focus on the very recent past when the Estonian and the British populations are expected to have independent histories; b) one might expect lower power to detect selection in our study as the time window in focus in the case of the Estonian dataset is shorter compared to UK10K.

Third, in the specific case of the LCT locus the frequency of the T allele of the causal SNP rs4988235 is lower in Estonia compared to that in the UK (0.50 and 0.74 [33] respectively) which points at rather pronounced differences in evolution of this locus in the two populations either due to differences in selection strength or time, admixture history and/or amount of genetic drift.

Given the reasoning above we conclude that although our analysis focuses on a rather short period of recent population history which results in some loss of statistical power generally the obtained SDS scores genuinely reflect changes in allele frequency. Thus we went on to see if we can detect any signatures of polygenic selection affecting many loci but having a weak effect on each particular SNP. To do so we tested whether certain functional categories are enriched in SNPs with extreme SDS values.

To this end we explored the GWAS catalog [37] to test whether there are categories that are enriched for variants with high standardized SDS scores. First, we analyzed 31,155 SNPs in our set that have annotations in GWAS catalog and found that at FDR <5% there are 12 categories enriched for variants with high absolute sSDS scores (table S5.4). Next, to control whether SNPs in LD will inflate the association we removed all SNPs belonging to the same category and closer than 100kb to each other. Repeating the test with these conditions, 8 out of 12 categories still hold significance, most of them being related to lung diseases or autoimmune diseases (table S5.4). However, if we further reduce the test set by removing the HLA region (chr6:28-34 Mb) no category passes the significance level at 5% FDR and one category, “feeling guilty”, stays at FDR equal to 25%. Thus we conclude that previous results are mainly driven by variants within the HLA locus which is known to show evidence of natural selection, including results by Field et al. and may indicate pleiotropic effects or linkage between selected and trait-associated SNPs.

If we analyze the correlation between SDS score and effect size of the SNPs according to GWAS catalog, in the form of absolute β score, we obtain a sense of how much the selection strength corresponds to the biologic effect on a certain trait (table S5.4). Considering significant categories at FDR <5% we again observe the strongest correlation for “lung cancer” related traits namely “lung cancer in ever smokers” and “squamous cell lung carcinoma”. When plotting SDS and β scores for SNPs associated with those categories, SNPs with strongly causative derived alleles show the highest SDS scores (figure S5.5). Other categories appear in this analysis as well, but given their low number of hits they can be considered only suggestive (table S5.4).


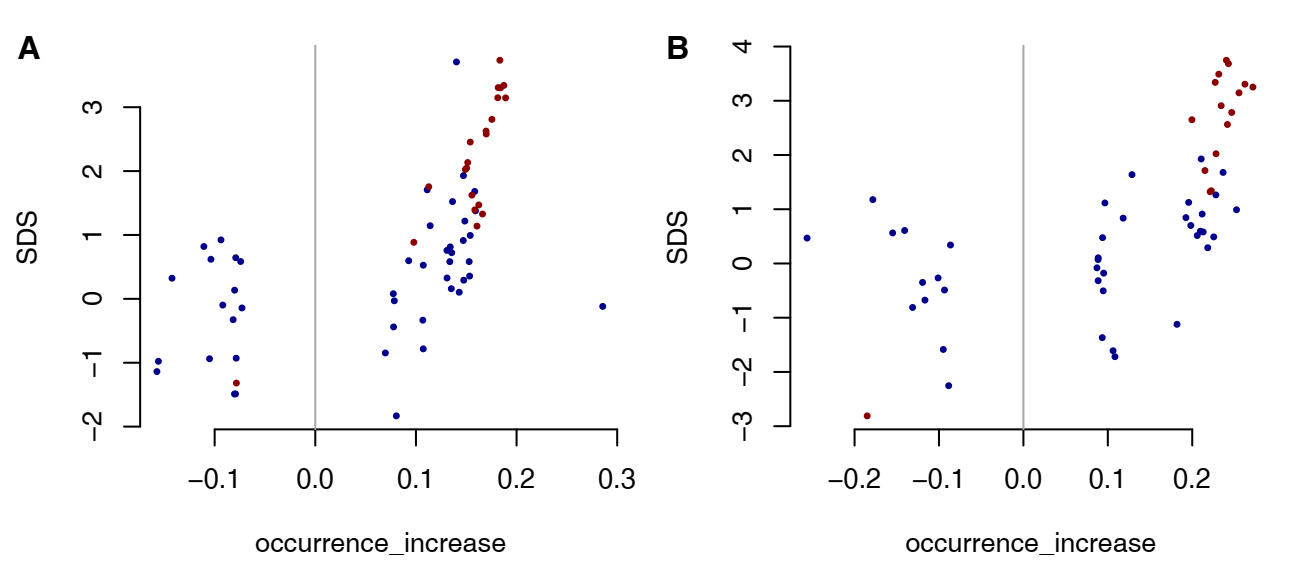


**Figure S5.5.** Lung cancer in ever smokers (A) and squamous cell lung carcinoma (B) GWAS catalog hits correlation with SDS. SNPs, represented as dots, were distance-pruned as specified in section 5.2, red dots depict SNPs falling in the HLA region. SDS is computed on the entire Estonian dataset and β scores are the effect of the derived allele.

Alternatively, CADD tool was used to assign scaled C-scores for each test SNPs (in other words, to rank the variant’s effect with respect to all possible substitutions in the human genome) [34]. We restricted our dataset to variants with CADD scores higher than 10 and SDS scores higher than 2.5, thus focusing on those that have stronger effect and that experience (recent) increase in frequency, and scored 637 genes that are associated with those SNPs for further “enrichment” test (table S5.7). We used the *Enrichr* tool [35,36] that allows analyzing various annotation categories and rank those that are enriched for the genes from our list. The top phenotypes from the Disease/Drug sub-category “dbGaP” (database for Genotypes and Phenotypes) are related to body height, weight as well as to various categories related to metabolism and heart phenotypes (table S5.8).

5.4 Regional differences in the SDS results in Estonians

Range of analyses done in our study suggests South East Estonian region to be differentiated from the rest of the today’s country (figures 2, 4). This motivated us to run the selection test separately on two subsets – South East (SE) and the rest of Estonians (nonSE) – questioning whether regional genetic differences affect also SDS results. The two subsets, 927 samples in SE and 1,132 in nonSE, are comparable in terms of the time we focus on as the mean number of singletons per individual after removing outliers is very similar in the two datasets used: 6,146 in SE and 6,105 in nonSE (figure S5.1), which roughly corresponds to potential selection acting during the last 80 generations. Based on our MAPS (figure S2.8) and IBDNe results (figure 4c) during most of this time SE and nonSE were partially isolated and had a partially independent demographic history. We also observe some differences in DAF between the two subsets (figure S5.3) with 5% of the SNPs having a difference in DAF bigger than 3.9%. When comparing normalized SDS scores between SE and nonSE we see that those are only weakly correlated (Pearson’s correlation coefficient 0.24, p-value < 2.2x10^-16^) suggesting again that we are dealing with an extremely recent and narrow time window.

Looking at the hits in each of the subsets we reveal 61 SNPs in 34 genomic regions with the p-value below 1x10^-5^ in one of the subsets but not in the other (table S5.1). Out of these the most promising signals of differential selection in the two Estonian subsets include five regions: 104.92 − 104.96 Mb on chromosome 2, 71.91 − 72.42 Mb on chromosome 5, 139.94 – 140.78 Mb and 146.68 − 146.70 Mb on chromosome 6 and 88.39 – 88.54 Mb on chromosome 11 as besides low SDS p-values in the SE subset the corresponding variants are characterized by Fst between SE and nonSE above 0.011 which corresponds to the 99.9 percentile of the genome-wide Fst distribution (table S5.1).

One of those regions, region 146.68 − 146.70 Mb on chromosome 6 lies within an intron of the GRM1 gene and includes two SNPs, rs75386033 and rs79907158, which are the only SNPs in our results with SDS p-values below 5x10^-8^.The frequency of derived alleles at these positions in SE (10%) is higher than in nonSE (6%) (table S5.1) and other European populations from reported in GnomAD [44] (https://gnomad.broadinstitute.org), where it does not exceed 4.5%. Searching for reported phenotypic effects in https://www.ncbi.nlm.nih.gov/snp database did not give any result. We also checked these two SNPs as well as five others that are in linkage disequilibrium with them (R2 > 0.5 in the entire Estonian dataset) for known cis-eQTL effects in the eQTLGen Consortium database [38] (http://www.eqtlgen.org/, access date 02.08.2019) (table S5.3). This revealed that one SNP, rs362848, effects expression levels of EPM2A gene (FDR=1.31x10-5), however its’ effect is modest compared to many other loci in this chromosomal region (figure S5.4). So there is no strong evidence for any phenotypic effect of rs75386033 and rs79907158, however, one can hypothesize that EPM2A expression may be effected by cis combinations of alleles at different loci and the detected SNPs mark a certain selected haplotype but we currently lack the data to test this hypothesis.

Analysis of the remaining four regions shows that three of them, on chromosome 5, 6 (139.94 – 140.78 Mb) and 11 include SNPs associated with expression levels of genes FCHO2, CITED2 and CTSC respectively (table S5.2). Besides that the region on chromosome 11 lies within the intron of the GRM5 which has been previously detected as a locus under positive selection associated with skin pigmentation [45].

As mentioned in the previous section, when studying the two subsets independently we also observe a signal in the WDFY4 gene which was one of the genome-wide significant hits in the study by Field et al [33]. However, despite the rather low SDS p-value in SE it shows almost no difference in DAF between the two Estonian subsets which does not support the idea of differential selection on this SNP.


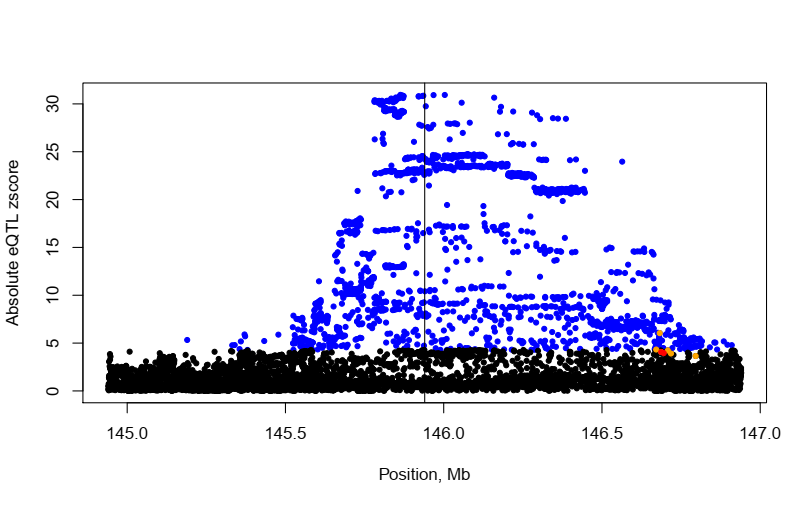


**Figure S5.4. Absolute *cis*-eQTL z-scores for SNPs in the region 144940105-146939903 on chromosome 6.** Loci, polymorphic in the entire Estonian dataset are shown. Significant SNPs at 0.05 FDR are shown in blue, the rest are in black. The SNP detected in the SDS scan (rs75386033 and rs79907158) are in red, while SNPs in LD with those (R^2^ > 0.5) are in orange. Vertical black line shows *EPM2A* transcription start site. The data is retrieved from the eQTLGen Consortium database [38] (<http://www.eqtlgen.org/>, access date 02.08.2019).

Next, we performed enrichment tests for the two datasets in the same manner as described in section 5.3. GWAS catalog enrichment test revealed only few categories to be enriched in variants with high absolute values of SDS in SE and nonSE when controlling for linkage between variants and applying a multiple-testing correction. Those categories include “Pneumonia” in SE and 9 traits with 5 lung disease-related ones including “Pneumonia” in nonSE, similar to the results in the entire dataset (tables S5.5 and S5.6). When looking for correlation between the absolute sSDS values and GWAS β we see that the category “Lung cancer in ever smokers” shows a significant correlation in both SE and nonSE while “Bone mineralization density” is specific to SE and “Squamous cell lung carcinoma” and “Feeling fed-up” are specific to nonSE. Again, both in the case of enrichment and correlation, none of the categories hold if HLA is removed and FDR < 5% is used as a threshold indicating that the results are being driven by the HLA locus (tables S5.5 and S5.6).

To conclude, we observe enrichment for different, although partially related, categories (lung diseases) in the two dataset with nonSE showing almost exactly the same results as the entire dataset while SE shares only one category with the entire dataset in each test and has a private category “Bone mineralization density” with and FDR-corrected p-value equal to 0.0505 for correlation between absolute sSDS and absolute β after all corrections applied. Although it is unclear if this trait really experienced recent natural selection as our results suggest, if this is a genuine signal it would have not been detected if only the entire dataset was analyzed.

Within an alternative enrichment test, lists of 640 genes in SE and 539 genes in nonSE (associated with variants with CADD scores > 10 and SDS scores >2.5) were subjected to *Enrichr* tool [35,36] to reveal which functional categories are enriched for those genes in each dataset (tables S5.9 – S5.12). We found that top entries within the dbGaP category overlap between the two datasets as well as with the results in the entire dataset, with categories related to metabolism and anthropometry showing the highest enrichment.

We conclude that given the demographic history of the Estonian population and specifically its low Ne when applying SDS we focus on a rather short period of the population’s past. On one hand this compromises our statistical power to detect selection. On the other hand, however, our results may at least suggest that very recent selection has been acting differentially between the two Estonian subpopulations studies. Although we detect no strong selection signal, combining different lines of evidence, we can name loci 99.6 Mb on chromosome 4, 72.4 Mb on chromosome 5, 139.9 Mb on chromosome 6, 146.6 Mb on chromosome 6 (GRM1 gene), 34.7 Mb on chromosome 9 (CCL21 gene), and 88.5 Mb on chromosome 11 (GRM5 gene) as the likeliest targets for recent selection in the Estonian population due to the corresponding SNPs having low SDS p-values, relatively big differences in DAF with other European population and/or between Estonian subsets, many of those SNPs being eQTLs and the GRM5 gene being previously detected in scans for positive selection.

1. **References**

1. N. Patterson, A. L. Price, D. Reich, Population structure and eigenanalysis. *PLoS Genet.* **2**, e190 (2006).

2. M. Nelis, T. Esko, R. Mägi, F. Zimprich, A. Zimprich, D. Toncheva, S. Karachanak, T. Piskáčková, I. Balaščák, L. Peltonen, E. Jakkula, K. Rehnström, M. Lathrop, S. Heath, P. Galan, S. Schreiber, T. Meitinger, A. Pfeufer, H.-E. Wichmann, B. Melegh, N. Polgár, D. Toniolo, P. Gasparini, P. D’Adamo, J. Klovins, L. Nikitina-Zake, V. Kučinskas, J. Kasnauskienė, J. Lubinski, T. Debniak, S. Limborska, A. Khrunin, X. Estivill, R. Rabionet, S. Marsal, A. Julià, S. E. Antonarakis, S. Deutsch, C. Borel, H. Attar, M. Gagnebin, M. Macek, M. Krawczak, M. Remm, A. Metspalu, Genetic Structure of Europeans: A View from the North–East. *PLOS ONE*. **4**, e5472 (2009).

3. T. Haller, L. Leitsalu, K. Fischer, M.-L. Nuotio, T. Esko, D. I. Boomsma, K. O. Kyvik, T. D. Spector, M. Perola, A. Metspalu, MixFit: Methodology for Computing Ancestry-Related Genetic Scores at the Individual Level and Its Application to the Estonian and Finnish Population Studies. *PLoS ONE*. **12** (2017), doi:10.1371/journal.pone.0170325.

4. A. R. Martin, K. J. Karczewski, S. Kerminen, M. I. Kurki, A.-P. Sarin, M. Artomov, J. G. Eriksson, T. Esko, G. Genovese, A. S. Havulinna, J. Kaprio, A. Konradi, L. Korányi, A. Kostareva, M. Männikkö, A. Metspalu, M. Perola, R. B. Prasad, O. Raitakari, O. Rotar, V. Salomaa, L. Groop, A. Palotie, B. M. Neale, S. Ripatti, M. Pirinen, M. J. Daly, Haplotype Sharing Provides Insights into Fine-Scale Population History and Disease in Finland. *Am. J. Hum. Genet.* **102**, 760–775 (2018).

5. P. Danecek, A. Auton, G. Abecasis, C. A. Albers, E. Banks, M. A. DePristo, R. E. Handsaker, G. Lunter, G. T. Marth, S. T. Sherry, G. McVean, R. Durbin, 1000 Genomes Project Analysis Group, The variant call format and VCFtools. *Bioinforma. Oxf. Engl.* **27**, 2156–2158 (2011).

6. D. J. Lawson, G. Hellenthal, S. Myers, D. Falush, Inference of Population Structure using Dense Haplotype Data. *PLoS Genet*. **8**, e1002453 (2012).

7. C. Bycroft, C. Fernandez-Rozadilla, C. Ruiz-Ponte, I. Quintela, Á. Carracedo, P. Donnelly, S. Myers, Patterns of genetic differentiation and the footprints of historical migrations in the Iberian Peninsula. *Nat. Commun.* **10**, 551 (2019).

8. S. R. Browning, B. L. Browning, Accurate Non-parametric Estimation of Recent Effective Population Size from Segments of Identity by Descent. *Am. J. Hum. Genet.* **97**, 404–418 (2015).

9. B. L. Browning, S. R. Browning, Improving the accuracy and efficiency of identity-by-descent detection in population data. *Genetics*. **194**, 459–471 (2013).

10. D. J. Lawson, D. Falush, Population identification using genetic data. *Annu. Rev. Genomics Hum. Genet.* **13**, 337–361 (2012).

11. B. L. Browning, S. R. Browning, Detecting Identity by Descent and Estimating Genotype Error Rates in Sequence Data. *Am. J. Hum. Genet.* **93**, 840–851 (2013).

12. H. Al-Asadi, D. Petkova, M. Stephens, J. Novembre, Estimating recent migration and population-size surfaces. *PLOS Genet.* **15**, e1007908 (2019).

13. J. Vasar, in *Eesti rahva ajalugu* (Tartu, 1934), vol. 9, p. 932.

14. E. Tarvel, in *Eesti talurahva ajalugu* (Tallinn, 1992), vol. 1, pp. 136–147.

15. O. Liiv, in *Eesti majandusajalugu*, H. Sepp, Ed. (Tartu, 1937), p. 163.

16. G. Hellenthal, G. B. J. Busby, G. Band, J. F. Wilson, C. Capelli, D. Falush, S. Myers, A genetic atlas of human admixture history. *Science*. **343**, 747–751 (2014).

17. G. Hudjashov, T. M. Karafet, D. J. Lawson, S. Downey, O. Savina, H. Sudoyo, J. S. Lansing, M. F. Hammer, M. P. Cox, Complex Patterns of Admixture across the Indonesian Archipelago. *Mol. Biol. Evol.* **34**, 2439–2452 (2017).

18. A. Loit, Invandringen från Finland till Baltikum under 1600-talet. *Hist. Tidskr. För Finl.* (1982), pp. 194–195.

19. S. Leslie, B. Winney, G. Hellenthal, D. Davison, A. Boumertit, T. Day, K. Hutnik, E. C. Royrvik, B. Cunliffe, Wellcome Trust Case Control Consortium 2, International Multiple Sclerosis Genetics Consortium, D. J. Lawson, D. Falush, C. Freeman, M. Pirinen, S. Myers, M. Robinson, P. Donnelly, W. Bodmer, The fine-scale genetic structure of the British population. *Nature*. **519**, 309–314 (2015).

20. J. Kelleher, A. M. Etheridge, G. McVean, Efficient Coalescent Simulation and Genealogical Analysis for Large Sample Sizes. *PLOS Comput. Biol.* **12**, e1004842 (2016).

21. M. C. Whitlock, N. H. Barton, The Effective Size of a Subdivided Population. *Genetics*. **146**, 427–441 (1997).

22. S. Vahtre, in *Eesti ajalugu. Pärisorjuse kaotamisest Vabadussõjani* (Tartu, 2010), vol. 5, pp. 60–62.

23. H. Palli, *Eesti rahvastiku ajalugu 1712-1799* (Tallinn, 1997).

24. H. Palli, *Eesti rahvastiku ajalugu aastani 1712* (Tallinn, 1996).

25. M. Seppel, 1601.-1603. aasta näljahäda Eestimaal. *Tuna Ajalookultuuri Ajak.* (2014), pp. 25–43.

26. E. Tarvel, in *Mokslotyra. Eina nuo 1998 m.* (Vilnius, 1999), pp. 83–85.

27. J. Goldberg, in *The Black Death in England*, M. Ormrod, P. G. Lindley, Eds. (Stamford, 1996), p. English.

28. V. Laporte, B. Charlesworth, Effective Population Size and Population Subdivision in Demographically Structured Populations. *Genetics*. **162**, 501–519 (2002).

29. B. Charlesworth, Fundamental concepts in genetics: effective population size and patterns of molecular evolution and variation. *Nat. Rev. Genet.* **10**, 195–205 (2009).

30. S. R. Browning, B. L. Browning, M. L. Daviglus, R. A. Durazo-Arvizu, N. Schneiderman, R. C. Kaplan, C. C. Laurie, Ancestry-specific recent effective population size in the Americas. *PLoS Genet.* **14**, e1007385 (2018).

31. S. Purcell, B. Neale, K. Todd-Brown, L. Thomas, M. A. R. Ferreira, D. Bender, J. Maller, P. Sklar, P. I. W. de Bakker, M. J. Daly, P. C. Sham, PLINK: a tool set for whole-genome association and population-based linkage analyses. *Am. J. Hum. Genet.* **81**, 559–575 (2007).

32. The 1000 Genomes Project Consortium, A global reference for human genetic variation. *Nature*. **526**, 68–74 (2015).

33. Y. Field, E. A. Boyle, N. Telis, Z. Gao, K. J. Gaulton, D. Golan, L. Yengo, G. Rocheleau, P. Froguel, M. I. McCarthy, J. K. Pritchard, Detection of human adaptation during the past 2000 years. *Science*. **354**, 760–764 (2016).

34. M. Kircher, D. M. Witten, P. Jain, B. J. O’Roak, G. M. Cooper, J. Shendure, A general framework for estimating the relative pathogenicity of human genetic variants. *Nat. Genet.* **46**, 310–315 (2014).

35. E. Y. Chen, C. M. Tan, Y. Kou, Q. Duan, Z. Wang, G. V. Meirelles, N. R. Clark, A. Ma’ayan, Enrichr: interactive and collaborative HTML5 gene list enrichment analysis tool. *BMC Bioinformatics*. **14**, 128 (2013).

36. M. V. Kuleshov, M. R. Jones, A. D. Rouillard, N. F. Fernandez, Q. Duan, Z. Wang, S. Koplev, S. L. Jenkins, K. M. Jagodnik, A. Lachmann, M. G. McDermott, C. D. Monteiro, G. W. Gundersen, A. Ma’ayan, Enrichr: a comprehensive gene set enrichment analysis web server 2016 update. *Nucleic Acids Res.* **44**, W90-97 (2016).

37. J. MacArthur, E. Bowler, M. Cerezo, L. Gil, P. Hall, E. Hastings, H. Junkins, A. McMahon, A. Milano, J. Morales, Z. M. Pendlington, D. Welter, T. Burdett, L. Hindorff, P. Flicek, F. Cunningham, H. Parkinson, The new NHGRI-EBI Catalog of published genome-wide association studies (GWAS Catalog). *Nucleic Acids Res.* **45**, D896–D901 (2017).

38. U. Võsa, A. Claringbould, H.-J. Westra, M. J. Bonder, P. Deelen, B. Zeng, H. Kirsten, A. Saha, R. Kreuzhuber, S. Kasela, N. Pervjakova, I. Alvaes, M.-J. Fave, M. Agbessi, M. Christiansen, R. Jansen, I. Seppälä, L. Tong, A. Teumer, K. Schramm, G. Hemani, J. Verlouw, H. Yaghootkar, R. Sönmez, A. Brown, V. Kukushkina, A. Kalnapenkis, S. Rüeger, E. Porcu, J. Kronberg-Guzman, J. Kettunen, J. Powell, B. Lee, F. Zhang, W. Arindrarto, F. Beutner, B. Consortium, H. Brugge, i2QTL Consortium, J. Dmitreva, M. Elansary, B. P. Fairfax, M. Georges, B. T. Heijmans, M. Kähönen, Y. Kim, J. C. Knight, P. Kovacs, K. Krohn, S. Li, M. Loeffler, U. M. Marigorta, H. Mei, Y. Momozawa, M. Müller-Nurasyid, M. Nauck, M. Nivard, B. Penninx, J. Pritchard, O. Raitakari, O. Rotzchke, E. P. Slagboom, C. D. A. Stehouwer, M. Stumvoll, P. Sullivan, P. A. C. ‘t Hoen, J. Thiery, A. Tönjes, J. van Dongen, M. van Iterson, J. Veldink, U. Völker, C. Wijmenga, M. Swertz, A. Andiappan, G. W. Montgomery, S. Ripatti, M. Perola, Z. Kutalik, E. Dermitzakis, S. Bergmann, T. Frayling, J. van Meurs, H. Prokisch, H. Ahsan, B. Pierce, T. Lehtimäki, D. Boomsma, B. M. Psaty, S. A. Gharib, P. Awadalla, L. Milani, W. Ouwehand, K. Downes, O. Stegle, A. Battle, J. Yang, P. M. Visscher, M. Scholz, G. Gibson, T. Esko, L. Franke, Unraveling the polygenic architecture of complex traits using blood eQTL metaanalysis. *bioRxiv*, 447367 (2018).

39. D. J. Theisen, J. T. Davidson, C. G. Briseño, M. Gargaro, E. J. Lauron, Q. Wang, P. Desai, V. Durai, P. Bagadia, J. R. Brickner, W. L. Beatty, H. W. Virgin, W. E. Gillanders, N. Mosammaparast, M. S. Diamond, L. D. Sibley, W. Yokoyama, R. D. Schreiber, T. L. Murphy, K. M. Murphy, WDFY4 is required for cross-presentation in response to viral and tumor antigens. *Science*. **362**, 694–699 (2018).

40. Q. Yuan, Y. Li, J. Li, X. Bian, F. Long, R. Duan, X. Ma, F. Gao, S. Gao, S. Wei, X. Li, W. Sun, Q. Liu, WDFY4 Is Involved in Symptoms of Systemic Lupus Erythematosus by Modulating B Cell Fate via Noncanonical Autophagy. *J. Immunol. Baltim. Md 1950*. **201**, 2570–2578 (2018).

41. Y. Zhang, L. Bo, H. Zhang, C. Zhuang, R. Liu, E26 Transformation-Specific-1 (ETS1) and WDFY Family Member 4 (WDFY4) Polymorphisms in Chinese Patients with Rheumatoid Arthritis. *Int. J. Mol. Sci.* **15**, 2712–2721 (2014).

42. L. A. McIntosh, M. C. Marion, M. Sudman, M. E. Comeau, M. L. Becker, J. F. Bohnsack, T. E. Fingerlin, T. A. Griffin, J. P. Haas, D. J. Lovell, L. A. Maier, P. A. Nigrovic, S. Prahalad, M. Punaro, C. D. Rosé, C. A. Wallace, C. A. Wise, H. Moncrieffe, T. D. Howard, C. D. Langefeld, S. D. Thompson, Genome-Wide Association Meta-Analysis Reveals Novel Juvenile Idiopathic Arthritis Susceptibility Loci. *Arthritis Rheumatol. Hoboken NJ*. **69**, 2222–2232 (2017).

43. [Y.Okada](https://www.ncbi.nlm.nih.gov/pubmed/?term=Okada%20Y%5BAuthor%5D&cauthor=true&cauthor_uid=29691385), [Y. Momozawa](https://www.ncbi.nlm.nih.gov/pubmed/?term=Momozawa%20Y%5BAuthor%5D&cauthor=true&cauthor_uid=29691385), [S. Sakaue](https://www.ncbi.nlm.nih.gov/pubmed/?term=Sakaue%20S%5BAuthor%5D&cauthor=true&cauthor_uid=29691385), [M. Kanai](https://www.ncbi.nlm.nih.gov/pubmed/?term=Kanai%20M%5BAuthor%5D&cauthor=true&cauthor_uid=29691385), [K. Ishigaki](https://www.ncbi.nlm.nih.gov/pubmed/?term=Ishigaki%20K%5BAuthor%5D&cauthor=true&cauthor_uid=29691385), [M. Akiyama](https://www.ncbi.nlm.nih.gov/pubmed/?term=Akiyama%20M%5BAuthor%5D&cauthor=true&cauthor_uid=29691385), [T. Kishikawa](https://www.ncbi.nlm.nih.gov/pubmed/?term=Kishikawa%20T%5BAuthor%5D&cauthor=true&cauthor_uid=29691385), [Y Arai](https://www.ncbi.nlm.nih.gov/pubmed/?term=Arai%20Y%5BAuthor%5D&cauthor=true&cauthor_uid=29691385), [T Sasaki](https://www.ncbi.nlm.nih.gov/pubmed/?term=Sasaki%20T%5BAuthor%5D&cauthor=true&cauthor_uid=29691385), [K. Kosaki](https://www.ncbi.nlm.nih.gov/pubmed/?term=Kosaki%20K%5BAuthor%5D&cauthor=true&cauthor_uid=29691385), [M. Suematsu](https://www.ncbi.nlm.nih.gov/pubmed/?term=Suematsu%20M%5BAuthor%5D&cauthor=true&cauthor_uid=29691385), [K. Matsuda](https://www.ncbi.nlm.nih.gov/pubmed/?term=Matsuda%20K%5BAuthor%5D&cauthor=true&cauthor_uid=29691385), [K. Yamamoto](https://www.ncbi.nlm.nih.gov/pubmed/?term=Yamamoto%20K%5BAuthor%5D&cauthor=true&cauthor_uid=29691385), [M. Kubo](https://www.ncbi.nlm.nih.gov/pubmed/?term=Kubo%20M%5BAuthor%5D&cauthor=true&cauthor_uid=29691385), [N. Hirose](https://www.ncbi.nlm.nih.gov/pubmed/?term=Hirose%20N%5BAuthor%5D&cauthor=true&cauthor_uid=29691385), [Y Kamatani](https://www.ncbi.nlm.nih.gov/pubmed/?term=Kamatani%20Y%5BAuthor%5D&cauthor=true&cauthor_uid=29691385). Deep whole-genome sequencing reveals recent selection signatures linked to evolution and disease risk of Japanese. *Nat Commun*. 9 1631 (2018).

44. K. J. Karczewski, L. C. Francioli, G. Tiao, B. B. Cummings, J. Alföldi, Q. Wang, R. L. Collins, K. M. Laricchia, A. Ganna, D. P. Birnbaum, L. D. Gauthier, H. Brand, M. Solomonson, N. A. Watts, D. Rhodes, M. Singer-Berk, E. G. Seaby, J. A. Kosmicki, R. K. Walters, K. Tashman, Y. Farjoun, E. Banks, T. Poterba, A. Wang, C. Seed, N. Whiffin, J. X. Chong, K. E. Samocha, E. Pierce-Hoffman, Z. Zappala, A. H. O’Donnell-Luria, E. V. Minikel, B. Weisburd, M. Lek, J. S. Ware, C. Vittal, I. M. Armean, L. Bergelson, K. Cibulskis, K. M. Connolly, M. Covarrubias, S. Donnelly, S. Ferriera, S. Gabriel, J. Gentry, N. Gupta, T. Jeandet, D. Kaplan, C. Llanwarne, R. Munshi, S. Novod, N. Petrillo, D. Roazen, V. Ruano-Rubio, A. Saltzman, M. Schleicher, J. Soto, K. Tibbetts, C. Tolonen, G. Wade, M. E. Talkowski, T. G. A. D. Consortium, B. M. Neale, M. J. Daly, D. G. MacArthur, Variation across 141,456 human exomes and genomes reveals the spectrum of loss-of-function intolerance across human protein-coding genes. *bioRxiv*, 531210 (2019).

45. P. F. Palamara, J. Terhorst, Yu. S. Song, A. L. Price. High-throughput inference of pairwise coalescence times identifies signals of selection and enriched disease heritability. *Nat Genet*. 50(9): 1311–1317 (2018).

46. T. GTE. Consortium, The Genotype-Tissue Expression (GTEx) pilot analysis: Multitissue gene regulation in humans. *Science*. **348**, 648–660 (2015).

47. M. Melé, P. G. Ferreira, F. Reverter, D. S. DeLuca, J. Monlong, M. Sammeth, T. R. Young, J. M. Goldmann, D. D. Pervouchine, T. J. Sullivan, R. Johnson, A. V. Segrè, S. Djebali, A. Niarchou, T. Gte. Consortium, F. A. Wright, T. Lappalainen, M. Calvo, G. Getz, E. T. Dermitzakis, K. G. Ardlie, R. Guigó, The human transcriptome across tissues and individuals. *Science*. **348**, 660–665 (2015).

48. GTEx Consortium, Laboratory, Data Analysis &Coordinating Center (LDACC)—Analysis Working Group, Statistical Methods groups—Analysis Working Group, Enhancing GTEx (eGTEx) groups, NIH Common Fund, NIH/NCI, NIH/NHGRI, NIH/NIMH, NIH/NIDA, Biospecimen Collection Source Site—NDRI, Biospecimen Collection Source Site—RPCI, Biospecimen Core Resource—VARI, Brain Bank Repository—University of Miami Brain Endowment Bank, Leidos Biomedical—Project Management, ELSI Study, Genome Browser Data Integration &Visualization—EBI, Genome Browser Data Integration &Visualization—UCSC Genomics Institute, University of California Santa Cruz, Lead analysts:, Laboratory, Data Analysis &Coordinating Center (LDACC):, NIH program management:, Biospecimen collection:, Pathology:, eQTL manuscript working group:, A. Battle, C. D. Brown, B. E. Engelhardt, S. B. Montgomery, Genetic effects on gene expression across human tissues. *Nature*. **550**, 204–213 (2017).
